# Influence of a six-month home-based individualized physical activity intervention on carotid plaque instability measured by magnetic resonance imaging: a randomized controlled clinical trial

**DOI:** 10.1016/j.eclinm.2025.103158

**Published:** 2025-04-22

**Authors:** Mathilde Mura, Emeraude Rivoire, Leila Dehina-Khenniche, Ghina Jazzar, Sophie Schlatter, Nellie Della-Schiava, Matthieu Arsicot, Zahi A. Fayad, Patrick Lermusiaux, Anne Long, Philippe Douek, Erica N. Chirico, Amandine Thomas, Vincent Pialoux, Antoine Millon

**Affiliations:** a University Claude Bernard Lyon 1, LIBM UR 7424, Team Atherosclerosis, Thrombosis and Physical Activity, Lyon, France; b Vascular Medicine Department, Edouard Herriot Hospital, Hospices Civils de Lyon, Lyon, France; c Vascular and Endovascular Surgery Department, Louis Pradel Hospital, Hospices Civils de Lyon, Lyon, France; d High Fidelity Medical Simulation Center, SIMULYON, Lyon, France; e Research on Healthcare Performance (RESHAPE), INSERM U1290, Lyon 1 University, University of Lyon, Lyon, France; f Electrical Engineering and Ferroelectrical Laboratory, INSA Lyon, Lyon, France; g BioMedical Engineering and Imaging Institute, Icahn School of Medicine Mount Sinai, New York, NY, USA; h Radiology Department, Louis Pradel Hospital, Hospices Civils de Lyon, Lyon, France; i Department of Biomedical Sciences, Cooper Medical School of Rowan University, Camden, NJ, USA

**Keywords:** Cardiovascular diseases, Carotid atherosclerosis, Intraplaque haemorrhage, Physical activity, mHealth, Connected devices

## Abstract

**Background:**

Management of asymptomatic patients with carotid atherosclerotic plaque is still debated. Intraplaque haemorrhage measured by magnetic resonance imaging is the best *in-vivo* indicator of plaque instability and ischaemic risk. A cross-sectional study reported that physical activity was associated with lower prevalence of carotid intraplaque haemorrhage. The aim of this trial was to determine the influence of a physical activity intervention on plaque instability in asymptomatic patients with carotid stenosis.

**Methods:**

*Physical Activity and Carotid Atherosclerotic Plaque haemorrhage* is a randomized clinical trial (ID-RCB:2019-A01543-54/SI:19.06.21.40640; registered on clinicaltrials.govNCT04053166). Patients were enrolled at University Hospital of Lyon, France, from December 2019 to March 2022. Fifty-six asymptomatic patients with carotid artery stenosis ≥50% NASCET were included and randomized 1:1 in an interventional physical activity arm or a control arm. The interventional arm underwent 6 months of an individualized home-based physical activity program, while the control arm followed usual care. The main outcome is the variation of the intraplaque haemorrhage score measured with high-resolution magnetic resonance imaging of the carotid plaque. All data were analysed with an intention to treat. Investigators were blinded from grouping.

**Findings:**

Out of 52 patients participating in the trial, 26 were allocated in each arm. The intraplaque haemorrhage score was significantly reduced over time in the physical activity arm (estimate difference: −0.32 ± 0.15, [95% CI −0.63 to −0.01], p = 0.04). The physical activity arm had increased daily step counts at the end of the 6-month intervention compared to the control arm (1843 ± 758, [CI95% 313–3373], p = 0.02).

**Interpretation:**

This trial demonstrates that an individualized home-based physical activity intervention could reduce the severity of intraplaque haemorrhage detected by magnetic resonance imaging and that it is feasible in asymptomatic patients with carotid atherosclerotic plaque. These results suggest that this type of moderate-intensity physical activity could be included in this population to the best medical treatment.

**Funding:**

This study was funded by the 10.13039/501100003100*Fédération Française de Cardiologie* and *Nouvelle Société Francophone d’Athérosclérose*.

## Graphical abstract

Fifty-two asymptomatic patients with carotid atherosclerosis were randomised 1:1 in physical activity or control group. At the end of the 6 months, intraplaque haemorrhage was reduced in the physical activity group while it stayed stable in the control group. The decreased intraplaque haemorrhage can reduce ischemic stroke incidence.

**Figure:**
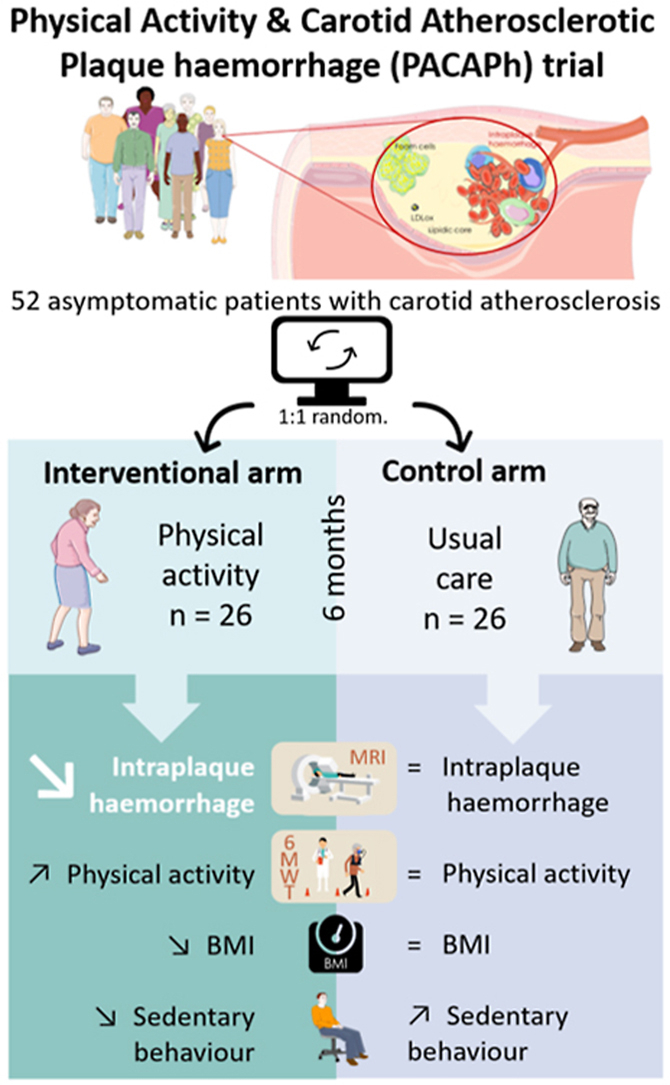

**Research in context**

**Evidence before this study**

According to the World Health Organization, strokes are the second leading cause of mortality worldwide in industrialized countries. Unstable carotid atherosclerotic plaque presenting intraplaque haemorrhage (IPH) are a major cause of ischaemic stroke. High-resolution magnetic resonance imaging (HR-MRI) of the carotid plaque is considered the most appropriate non-invasive tool for monitoring plaque instability, as it is currently the most accurate imaging modality for detecting IPH. In most asymptomatic patients, surgical intervention appears to be inadequate in terms of the benefit-risk ratio. Therefore, it is highly likely that more and more patients will be recommended for medical treatment over surgery. Currently, optimal medical therapy is associated with drug therapy and management of quality of life, including physical activity. The sole drug therapy exhibits positive yet limited results in terms of cardiovascular prevention in this population. Thus, physical activity promotion is of major interest for patients not undergoing surgery.

**Added value of this study**

This trial demonstrates that moderate-intensity physical activity intervention, which was mainly based on walking, can reduce IPH measured by HR-MRI. This type of study is well accepted by the patients, with a 96% adhesion rate to the physical activity intervention, and an increase of distance walked during the 6-min walk test.

**Implications of all the available evidence**

The present results strengthen the previous conclusions that there is an association between moderate physical activity in this population and the recommendation that physical activity should be considered as a complementary therapy for patients who do not require surgery.

## Introduction

After 65 years of age, 4% of men and 2% of women present with carotid stenosis larger than 50% NASCET (North American Symptomatic Carotid Endarterectomy trial), and the prevalence increases up to 7% and 4%, respectively, for the 75–79 year age group.^1^^,^^2^ Vulnerable carotid atherosclerotic plaques present with a thin fibrous cap, a large lipid core, and immature neovascularization leading to intraplaque haemorrhage (IPH).^3^ Among these features, IPH can be reliably assessed by magnetic resonance imaging (MRI) with a high sensitivity and specificity and is the best indicator of plaque instability and subsequent ischaemic risk.^4^, ^5^, ^6^, ^7^ Despite considerable improvement in the understanding of carotid atherosclerotic plaque, the management of asymptomatic patients is still debated.^8^ The Asymptomatic Carotid Artery Stenosis and Asymptomatic Carotid Surgery Trial studies did not report better outcomes after carotid endarterectomy surgery compared to drug therapy for the majority of asymptomatic patients.^9^ Moreover, a recent meta-analysis showed that carotid endarterectomy and modern best medical therapy presented with the same odds of stroke.^10^ Previously, the decision to perform carotid endarterectomy surgery was almost exclusively based on the severity of the carotid stenosis (≥70% NASCET) regardless of the risk of stroke, whereas now plaque vulnerability is also considered.^8^ Thus, the identification of patients at low risk of stroke for whom carotid endarterectomy surgery might be less beneficial^8^ led to a growing population of patients with carotid stenosis >50% NASCET. These patients were only treated according to the best medical therapy which corresponds to the association of statins, anti-hypertensive, and antiplatelet aggregator drugs as well as lifestyle management.

A previous cross-sectional study demonstrated that patients with moderate to high physical activity levels (>900 Metabolic Equivalent of Task [MET].min/week) had fewer haemorrhagic carotid plaques than those with low physical activity levels (<900 MET.min/week).^11^ Considering these results^11^ as well as the benefits of physical activity on atherogenesis and ischaemic risk,^12^ it could be relevant to include physical activity as a best medical therapy in patients with carotid stenosis >50% NASCET when carotid endarterectomy surgery is not recommended. However, to our knowledge, no study has evaluated the effect of a physical activity intervention on the risk of plaque rupture, and more specifically on IPH in patients with carotid stenosis >50% NASCET. In addition, compared to rehabilitation centre programs, home-based physical activity interventions have shown better adherence rates in older individuals^13^ and equal efficacy in patients who have undergone cardiac surgery.^14^ In the context of home-based interventions, the use of connected wearable devices was shown to further improve adherence to physical activity^15^; as well as tailor physical activity based on the patients’ current physical capacities.

The Physical Activity and Carotid Atherosclerotic Plaque haemorrhage (PACAPh) trial^16^ aims to test the impact of a 6-month home-based individualized physical activity intervention using a connected wearable device, on carotid atherosclerotic plaque instability features measured by MRI as primary endpoint.

We hypothesized that the 6-month intervention in physical activity would i) reduce the IPH ii) improve physical function and iii) improve other risk factors of plaque vulnerability.

## Methods

### Ethics

The data that support the findings of this trial are available from the corresponding author upon reasonable request. The PACAPh protocol has been approved by a national ethics committee (Comité de Protection des Personnes Sud-Méditerranée II ID-RCB:2019-A01543-54/SI:19.06.21.40640), has been registered on clinicaltrials.gov (NCT04053166). This article follows the CONSORT guidelines. All patients gave their consent after a period of reflection following the provision of information.

### Trial design

The protocol for the PACAPh trial, a randomized, controlled, single-centre trial, has been previously described.^16^ Patients were enrolled in the vascular surgery unit of the Hospices Civils de Lyon, France (centre of ethic approval).

### Patients

Asymptomatic patients with carotid atherosclerotic plaques detected by Doppler ultrasonography exam, with stenosis ≥50% NASCET, and without carotid endarterectomy surgery indication according to European guidelines were included. The threshold of 50% NASCET was chosen in order to have plaques large enough to be visible on MRI but not eligible for surgery. The main exclusion criteria were: ischaemic event <6 months, intercurrent inflammatory diseases, and major contraindication to physical activity (e.g., advanced arthritis). Based on the results of Mury et al., 2019, ^11^ the intended samples size was calculated using G∗power for between-factor repeated measures. An *a priori* power calculation based on a medium effect size (f = 25, α = 5%, 1−β = 0.85) resulted in the requirement of 74 patients. Considering an 8% attrition rate, we will aim to recruit 80 patients. All patients gave informed consent.

### Arms

As part of their usual care, all patients were treated in regard to their cardiovascular risk factors and received cardiovascular lifestyle guidelines, including nutrition and smoking cessation recommendations at inclusion. According to the medical chart and confirmed by the patient, medical treatment was unchanged during the trial period.

#### Physical activity intervention arm

Patients randomized into the physical activity intervention arm wore a connected activity tracker (Fitbit AltaHR, CA, USA) throughout the 6-month intervention period. The physical activity goal for this trial was an average of 7000 steps/day or a 30% increase in the average steps/day count (compared to the first two weeks of the intervention). In order to achieve this goal, a physical activity instructor called the patients every 14 days to propose a personalized daily step goal and to provide them with individualized advice (e.g., increase their moderate physical activity; for more details, see^16^).

#### Control arm

Patients randomized into the control arm wore the activity tracker for the first 14 and last 14 days of the trial period. Daily steps were not monitored during the 6-month period in the control arm in order to avoid the incentive effect of the connected devices on physical activity and prevent potential inter-arm contamination.

### Randomization

**Fig. 1:**
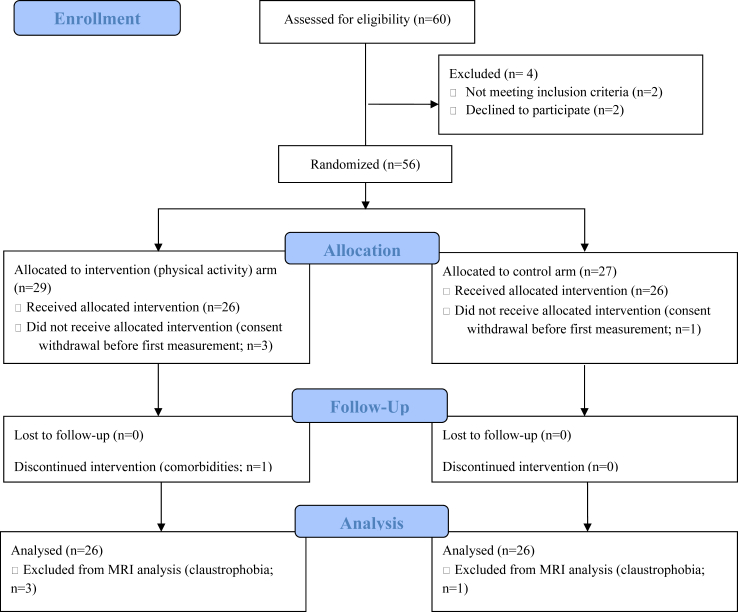
Flow chart diagram from enrolment to analysis of the Physical Activity and Carotid Atherosclerotic Plaque haemorrhage (PACAPh) trial. MRI: magnetic resonance imaging.

Patients were randomly assigned in a 1:1 ratio either in the physical activity (interventional) arm or the control arm (usual clinical care) without stratification, using the Ennov Clinical© software (Fig. 1). See arms characteristics at inclusion in Table 1.

**Table 1:** Patient characteristics at inclusion.

	Physical activity arm (n = 26)	Control arm (n = 26)	p-value
Sex (women/men)	10 (38%)/16 (62%)	9 (35%)/17 (65%)	0.77
Age (years)	70.73 ± 8.05	71.69 ± 8.77	0.68
Weight (kg)	75.10 ± 10.24	73.00 ± 15.08	0.56
Body mass index (kg/m^2^)	27.18 ± 4.66	26.05 ± 4.04	0.89
Waist circumference (cm)	99.39 ± 11.60	96.54 ± 11.98	0.81
Hip circumference (cm)	103.32 ± 10.42	101.94 ± 9.27	0.62
Fat percentage (%)	37.45 ± 6.12	36.89 ± 5.62	0.73
Tobacco use (pack-year)	25.63 ± 36.41	17.9 ± 20.16	0.50
Smoking status:			
Never smoked	6 (23%)	9 (35%)	0.36
Former smoker	17 (65%)	13 (50%)	0.26
Current smoker	3 (12%)	4 (15%)	0.68
GPAQ (MET.min/week)	3088 ± 2995	3342 ± 5096	0.16
Sedentary behaviour (min/day)	536.54 ± 229.19	489.52 ± 199.34	0.46
Nutrition (score)	37.62 ± 8.36	37.54 ± 7.6	0.97
Quality of life (score)	65.50 ± 13.96	71.35 ± 17.06	0.22
Stenosis (% NASCET)	60.38 ± 9.05	64.81 ± 10.15	0.12
Stenosis laterality (R/L)	18 (69%)/8 (31%)	13 (50%)/13 (50%)	0.16
Contralateral stenosis (n)	8 (31%)	6 (23%)	0.88
Resting heart rate (bpm)	69.54 ± 13.49	72.00 ± 14.71	0.53
Resting systolic pressure (mmHg)	148.81 ± 22.55	148.35 ± 24.37	0.94
Resting diastolic pressure (mmHg)	84.35 ± 9.06	82.38 ± 10.79	0.16
**Diagnosed comorbidities**			
Hypertension (n)	21 (82%)	22 (85%)	0.71
Diabetes mellitus (n)	11 (42%)	16 (62%)	0.17
Dyslipidaemia (n)	18 (69%)	21 (82%)	0.34
Coronaropathy (n)	7 (27%)	8 (31%)	0.78
LE-PAD (n)	6 (23%)	7 (27%)	0.75
Retinal thrombosis (n)	1 (4%)	0 (0%)	0.31
Ancient stroke or TIA (n)	3 (12%)	4 (15%)	0.68
Myocardial infarct (n)	1 (4%)	1 (4%)	1.00
Family history of ischaemia (n)	5 (19%)	7 (27%)	0.51
**Drug treatment**			
Anti-platelet (n)	20 (77%)	24 (92%)	0.14
Anti-hypertensive (n)	19 (73%)	21 (81%)	0.64
Statins (n)	20 (77%)	23 (88%)	0.57
Best medical treatment (n)	13 (50%)	19 (73%)	0.10
Diabetes drugs (n)	10 (38%)	13 (50%)	0.39
Other treatments (n)	20 (77%)	17 (65%)	0.18

Data are presented as mean ± standard deviation or number (percentage). Variables are compared using Student t-test, Wilcoxon, or Pearson's Chi-squared Test as appropriate. All p-values were p > 0.05. GPAQ: global physical activity questionnaire; LE-PAD: lower extremities peripheral artery disease; MET: metabolic equivalent of task; TIA: transient ischaemic attack.

### Procedures

All the following outcomes were measured once at inclusion and once at the end of the trial period (after 6 months).

#### Primary outcome: plaque magnetic resonance imaging

Imaging was performed on a 3–Tesla high-resolution MRI (Ingenia scanners, Philips Healthcare, Best, The Netherlands) with a dual surface coil (SenseFlexS; Philips Healthcare). Correct positioning was ensured by a radiologist in order to get a signal-to-noise ratio identical during both examinations. If the bifurcation of the carotid artery was too deep in the neck, an ultrasound was used to place the markers in line with the bifurcation of the artery in order to properly position the coils. Time of flight sequence was acquired and centred on the plaque perpendicular to the main carotid axis. Contrast-enhanced magnetic resonance angiographic and T1–weighted images were obtained at the same levels and with the same parameters before and during an injection of 30 mL of a gadolinium-based contrast agent (ProHance®, Bracco Imaging S.P.A., Colleretto Giacosa, Italy). Each image was assessed for all parameters (quality, IPH score and presence, fibrous cap integrity, calcifications and lipid core presence) by two experts who were blinded to the experimental arm, the time of assessment, and trial goals.

First, image quality was rated on a 5-point scale: images with a quality score ≥3 were included in the analysis.

Second, IPH score and severity were assessed from the IPH signal intensity and longitudinal length on the T1–weighted sequence (see Table 2 for score details determination).^17^ Scale was rated 0 when IPH was absent (i.e., no difference with the sternocleidomastoid muscle intensity signal). When IPH was detected it was attributed a scale of 1, 2, or 3 depending on the signal intensity (i.e., >2 times visual difference with the sternocleidomastoid muscle intensity signal, mean signal intensity on all slides where IPH was present; see Supplemental file 1) and signal longitudinal length (calculated according to the number of consecutive 2 mm-slides presenting hyperintensity; see Supplemental file 2). A length threshold of 12 mm and a signal-to-muscle ratio threshold of 5 were established according to Wang and collaborators.^17^ The scale was then established as described in Table 2. The IPH score was the mean of the scaling assessed by the two independent experts (NDS and AM). The agreement on the IPH scale between the two experts was good (Cronbach α = 0.71, CI [0.66: 0.81]). In order to improve the quality of the assessments, images for which the experts' scaling was >1 were discussed with a third expert (PD), until they reached a consensus and gave the same scale to the images. In addition, the overall absence or presence of IPH was classified (i.e., no difference vs. visual difference with the sternocleidomastoid muscle intensity signal).^18^^,^^19^ Thrombus was not recorded.

**Table 2:** Table of intraplaque haemorrhage severity scaling.

	IPH length ≤ 12 mm	IPH length > 12 mm
IPH signal intensity ≤5 times SCM signal intensity	1	2
IPH signal intensity >5 times SCM signal intensity	2	3

If no intraplaque haemorrhage was detected, the plaque was scaled as 0 (signal-to-muscle ratio <2). If IPH was detected, the following table was used to scale the IPH severity.

If IPH was detected IPH longitudinal length and plaque signal intensity were scaled 1 to 3 according to the threshold determined as mean IPH length and mean IPH signal to sternocleidomastoid muscle signal as measured in asymptomatic plaques in Wang et al.^17^ IPH shorter than 12 mm and with an IPH-to-muscle ratio comprised between 2 and 5 was scaled 1; IPH shorter than 12 mm and with an IPH-to-muscle ratio >5 OR an IPH longer than 12 mm but with an IPH-to-muscle ratio comprised between 2 and 5 was scaled 2; IPH longer than 12 mm and with an IPH-to-muscle ratio >5 was scaled 3. IPH: intraplaque haemorrhage; SCM: sternocleidomastoid muscle.

Calcifications were identified as an asignal on the time of flight, T1-weighted and post-gadolinium T1-weighted sequences. Lipid core was identified as an iso-intense signal on Time of flight and T1-weighted sequences. While fibrous cap rupture was observed as a discontinuity in the post gadolinium T1-weighted images or/and a disrupted or an asignal band adjacent to the lumen on Time of flight images. Calcifications and lipid core presence were rated 0 (absent), 1 (minor), or 2 (major). Fibrous cap was rated as 0 (ruptured) or 1 (intact).

#### Secondary parameters’ analysis

##### Step counts

Daily step counts were measured using the Fitbit AltaHR wearable device. In the physical activity intervention arm, daily steps were monitored during the 6-months period. In the control arm, daily steps were recorded the first 14 and last 14 days of the trial period but were not monitored during the 6-months period in order to avoid the incentive effect of the connected devices on physical activity^20^ and prevent inter-arm contamination.

##### Physical fitness

Walking endurance was measured by the total distance walked during the 6-min walk test. Oxygen uptake was recorded continuously using Metamax3b® analyser (Cortex Biophysik, Leipzig, Germany).

##### Clinical data

Clinical data included age, sex, resting heart rate, resting systolic and diastolic blood pressures, and percentage of stenosis. Any previous or current conditions were recorded: acute coronary events, arterial retinal thrombosis, stroke, transient ischaemic attack, myocardial infarction, peripheral arterial disease of the lower limbs, hypertension, diabetes, dyslipidaemia, familial related medical history and/or drug treatment. Best medical treatment comprising anti-platelet, anti-hypertensive and statin, was also recorded. Any adverse events were also reported.

##### Metabolic syndrome parameters

The anthropometric characteristics of height, weight, body mass index, skinfold, and waist and hip circumference were collected. Fat percentage was calculated using the skinfold sum method equations.^21^ A blood sample was drawn into an ethylenediaminetetraacetic acid tube after an overnight fast and a sufficient period of time after any acute anti-inflammatory treatment, in order to determine lipid content, complete blood count, and fasting glucose content.

##### Questionnaires

The Folstein mini-mental questionnaire^22^ allowed to assess whether the patients had sufficient cognitive capacities (score ≥8) to answer the further questionnaires. At inclusion, the weekly physical activity levels and intensity were assessed by the Global Physical Activity Questionnaire^23^ and expressed in MET-min/week. The daily amount of time spent in a sedentary behaviour was assessed by the Sedentary Behaviour Questionnaire^23^ expressed in min/day. Nutrient intakes were evaluated using the French *Programme National Nutrition Santé* questionnaire.^24^ Quality of life was assessed using the EuroQol-5Dimensions-5Levels questionnaire. Tobacco consumption was evaluated in pack-year.

### Outcomes

The primary outcome was the IPH score variation over time. Secondary outcomes were the IPH prevalence, scores of calcifications and lipid core volume, integrity of fibrous cap, daily step counts, distance on the 6-min walk test, blood pressures, body mass index (BMI), lipid profile, and questionnaire scores measuring cognitive capacities, sedentary behaviour, nutrient intake, and quality of life variation over time. The experimenters who measured the outcomes were blinded to the arm of randomisation.

### Statistical analysis

All hypotheses were tested using a statistical significance level of 0.05. Data were analysed using the R software (version 4.1.2, R Foundation for Statistical Computing, Vienna, Austria).

At inclusion, qualitative data were described by count and percentage, and analysed with Pearson’s χ^2^ test. Quantitative data were described by mean values and standard deviations, and after visual inspection of the qq plots and histogram, analysed with Wilcoxon or t-test when appropriate.

All the outcomes were analysed on an intent-to-treat basis. To analyse the effect of the intervention on the dependent variables (MRI results, physical activity levels, and metabolic parameters), we first used a linear mixed effects model with a by-subject random intercept (Model 1) and entered time (PRE/POST) and arm (physical activity/control) as fixed effects with interaction terms (lme function, nmle package). As post-hoc investigations, we used planned contrasts (emmeans package). An additional multivariate linear regression model, further adjusted for sex, age, diabetes status (yes/no), smoking status (yes/no) and variable value at inclusion assessed the influence of the physical activity intervention compared to the control arm on each variable value at the end of the intervention (Model 2). Expect if stated otherwise, estimate differences of the model 2 are presented in the main text, they always take the control group variation as a reference. The difference in the proportion of IPH presence (yes/no) was assessed using a generalised linear mixed-effects model with a by-subject random intercept and entered time (PRE/POST) and arm (physical activity/control) as fixed effects with interaction terms and with binomial response distribution and a logit link function.

### Role of funding source

This trial was funded by the *Fédération Francaise de Cardiologie and Nouvelle Société Francophone d’atherosclérose*, but the funders did not interfere in the study design, data collection, outcome measurements, analysis, interpretation, manuscript writing nor in submission choices.

## Results

Patients were included from Dec 3, 2019, to Mar 4, 2022. Although the initial aim was to recruit 80 patients,^16^ a total of 60 patients presenting with carotid stenosis ≥50% NASCET were screened in our centre. This low inclusion rate was primarily due to the COVID-19 pandemic, during which consultation for the service were limited to urgent care, thus the targeted population was not prioritized. Two patients refused to participate, one was excluded because of cancer diagnosis, and one was unable to perform physical activity. A total of 56 patients (22 women, 71 ± 8 y/o, range 53–89 y/o) were included. Four patients retracted their consents before the first examination, thus the following analyses were conducted on the remaining 52 patients (i.e., no missing data, except when stated otherwise; Fig. 1). Arms were equivalent in characteristics, diagnosed comorbidities and drug treatments at inclusion (Table 1).

As four patients refused to undergo MRI due to claustrophobia, MRI analyses were performed on 48 patients. All MRI scans obtained a quality score ≥3. At inclusion, 69% of patients presented with an IPH (IPH scale > 0).

**Fig. 2:**
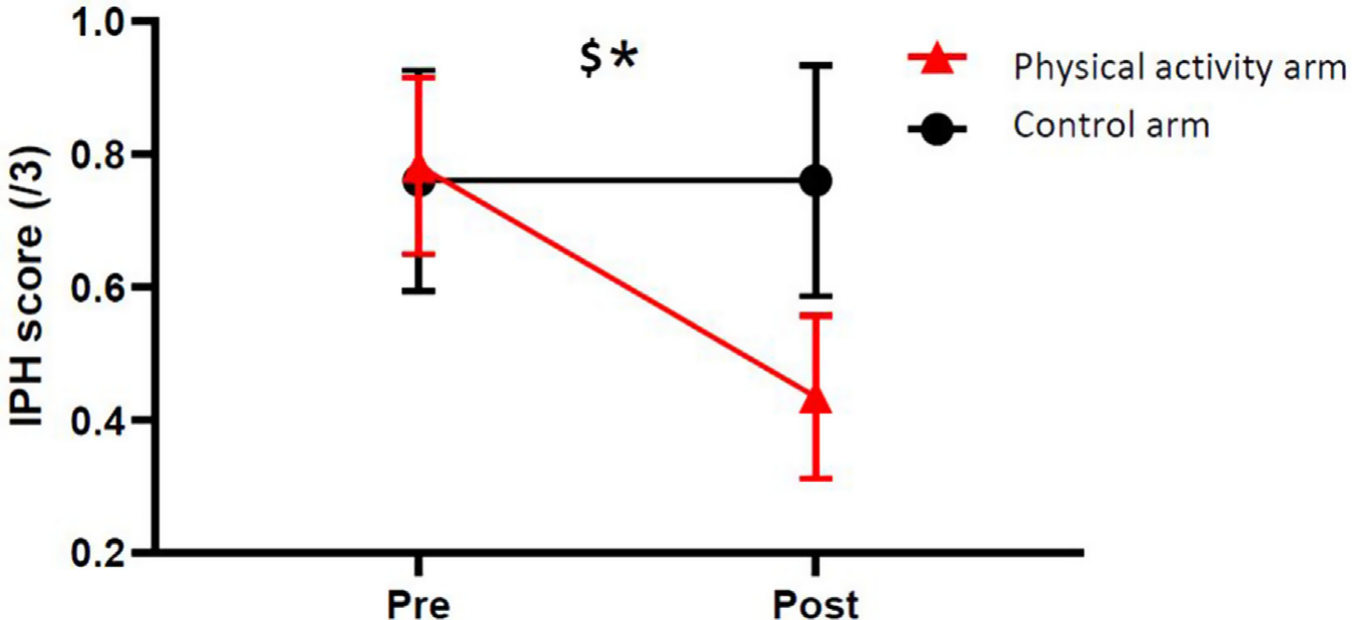
Change in IPH score between the pre- and post-trial periods, measured on T1-weighted MRI sequence. IPH score was scaled from 0: no IPH to 3: important IPH by visual comparison with the sternocleidomastoid muscle signal and IPH score was calculated as the mean of each scales in each arm at each time, see Table 2 and Appendix 1 and 2. IPH: intraplaque haemorrhage; MRI: magnetic resonance imaging; PRE: measurement at inclusion and POST: measurement at the end of the trial. ∗p ≤ 0.05 significant contrast Time∗Arm, in non-adjusted linear mixed mode (Model 1). $p < 0.05 significant Model 2 adjusted on tobacco consumption, diabetes, age, sex, and IPH score at inclusion.

The adjusted multivariate model showed that, compared to the control arm, the physical activity arm was characterized by a lower IPH score following the intervention (−0.32 ± 0.15, [95% CI −0.63 to −0.01], p = 0.04; Fig. 2 and Supplemental data 3). The IPH score was reduced over time in the physical activity arm (−0.35, equivalent to a 43% reduction), while it remained stable over time in the control arm (0.00, equivalent to a 0% reduction). In addition, the number of patients presenting IPH reduced over time in the physical activity arm, while it remained stable in the control arm (−2.3 ± 1.19, [95% CI −4.64 to 0.04], p = 0.050) (Table 3). The calcification score (0.12 ± 0.17, [95% CI −0.23 to 0.46], p = 0.50), lipid core volume (0.24 ± 0.14, [95% CI −0.05 to 0.53], p = 0.10), and fibrous cap integrity (−0.04 ± 0.13, [95% CI −0.30 to 0.20], p = 0.71) were not significantly modified over time in either arm (Supplemental data 4).

**Table 3:** Prevalence and score of intraplaque haemorrhage according to scale measured on T1–weighted MRI sequence.

	Physical activity arm (n = 23)		Control arm (n = 25)		p-value
	Pre	Post	Pre	Post	
IPH absence, score 0 (n)	5	12	10	9	0.05^a^
IPH presence (n)	18	11	15	16	
IPH score 0.5 (n)	7	6	5	6	
IPH score 1 (n)	6	2	3	5	
IPH score 1.5 (n)	4	2	2	2	
IPH score 2 (n)	0	1	4	0	
IPH score 2.5 (n)	1	0	1	2	
IPH score 3 (n)	0	0	0	1	

Number of patients for each score of IPH. IPH score is the mean scale obtained from evaluation by two independent experts. Statistical model was applied comparing the absence (scale 0) and the presence (any scale between 0.5 and 3). MRI: magnetic resonance imaging; Pre: measurement at inclusion; Post: measurement at the end of the trial period.

a p ≤ 0.05 significant difference between group evolution over time in generalised linear mixed-effects model with binomial response distribution and a logit link function.

**Fig. 3:**
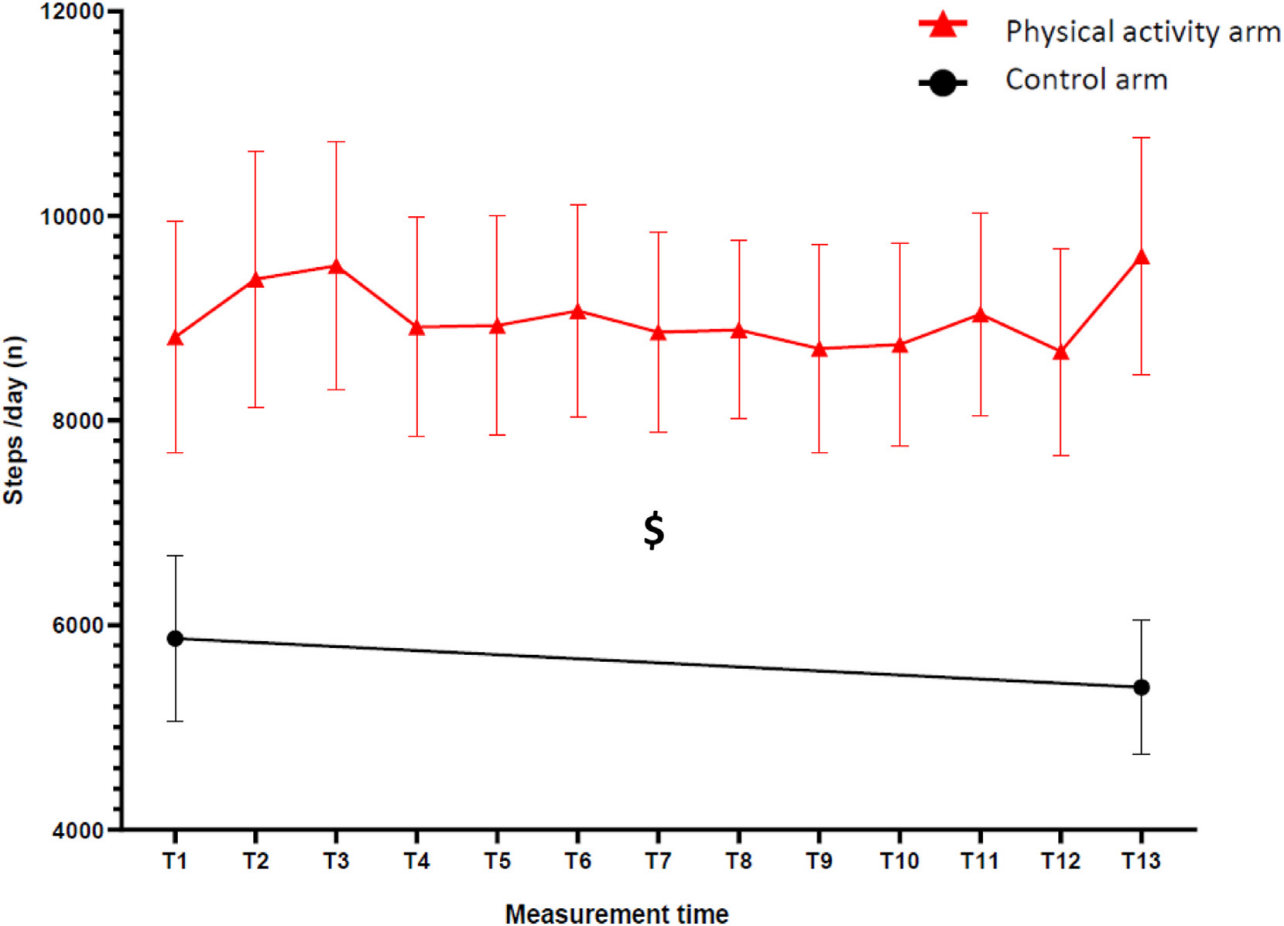
Change in daily step count during the intervention, the daily step count was expressed as a mean (± standard error) at each measurement. In the physical activity arm, a mean of daily steps was measured every two weeks during phone calls (T1 to T13). In the control arm, the daily step count was measured during the first two weeks after inclusion and during the last two weeks of the trial (i.e., T1 and T13). $p < 0.05 significant Model 2 adjusted on tobacco consumption, diabetes, age, sex, and steps/day at T1.

The daily step count was higher after the first 14 days of the trial period (+50%) and during the last 14 days of the trial period (+78%) in the physical activity arm compared to the control arm (estimate arm difference with the control arm in model 1: 3060 ± 1262, [95% CI −229 to 5103], p = 0.01). Over time, the adjusted model (Model 2) showed an increased daily step count in the physical activity arm while it slightly decreased in the control arm (1843 ± 758, [CI95% 313–3373], p = 0.02, Fig. 3). The distance walked during the 6-min walk test increased in the physical activity arm (432 ± 129 m to 457 ± 133 m) while it remained stable in the control arm (401 ± 120 m to 399 ± 130 m) (estimate difference: 32 ± 12, [95% CI 7–56], p = 0.01; Table 4).

**Table 4:** Variables measured during the 6 MWT in the intervention (PA) and the control arms at inclusion and at the end of the trial period.

	PA pre (n = 26)	PA post (n = 26)	CTRL pre (n = 26)	CTRL post (n = 26)	p-value	
					Model 1	Model 2
6 MWT distance (m)	432 ± 129	457 ± 133	401 ± 120	399 ± 130	**0.01**^a^	**0.01**^b^
% predicted distance (%)	100 ± 26	107 ± 27	96 ± 27	95 ± 28	**0.01**^a^	**0.02**^b^
Energetic cost of walking (mL/kg/m)	0.21 ± 0.06	0.20 ± 0.02	0.19 ± 0.04	0.20 ± 0.04	0.58	0.74
Absolute VO_2_ plateau (L/min)	1.07 ± 0.41	1.21 ± 0.36	0.98 ± 0.48	1.06 ± 0.49	0.41	0.46
Relative VO_2_ plateau (mL/min/kg)	14.30 ± 5.11	16.04 ± 4.57	13.30 ± 4.37	14.12 ± 5.12	0.09	0.89
Respiratory exchange ratio	0.81 ± 0.04	0.85 ± 0.08	0.85 ± 0.07	0.83 ± 0.05	**<0.01**^a^	**0.04**^b^
6 MWT Borg’s RPE	3.70 ± 2.55	4.32 ± 2.10	4.32 ± 1.52	4.08 ± 2.50	0.23	0.20
6 MWT heart rate (bpm)	95.8 ± 22.3	103.4 ± 23.1	96.5 ± 18.2	100.6 ± 18.5	0.54	0.43
6 MWT oxygen saturation (%)	96.0 ± 2.2	95.6 ± 2.0	95.1 ± 2.2	95.4 ± 2.2	0.33	¨0.56
6 MWT systolic BP (mmHg)	159.7 ± 126.14	160.1 ± 21.0	160.4 ± 25.4	155.4 ± 27.1	0.48	0.32
6 MWT diastolic BP (mmHg)	86.4 ± 9.3	82.8 ± 12.2	83.3 ± 8.1	82.0 ± 10.2	0.45	0.58

Predicted distance is adjusted for sex, height, age, and weight. CTRL: control arm; PA: physical activity arm; RPE: rate of perceived effort; VO_2_: Oxygen intake; 6 MWT: 6-min walk test.

Clinically relevant relationships in the model are in bold.

a p ≤ 0.05 significant contrast (arm ∗ time) in the linear mixed model.

b p ≤ 0.05 significant Model 2 adjusted on tobacco consumption, diabetes, age, sex and variable value at inclusion.

The BMI decreased in the physical activity arm (27.18 kg/m^2^ to 26.77 kg/m^2^) while it remained stable in the control arm (26.05 kg/m^2^ to 26.17 kg/m^2^) (estimate difference in model 1: −0.49 ± 0.25, [95% CI −0.99 to 0.01], p = 0.048). This difference did not persist after adjustments in Model 2 (−0.15 ± 1.15, [95% CI −2.18 to 2.49], p = 0.89). All metabolic parameters are shown in Table 5.

**Table 5:** Metabolic syndrome parameters in the intervention (PA) and control arms at inclusion and at the end of the trial period, including lipid content and complete blood count.

	PA pre (n = 26)	PA post (n = 26)	CTRL pre (n = 26)	CTRL post (n = 26)	p-value	
					Model 1	Model 2
Cholesterol (mmol/L)	3.90 ± 0.76	3.62 ± 1.16	3.98 ± 1.40	3.94 ± 1.27	0.27	0.14
Triglycerides (mmol/L)	1.16 ± 0.45	1.15 ± 0.48	1.46 ± 0.63	1.38 ± 0.58	0.64	0.81
HDL-cholesterol (mmol/L)	1.27 ± 0.33	1.24 ± 0.38	1.15 ± 0.40	1.27 ± 0.40	0.13	0.88
LDL-cholesterol (mmol/L)	2.05 ± 0.69	1.87 ± 0.89	2.20 ± 0.85	1.95 ± 0.88	0.76	0.91
Fasting glycaemia (mmol/L)	5.79 ± 1.32	5.86 ± 0.96	7.13 ± 2.42 ¤	6.73 ± 1.83	0.10	0.56
BMI (kg/m^2^)	27.18 ± 4.66	26.77 ± 4.46	26.05 ± 4.04	26.17 ± 4.03	**0.05**^a^	0.89
Resting systolic blood pressure (mmHg)	148.81 ± 22.55	140.04 ± 18.13	148.35 ± 24.40	140.72 ± 26.15	0.77	0.69
Resting diastolic blood pressure (mmHg)	84.35 ± 9.06	79.27 ± 10.73	82.38 ± 10.79	77.72 ± 10.91	0.87	0.96

CTRL: control arm; HDL: high-density lipoprotein; LDL: low-density lipoprotein; PA: physical activity arm.

Clinically relevant relationships in the model are in bold.

a p ≤ 0.05 significant contrast (arm ∗ time) in the linear mixed model (Model 1). No significant results for the Model 2 adjusted on tobacco consumption, diabetes, age, sex and variable value at inclusion.

All patients had a score ≥8 on the Folstein mini-mental test. The patients in the physical activity arm spent less time being sedentary after the intervention period (537 ± 229 min/day to 445 ± 171 min/day) while patients in the control arm showed an increase in sedentary time (490 ± 199 min/day to 525 ± 223 min/day) (estimate difference: −119 ± 50, [95% CI −220 to −17], p = 0.02). Quality-of-life was not significantly modified over time in either arm (estimate difference: 6.50 ± 4.50, [95% CI −2.58 to 15.57], p = 0.16).

During the protocol, no patient underwent a stroke or any other cardiovascular event. Although one patient in the physical activity arm stopped the intervention (96% adhesion), all patients (100%) underwent the pre- and post-measurements.

## Discussion

The present trial reports for the first time that an individualized home-based physical activity intervention likely reduces the IPH score in asymptomatic patients with carotid atherosclerotic stenosis. These findings also show that the physical activity intervention increased the distance walked in the 6-min walk test, and also decreased the time spent in sedentary behaviour. These results bring new evidence supporting the efficacy of the therapeutic use of physical activity to diminish the risk of plaque instability in asymptomatic carotid atherosclerotic patients.

To our knowledge, only one study^25^ measured IPH evolution over time using a quantification instead of the presence of IPH. Indeed, Wang and collaborators looked at the persistence of the MRI signal in asymptomatic patients with carotid atherosclerosis for 18 months. They reported a slightly significant MRI T1 hyper signal (same marker of IPH) decrease after 12 months of follow-up, which continued to decrease at the 18-month follow-up, whereas at the 6-month follow-up, no significant decrease was observed. This latter study suggests that IPH can decrease over time. Nevertheless, our findings suggest that physical activity may accelerate this process. The present *in vivo* IPH results measured by MRI confirm previous cross-sectional observations where the carotid IPH prevalence was lower in patients with higher levels of physical activity^11^ and supports the hypothesis of a beneficial effect of moderate-intensity physical activity on IPH. The reduction in IPH score could be due to better regulation of the inflammatory markers and cells. Indeed, IPH can result from a pro-inflammatory environment,^3^^,^^26^ and chronic physical activity is known to regulate inflammation.^27^ These hypotheses should be further tested in future studies. The latest guidelines of the European Society of Vascular Surgery on management of the asymptomatic carotid disease encourage lifestyle management, including an increase in physical activity.^8^ In line with these recommendations, the present results of this study show that physical activity interventions have a direct influence on plaque vulnerability, reducing the IPH score over time. This is confirmed by the adjusted model for sex, age, diabetes, smoking status, and IPH score at inclusion. It should be noted that diet, smoking status, and drug treatment were not modified during the intervention in either arm. Based on this new level of evidence, physical activity should be added to the best medical treatment of asymptomatic patients with carotid stenosis, on the same level as statin treatment (level of evidence B).^9^ The results herein also suggest that the detection of carotid IPH by MRI is sufficiently sensitive to discriminate the effects of physical activity on IPH, strengthening the conclusion that MRI is a reliable tool to assess *in-vivo* the presence and severity of IPH.^4^, ^5^, ^6^, ^7^ Indeed, studies suggest that IPH signal intensity might be related to plaque instability: the more hyperintense, the more it is related to instable/ruptured plaques.^28^^,^^29^ As IPH is a dynamic process, the severity score we propose takes into account IPH longitudinal length and intensity, allowing for a more sensitive follow-up of the IPH evolution. The evaluation of the IPH severity would be a useful tool to define the patients that need carotid endarterectomy and patients that would benefit from the optimal medical therapy in association with a physical activity.

The efficacy of the physical activity intervention proposed herein was demonstrated by an increase in the distance walked during the 6-min walk test and a decrease in time spent in sedentary behaviour in the physical activity arm. The increase in 6-min walk test distance in the physical activity arm was within the range of minimal clinically important difference for patients with chronic diseases.^30^ These results support the efficacy of moderate-intensity physical activity intervention in such patients, and more generally, the beneficial effects of physical activity on atherosclerosis^12^ and its risk factors,^31^ especially in patients with asymptomatic carotid plaque. The present trial also demonstrates the feasibility of implementing an effective home-based individualized physical activity intervention based on walking and using connected devices in asymptomatic patients with carotid stenosis. In addition to the excellent adhesion rate, 96% of the patients reached the final physical activity’s objective (i.e., reach 7000 steps/day or increase the daily step count by 30%) which is higher than a similar physical activity (duration and intensity) but centre-based intervention in patients with cardiovascular diseases (75%–94%).^32^^,^^33^ To note, a per-intervention analysis (data not shown) was conducted with similar results to the per-protocol analysis. The adhesion rate reported herein shows that home-based physical activity interventions using connected wearable devices may increase adherence to physical activity as previously suggested.^13^, ^14^, ^15^ In intention-to-treat, only 50% of the patients in the physical activity arm took the recommended best medical therapy, and 12% of them continued smoking against medical recommendations. The adherence to the home-based physical activity intervention proposed herein thus reinforces the interest of including physical activity as a best medical treatment for this specific population of older inactive patients, with carotid stenosis and cardiovascular comorbidities. This strong adherence also suggests that such moderate physical activity intensity intervention is easy to follow. During the protocol, the daily step count increased in the physical activity arm (+10%), while it decreased and remained lower in the control arm, underlining the compliance to the physical activity intervention throughout the 6 months. However, long-term sustainability and post-program physical activity level observance without regular third-party management (i.e., bi-monthly phone calls in the present study) remains unknown and should be investigated in future studies.

This trial has some limitations and strengths. First, this trial was done in a real-life setting. As such, visual quantification of MRI images was chosen as it represents an easily available tool in routine practice and is currently the most frequent technique used by radiologists in order to assess IPH. Although the analysis of plaque IPH severity using visual segmentation shows good agreement with automatic segmentation, such visual segmentation is more subject to lower inter-observer reproducibility.^34^ In order to limit this bias, all segmentations were done by the two same experts, with a good inter-observer reproducibility. Moreover, the analysis of MRI signal was done on T1 sequences, future studies might confirm our finding by using a Spectral Presaturation with Inversion Recovery (SPIR) sequences which are less sensitive to flux (i.e., blood flow). One should know that the study was stopped before the *a priori* sample size (n = 80)^16^ was reached. Due to COVID pandemic, the recruitment rate was slower than expected, as previously acknowledged. Consequently, it should be acknowledged that our results, although significant, could be subjected to type I error. Furthermore, it should be also noted that the primary outcome analysed using the pre-specified model (i.e., effect of the physical activity intervention on IPH by the non-adjusted linear mixed effects model; model 1) are borderline (p = 0.05). Thus, the generalizability of the present results needs to be assessed by a larger multicentre trial, with more power. As per law, ethnicity could not be recorded, which could be another confounding factor of our results. A new trial could also assess the long-term risk of stroke and the long-term sustainability of this type of physical activity intervention. On the other hand, the real-life setting of this trial, allows to conclude that this type of intervention is easy to implement in the patients’ care path. Unless other multimodal imaging classifications^35^ our approach is more cost- and time-effective as well as less constraining for the patient, as it uses a single imaging examination. Moreover, due to the use of connected devices and regular but remote monitoring by a professional in adapted physical activity, the feasibility of the present home-based individualized physical activity intervention only requires low human and economic resources.

In conclusion, we demonstrated that an individualized home-based physical activity intervention is feasible in asymptomatic patients with carotid atherosclerotic plaque and that it may reduce the severity of IPH, a well-established risk factor of ischaemic stroke. This intervention also increased the 6-min walk test distance, and reduced sedentary behaviour, underlining its multidimensional effectiveness in these patients. These results should be confirmed by a larger trial.

## Contributors

Conceptualisation: MM, PV, MA; Methodology: MM, DKL, PV, MA; Validation: PV, MA; Formal analysis: MM, SS, PV; Investigation: MM, RE, JG, DKL; Resources: RE, DSN, AM, LP, LA, DP, MA; Data curation: DKL; Data verification: MM, LDK, VP; Writing—original draft: MM; Writing—Review & editing: MM, RE, DKL, JG, SS, DSN, AM, FZA, LP, LA, DP, CEN, TA, PV, MA; Visualization: MM; Supervision: MM, DKL, PV, MA; Project administration: MM, DKL, MA; Funding acquisition: MA. All authors read and approved the final version of the manuscript.

## Data sharing statement

Data are available upon reasonable request to the corresponding author.

## Declaration of interests

The authors declare no conflict of interest (see COI disclosure).

## Appendix ASupplementary data

Supplemental files 1–4

Statistical plan

RIPH 1 et 2 PACAPH english

Renamed_ef9bb
