# Supplementary material for: Influence of a six-month home-based individualized physical activity intervention on carotid plaque instability measured by magnetic resonance imaging: a randomized controlled clinical trial
Source: eClinicalMedicine. 2025 Apr 22;83:103158. doi: 10.1016/j.eclinm.2025.103158 (PMC12179388; doi:10.1016/j.eclinm.2025.103158)
Supplement: Supplemental files 1–4 [file mmc1.docx]

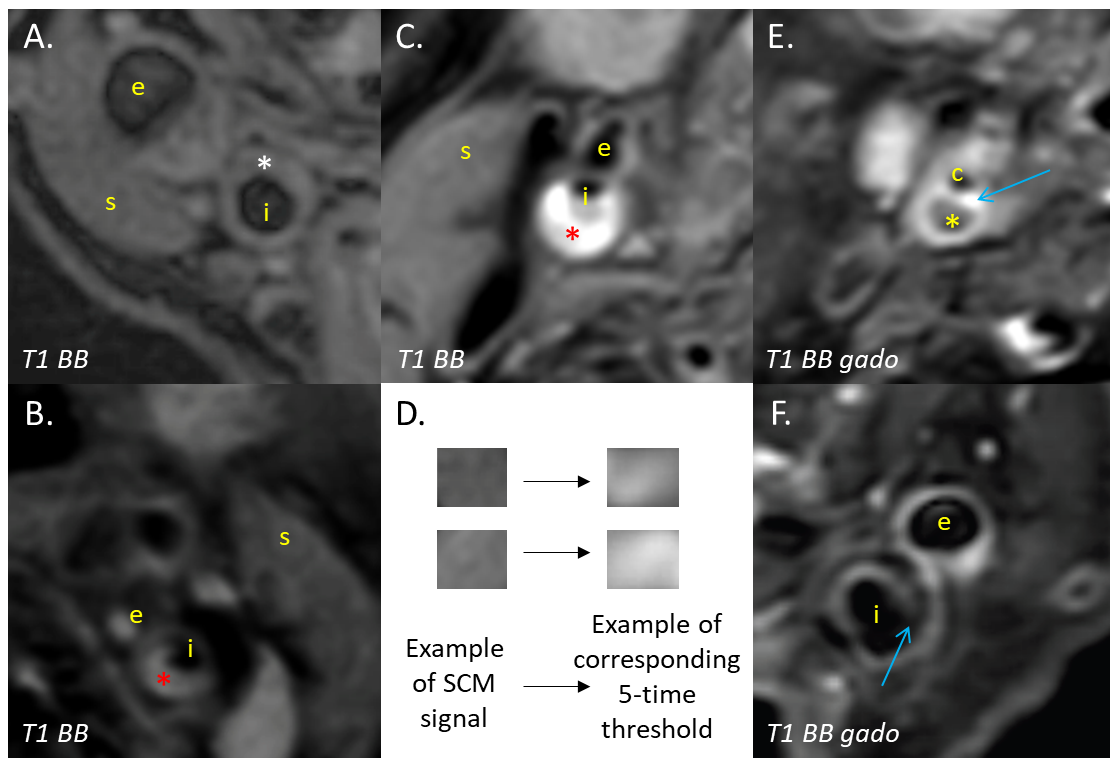


**Supplementary File 1:** *Examples of typical MRI pictures of the carotid atherosclerotic plaque. A. No IPH (scale 0, white *) detected in the T1-weighted sequence; B. IPH signal intensity < 5 times the SCM signal in the T1-weighted sequence (see table 1); C. IPH signal intensity > 5 times the SCM signal in the T1-weighted sequence (see table 1); D. Examples of SCM signal and associated 5-time threshold used to determine IPH signal intensity; E. Evaluation of lipid core and fibrous cap in the T1-weighted sequence with gadolinium contrast injection and F. a ruptured fibrous cap in the T1-weighted sequence with gadolinium contrast injection. Common artery is indicated as c, external carotid as e and internal carotid as i. In panels B and C, IPH (red *) is diagnosed by a visual hyperintensity in the T1-weighted sequence by contrast with the sternocleidomastoid muscle signal (s). In panel E. a thick and regular fibrous cap can be observed in hyperintensity (blue arrow) as well as a large lipid rich core in isointensity (yellow *). In panel F, the blue arrow points to a ruptured fibrous cap.IPH: intraplaque haemorrhage; MRI: magnetic resonance imaging; SCM: sternocleidomastoid muscle.*


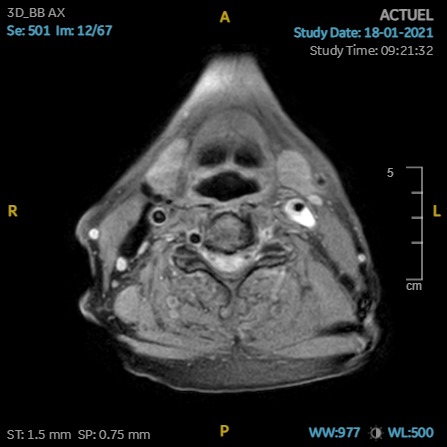

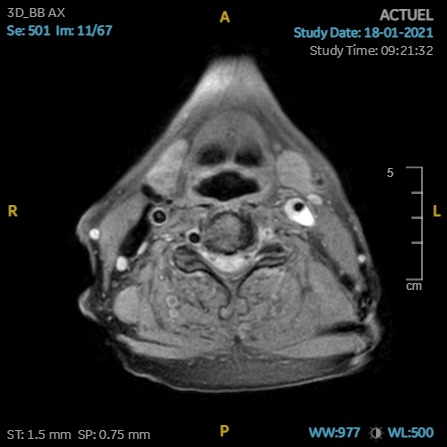

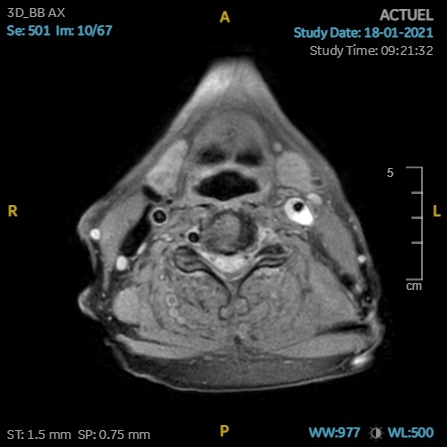

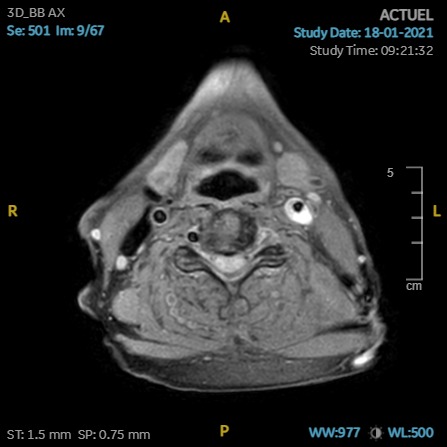

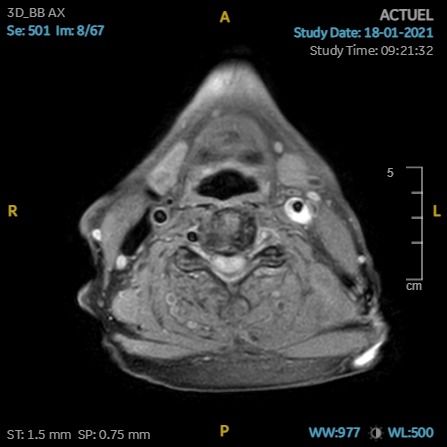

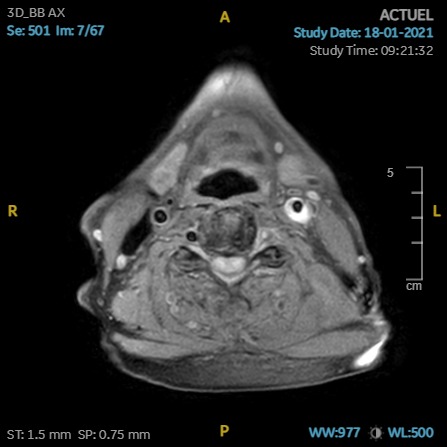

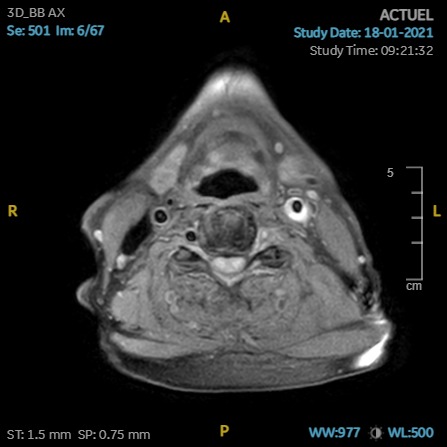

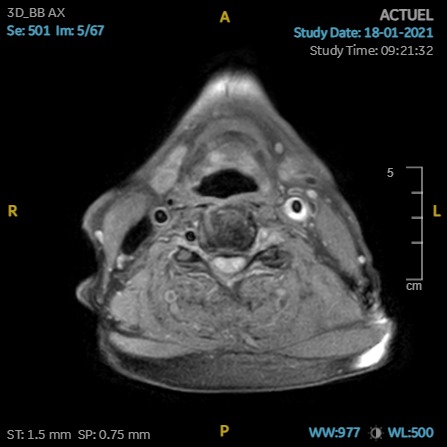

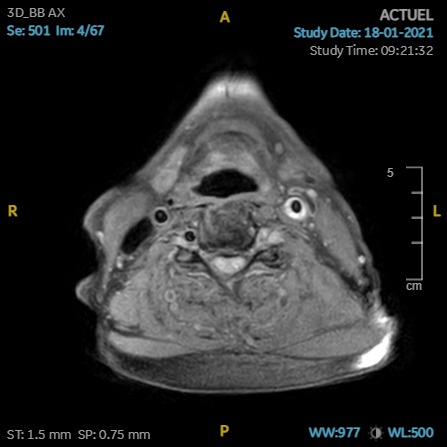

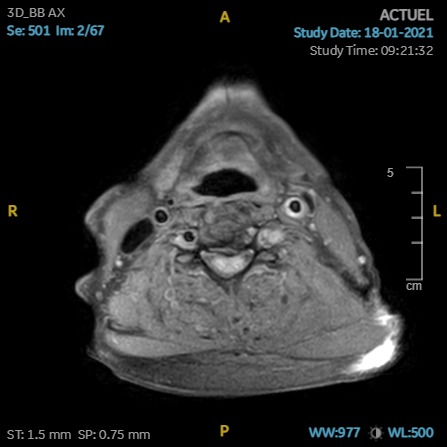

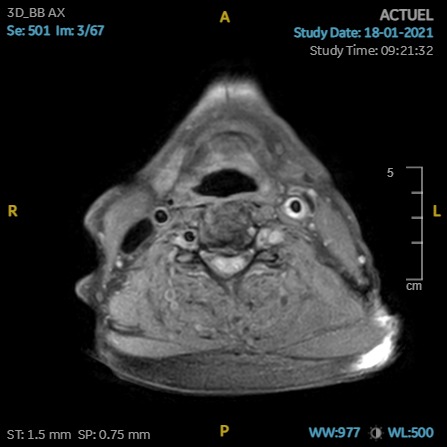

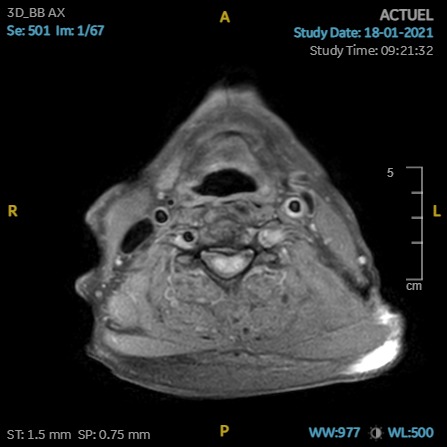


**
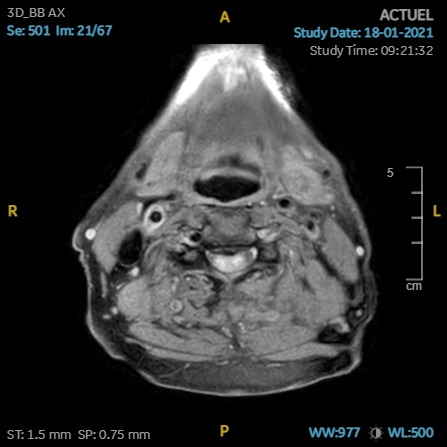

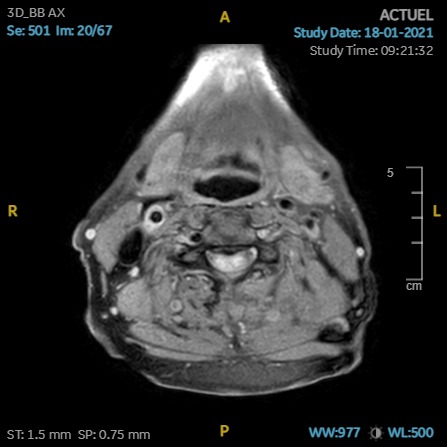

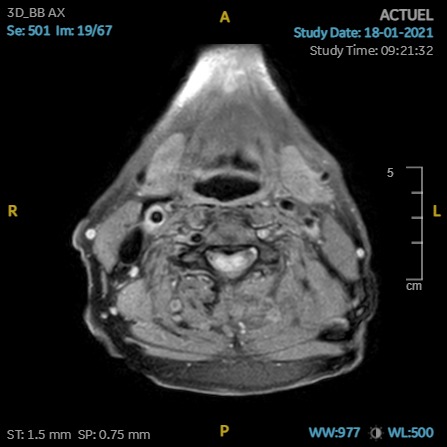

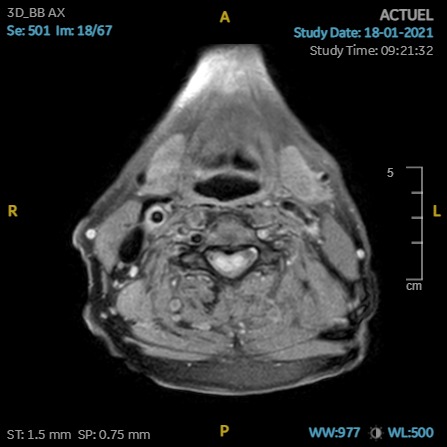

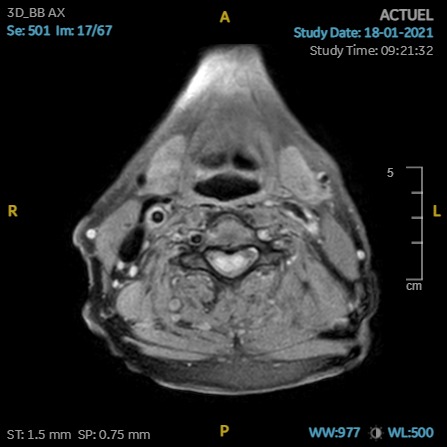

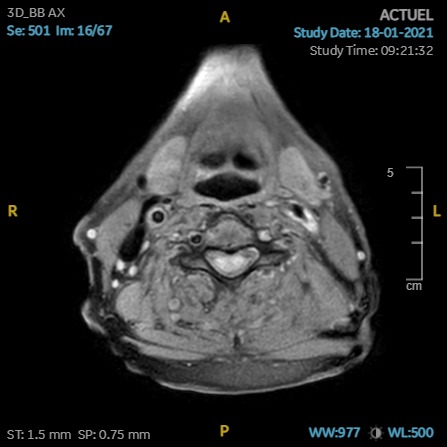

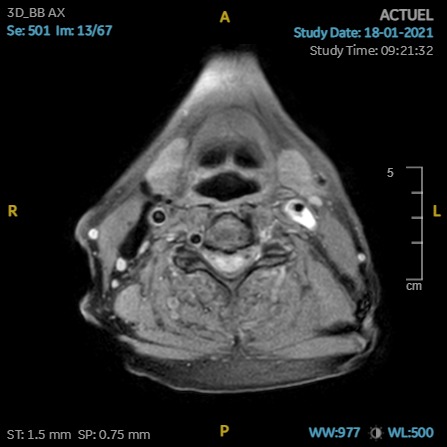

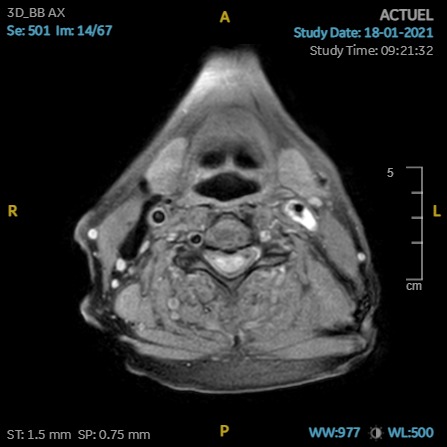

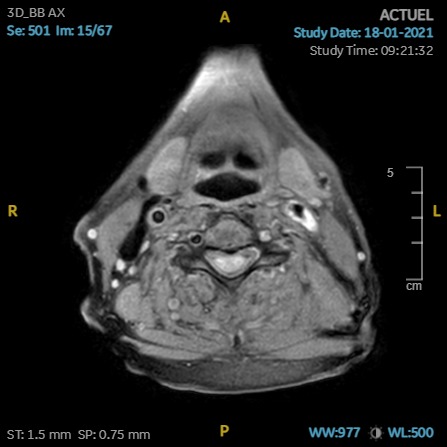
**

**Supplemental file 2:** *Example of a carotid plaque assessed in T1-weighted sequence, on which the IPH can be seen on the right plaque in images 1 to 18.*

**Supplementary File 3: Influence of the group on the post-intervention IPH score according to the adjusted multivariate regression model.**

|  | **β** | **SE** | **Adjusted β** | **Adjusted SE** | **p-value** |
| --- | --- | --- | --- | --- | --- |
| Physical activity arm | -0.32 | 0.15 | -0.43 | 0.20 | **0.04*** |
| Age | 0.02 | 0.01 | 0.26 | 0.11 | **0.03*** |
| Male sex | 0.18 | 0.17 | 0.24 | 0.22 | 0.29 |
| Type 2 diabetes | -0.18 | 0.17 | -0.24 | 0.22 | 0.28 |
| Non-smoker | -0.34 | 0.19 | -0.45 | 0.24 | **0.07** |
| Pre-intervention IPH score | 0.53 | 0.11 | 0.51 | 0.10 | **<0.01*** |

*Clinically relevant relationships in the model are in bold. * p≤0.05 significant p-value into the model 2. Adjusted R² was 0.53 (p<0.01). IPH: intraplaque haemorrhage; SE: standard error.*

**Supplementary file 4:** Mean MRI secondary outcomes in the physical activity and control arms at inclusion and at the end of the trial period.

|  | **Score range** | **PA pre**  (n=23) | **PA post**  (n=23) | **CTRL pre**  (n=25) | **CTRL post**  (n=25) | **p-value**  **Model 1** | **Model 2** |
| --- | --- | --- | --- | --- | --- | --- | --- |
| Calcifications | 0-2 | 1.07 ± 0.61 | 1.15 ± 0.79 | 1.06 ± 0.75 | 0.92 ± 0.66 | 0.41 | 0.50 |
| Lipid core | 0-2 | 0.94 ± 0.71 | 0.96 ± 0.67 | 0.75 ± 0.67 | 0.65 ± 0.62 | 0.28 | 0.10 |
| Fibrous cap | 0-1 | 0.78 ± 0.42 | 0.79 ± 0.42 | 0.58 ± 0.50 | 0.79 ± 0.42 | 0.14 | 0.71 |

CTRL: control arm; MRI: magnetic resonance imaging; PA: physical activity arm; *Pre: measurement at inclusion; Post: measurement at the end of the trial period.*
