# Supplementary material for: Influence of a six-month home-based individualized physical activity intervention on carotid plaque instability measured by magnetic resonance imaging: a randomized controlled clinical trial
Source: eClinicalMedicine. 2025 Apr 22;83:103158. doi: 10.1016/j.eclinm.2025.103158 (PMC12179388; doi:10.1016/j.eclinm.2025.103158)
Supplement: Statistical plan [file mmc2.pdf]

Statistical plan analysis

Initial group homogeneity First of all, we checked for the homogeneity of our groups after randomization. Qualitative data were described by count and percentage and analyzed with Pearson's χ^2 test. Quantitative data were described by mean values and standard deviations and analyzed with Wilcoxon or t-test when appropriate.

All the results were analyzed on an intention-to-treat basis.

Effect of intervention We used linear mixed effects with a by-subject random intercept (*lme* function, *nmle* package)[30] to analyse the effect of the PA intervention on the dependent variables quantifying MRI results, PA levels and metabolic parameters. Visual inspection of the residual plots did reveal obvious deviation from homoscedasticity and/or normality. To investigate the instantaneous effect of GROUP on dependent variables, we entered time (PRE/POST) and group (PA/CTRL) as fixed effects with interaction terms. As post-hoc investigations, we used planned contrasts (emmeans package).

Further analysis of the primary outcome For IPH, in addition to the previous analysis, we performed further analyzes. Multivariate linear regression model assessed the influence of the PA intervention compared to the control group. The multivariate model was controlled for sex, age, diabetes status (yes/no), smoking status (yes/no), IPH score at inclusion, and group. Independent variables were mean-centered. The β coefficient (i.e., the degree of change in the outcome variable for every 1 unit of change in the predictor variable), the adjusted β coefficient (i.e., the degree of change in the outcome variable adjusted to the scale of the explained variable), and the adjusted coefficients R^2 (i.e., percentage of variance explained) were provided. Normality of the residuals of the models were checked. The difference in IPH presence (yes/no) proportions within each group was assessed with a Pearon's χ^2 test. All hypotheses were tested using a statistical significance level of 0.05. Data were analyzed using the R software (version 4.1.2, R Foundation for Statistical Computing, Vienna, Austria).
