# Supplementary material for: Influence of a six-month home-based individualized physical activity intervention on carotid plaque instability measured by magnetic resonance imaging: a randomized controlled clinical trial
Source: eClinicalMedicine. 2025 Apr 22;83:103158. doi: 10.1016/j.eclinm.2025.103158 (PMC12179388; doi:10.1016/j.eclinm.2025.103158)
Supplement: RIPH 1 et 2 PACAPH english [file mmc3.pdf]

Cette trame type de protocole présente les différents chapitres requis pour un projet de recherche impliquant la personne humaine, tels que décrits par les Bonnes Pratiques Cliniques françaises et européennes en vigueur et entrant dans le champ d'application de la loi 2012-300 du 5 mars 2012. Les passages en italique et en bleu sont des conseils rédactionnels ou des passages à adapter aux spécificités de votre projet selon le champ réglementaire de celui-ci : recherche portant sur un médicament, un dispositif médical ou hors produit de santé.
La sélection ou l'adaptation des paragraphes pertinents pour votre étude doit être réalisée en collaboration avec les différents intervenants dans la rédaction du document (investigateurs, méthodologiste-statisticien, pharmacovigilant).

Effets d'une intervention en activité physique individualisée en condition écologique sur les facteurs d'instabilité de la plaque d'athérome carotidienne
Physical Activity intervention and Carotid Atherosclerotic Plaque hemorrhage
PACAPh

Protocol involving human subjects of phase 1 non-drug intervention

Version 1 du 14/03/2019

Promoter: Hospices Civils de Lyon
BP 2251
3 quai des Célestins,
69229 LYON cedex 02

Principal investigator:
Pr Antoine Millon
Service de chirurgie vasculaire
Hôpital Louis Pradel
59 boulevard Pinel
Tél : 04.72.11.11.16
Email : antoinemillon@hotmail.com

Code promoteur : 69HCL19_0345
N°ID-RCB: 2019-A01543-54
Numéro d'enregistrement clinicaltrials.gov : *NCT04053166*
Avis favorable du CPP Sud-Méditerranée le : 26/09/2019
Autorisation de l'ANSM le : 22/07/2019

RESUME

**TITRE**	Effets d'une intervention en activité physique individualisée en condition écologique sur les facteurs d'instabilité de la plaque d'athérome carotidienne.<br>Physical Activity intervention and Carotid Atherosclerotic Plaque Hemorrhage (PACAPh)
**PROMOTEUR**	Hospices Civils de Lyon<br>BP 2251<br>3 quai des Célestins,<br>69229 LYON cedex 02
**INVESTIGATEUR PRINCIPAL**	Pr Antoine Millon<br>Service de chirurgie vasculaire<br>Hôpital Louis Pradel<br>59 boulevard Pinel<br>Tél : 04.72.11.11.16<br>Email : antoinemillon@hotmail.com
**VERSION DU PROTOCOLE**	*1.0 du 14/03/2019*
**JUSTIFICATION / CONTEXT**	Atherosclerosis is a cardiovascular and circulatory disease involving numerous biological processes. In France, circulatory diseases are the leading cause of mortality for women and the second leading cause for men (1). Carotid atherosclerotic plaque instability is the primary cause of ischemic stroke (2) and transient ischemic attack (TIA) due to plaque thromboembolism. According to the World Health Organization (WHO), strokes are the second leading cause of mortality worldwide and the leading cause of long-term disability in industrialized countries (3). Furthermore, the population is aging, and disabling or non-disabling strokes are occurring at increasingly younger ages in men (4).<br><br>Currently, the main criterion for carotid surgical decision-making is the percentage of carotid artery stenosis. However, the plaque composition, reflecting its vulnerability to oxidative and inflammatory processes, appears to be associated with embolic risk. Intraplaque hemorrhage (IPH) is a major anatomical factor in the risk of carotid plaque rupture (5). In a recent cross-sectional study of 90 patients (70 ± 5 years) with asymptomatic carotid plaque (i.e., no history of stroke or TIA), we found that the prevalence of IPH was significantly reduced (69% vs. 31%) in the most physically active and least sedentary patients compared to the most sedentary and least active patients. Indeed, it appears that a threshold of physical activity beyond 900 MET.min/week significantly reduces the frequency of carotid IPH (6).<br><br>IPH can lead to the rupture of the fibrous cap, exposing plaque components, including tissue factor, to the circulation, which is known to activate the coagulation cascade (7), forming a clot that can cause an ischemic stroke (8).<br><br>In a previous study (9), we found an association between low levels of physical activity and increased erythrocyte aggregation, which may play a role in the risk of ischemic stroke.<br><br>In the most physically active and least sedentary subjects, markers of oxidative stress and the percentage of circulating intermediate monocytes (CD14++/CD16+) are reduced compared to the most sedentary and least

	active subjects (Mathilde Mura's Master's study). This should be considered in light of the results of the HOM SWEET HOMe study, which showed that CD14++/CD16+ monocytes were predictive of cardiovascular events in a prospective cohort of subjects eligible for coronary angiography (10).<br><br>Finally, oxidative stress plays an important role in intraplaque hemorrhage and plaque rupture risk (5).<br><br>On the other hand, it has been observed that certain surgical interventions appear to be inadequate in terms of the benefit-risk ratio of carotid endarterectomy, particularly for asymptomatic patients, even with a high percentage of stenosis (11). It is therefore highly likely that more and more patients with severe carotid stenosis will go untreated, for whom it is important to offer appropriate treatment.<br><br>High-resolution magnetic resonance imaging (HR-MRI) of the carotid plaque is considered the most appropriate non-invasive tool for monitoring lesion progression, as it is currently the most accurate imaging modality for detecting IPH (12).<br><br>Finally, the experience of the LIBM shows that a personalized home based 6-month physical activity program using connected activity bracelets for regular goal reassessment in patients with metastatic breast cancer led to excellent adherence throughout the program (>96%) while improving the level of physical activity and physical fitness in these patients (13).
**OBJECTIVES**	• Main objective: The main objective of this study is to evaluate the effect of a 6-month personalized and home based physical activity program on carotid intraplaque hemorrhage (IPH) in patients with untreated carotid atheroma plaque.<br>• Secondary objective(s):<br>To study the effects of a 6-month individualized physical activity intervention in an home based setting on circulating markers associated with carotid atheroma plaque instability in patients.<br>To assess the evolution of physical fitness in patients with untreated carotid atheroma plaque during a 6-month individualized physical activity intervention in an home based setting.<br>To test the feasibility of a 6-month individualized physical activity intervention in an home based setting as a therapeutic approach to address atheroma plaque instability, as an alternative to carotid endarterectomy surgery.
**METHODOLOGY / RESEARCH DESIGN**	Longitudinal Interventional Monocentric Cohort Study Involving Only Minor Risks
**OUTCOMES MEASURE**	• Primary outcome:<br>To measure the variation in IPH during the study, we will assess IPH levels using MRI at baseline (Day 0) and at Month 6. Firstly, the image quality will be evaluated on a scale of 0 to 4, and if the image quality is good (≥3), IPH levels will be determined using a scale of 0 to 3.<br><br>• Secondary outcome(s):<br>- Evaluation of monocyte phenotype using flow cytometry at baseline and Month 6.<br>- Hemato/hemorheological evaluation at baseline and Month 6.

	- Assessment of coagulability at baseline and Month 6.<br>- Evaluation of circulating levels of oxidative stress, antioxidant enzymes, and inflammation through biological assays at baseline and Month 6.<br>- Assessment of circulating cytokine levels using multiplex assays at baseline and Month 6.<br>- Measurement of daily step count for a period of 2 weeks prior to baseline and after Month 6.<br>- Functional tests (6-minute walk test with measurement of gas exchange VO2 and maximal isometric strength test of the quadriceps) at baseline and Month 6.<br>- Quantitative assessment of physical activity using the Global Physical Activity Questionnaire (GPAQ, (14)) at baseline and Month 6.<br>- Assessment of sedentary behavior using a questionnaire (SBQ, (15)) at baseline and Month 6.<br>- Evaluation of quality of life using the EQ-5D-5L questionnaire (16).<br>- Feasibility of the study will be validated if the proportion of patients meeting the recommendations is maintained or increased.<br>- Proportion of patients achieving a predefined level of physical performance (minimum of 6,000 steps per day) at the end of the 6-month program. The number of steps per day will be measured using a connected bracelet worn by each patient in the intervention group throughout the study.<br>- Rate of patients accepting participation in the study, calculated as the ratio of the number of enrolled patients to the number of eligible patients.<br>- Measurement of weight (in kg), height (in cm), waist circumference, and hip circumference (in cm) at baseline and Month 6.
**POPULATION**	Patients with carotid atherosclerotic plaque with more than 50% stenosis, asymptomatic for more than 6 months, and without surgical indication for carotid endarterectomy.
**INCLUSION CRITERIONS**	- Patients with carotid atherosclerotic plaque with ≥ 50% NASCET stenosis.<br>- Followed at the vascular surgery department of the Hospices Civils de Lyon but not surgically treated.<br>- Men or women aged over 18 years.<br>- No contraindication to engaging in physical activity, Performance Status Index (PS) < 2.<br>- Available and willing to participate in the study for its entire duration (6 months).<br>- Capable of understanding, reading, and writing in French.<br>- Affiliated with a social security or similar healthcare system.<br>- Signed and dated an informed consent form.
**NON-INCLUSION CRITERIONS**	- Transient ischemic attack (TIA) or homolateral cerebral infarction within the past 6 months.<br>- History of homolateral carotid surgery or cervical irradiation.<br>- Cancer, heart failure, HIV positivity.<br>- Coronary risk.<br>- Renal insufficiency (creatinine clearance according to Cockcroft < 30 mL/min).<br>- Contraindications and precautions related to Prohance: hypersensitivity to the active ingredient or any of the components of Prohance, renal insufficiency with clearance <30 ml/min/1.73 m², higher likelihood of

	seizures during the examination in patients with epilepsy or brain lesions, pregnancy, breastfeeding.<br>- Contraindication to MRI: ferromagnetic material (including pacemakers, implantable defibrillators, cardiac valve prostheses, cochlear implants, neurostimulators, implanted automated injection devices, intraocular metallic foreign bodies, neurosurgical and vascular clips).<br>- Carotid occlusion.<br>- Homolateral intracranial stenosis.<br>- Risk of pregnancy or confirmed pregnancy based on self-report. Breastfeeding.<br>- Patient under guardianship, curatorship, or legal protection.<br>- Inability to express consent.<br>- Contraindication to engaging in physical activity.<br>- Uncontrolled cardiac or neurological conditions.<br>- Inability to be followed up for medical, social, geographic, or psychological reasons throughout the study duration.
**CRITERIA FOR STUDY WITHDRAWAL**	- Lost to follow-up.<br>- Any cardiovascular event.<br>- Death.
**PROCEDURES**	After the inclusion tests, patients will be randomly assigned to either the control group or the physical activity group. The physical activity group will receive an individualized physical activity intervention, and after the study period, patients will undergo the same tests as at baseline for comparison.
**BENEFIT/RISK RATIO**	The beneficial effects of physical activity (PA) in patients with atherosclerotic plaque have been demonstrated, including a decrease in the incidence of cardiovascular diseases, reduction in carotid IPH occurrence, anti-inflammatory effects, improvement in body composition, quality of life, and physical fitness. However, PA is not commonly recommended for patients with carotid atherosclerotic plaque. Nevertheless, some studies suggest benefits for patients with chronic cardiovascular diseases.<br><br>There is a low risk of traumatic injury associated with the practice of PA. The objectives will be tailored to the patients' abilities. The amount of blood taken for this study is minimal and will not have any impact on the patients' overall health. The blood samples required for the study will not involve any additional travel beyond what is required for the study. We do not anticipate any additional risks or discomfort associated with this study.<br><br>Other potential risks that patients may be exposed to are inherent to standard treatments for the disease, and these have been explained by the physician responsible for the patient's care.
**NUMBER OF SUBJECTS**	Estimated number of individuals: 88 subjects, with 44 in the intervention group and 44 in the control group.
**STUDY DURATION**	Inclusion duration: 18 months<br>Duration of participation for each patient: 1 and 2 hours, respectively, for each appointment (2 appointments).<br>For patients in the intervention group: 6 months.

	Total duration of the study: 2 years.
RESEARCH LOCATION	Hôpital Louis Pradel des Hospices Civils de Lyon, Lyon, France
EPECTED OUTCOMES	This study will help determine the effects of an individualized physical activity intervention in an home based setting for non-operated patients with carotid atheromatous plaque and stenosis ≥ 50%. This will provide evidence to propose physical activity as an alternative to surgery for these patients.

LISTE DES ABREVIATIONS

**15-LO**	15-Lipoxygenase
**ACABII**	Athérosclérose Carotidienne : Biomarqueurs d'Imagerie Innovants
**ACAS**	Asymptomatic Carotid Atherosclerosis Study
**ACST**	Asymptomatic Carotid Atherosclerosis Trial
**AIT**	Accident Ischémique Transitoire
**AMM**	Autorisation de Mise sur le Marché
**ANSM**	Agence Nationale de Sécurité des Médicaments et des produits de santé
**AP**	Activité Physique
**ARC**	Attaché de Recherche Clinique
**AVC**	Accident Vasculaire Cérébral
**AVCi**	Accident Vasculaire Cérébral Ischémique
**BPC**	Bonnes Pratiques Cliniques
**CCTIRS**	Comité Consultatif sur le Traitement de l'Information en matière de Recherche dans le domaine de la Santé
**CD**	Cluster de Différenciation
**cIMT**	Carotid Intima Media Thickness (Epaisseur de l'intima et de la média)
**CMV**	Contraction Maximale Volontaire
**CNIL**	Commission Nationale Informatique et Liberté
**CPP**	Comité de Protection des Personnes
**CRP**	C-Reactive Protein (Protéine C-Réactive)
**CRPV**	Centre Régional de la Pharmacovigilance
**CRF**	Case Report Form (cahier d'observation)
**CSP**	Code de la Santé Publique
**DIRC**	Direction Inter-régionale de Recherche Clinique
**DRCI**	Délégation à la Recherche Clinique et à l'Innovation
**ECST**	European Carotid Surgery Trial
**EI**	Evénement Indésirable
**EIG**	Evénement Indésirable Grave
**eNOS**	Edothelial Nitric Oxyde Synthase (Synthetase de l'Oxyde Nitrique endothéliale)
**GSH**	Glutathion Réduit
**HCL**	Hospices Civils de Lyon
**Hb**	Hémoglobine
**HDL**	High Density Lipoprotein (lipoprotéine de haute densité)
**HOM SWEET HOMe**	Heterogeneity Of Monocytes in Subjects Who undergo ElectivE coronary angiography – The HOMburg evaluation
**HTA**	Hypertension Artérielle
**ICH**	International Conference on Harmonisation
**IE**	Index d'Elongation
**IFN-γ**	Interféron-γ
**iNOS**	Inductible Nitric Oxyde Synthase (Synthetase de l'Oxyde Nitrique inductible)
**IPH**	IntraPlaque Hemorrhage (Hémorragie Intra-Plaque)
**IRM**	Imagerie par Resonance Magnétique

**IRM HR**	Imagerie par Résonnance Magnétique Haute Résolution
**LDL**	Lox Density Lipoprotein (Lipoprotéine de basse densité)
**MET.min/sem**	Metabolic Equivalent of Task per minute per week (Equivalent Métabolique de la Tâche par minute par semaine)
**mmHG**	Millimètres de mercure
**$mPa.s^{-1}$**	Millipascal par seconde
**MR**	Méthodologie de Référence
**NASCET**	North American Symptomatic Carotid Endarterectomy Trial
**NO**	Nitric Oxyde (monoxide d'azote)
**$O_2^{\circ-}$**	Anion Superoxyde
**Pa**	Pascal
**PUI**	Pharmacie à Usage Intérieur
**RCP**	Résumé des Caractéristiques du Produit
**ROS**	Reactive Oxygen Species (Espèces Réactives de l'Oxygène)
**RPE**	Rate of Perceived Exertion (échelle de perception de l'effort)
**SOD**	Super Oxyde Dismutase
**SUSAR**	Suspected Unexpected Serious Adverse Reaction
**TEC**	Technicien d'Etude Clinique
**TM6**	Test de Marche de 6 minutes
**VO_2max**	Volume d'Oxygène maximal
**VRB**	Volontaires pour les Recherches Biomédicales
**WHO**	World Health Organisation (Organisation Mondiale de la Santé)

SOMMAIRE

1 INFORMATIONS GENERALES 11
1.1. Titre 11
1.2. Identifiants du projet et historique des mises à jour .... **Erreur ! Signet non défini.**
1.3. Promoteur 11
1.4. Investigateurs 12
*1.4.1. Investigateur principal ** 12
1.4.2. Associated investigators 12
Monocentric study 12
1.5. Associated scientists 12
1.6. Methodologist – Biostatistician 12
1.7. Pharmacist 13
1.8. Comities 13
1.8.1. Scientific comities 13
2 SCIENTIFIC JUSTIFICATION 13
2.1 Rational 13
2.1.1 Atherosclerosis epidemiology 13
2.1.2 Physical activity and atherogenesis 13
2.1.3 Physical activity and carotid plaque instablity 14
2.1.4 Necessity of follow-up for patients 15
2.2 Research hypothesis 15
2.3 Justification of methodology 15
2.3.1 Tool of plaque instability follow-up 16
2.3.2 Interest of home based Physical activity 16
2.4 Population 16
2.5 Benefits / risks ratio 16
Benefits: 16
Risks : 17
2.6 Expected outcomes 17
3 Research objective 18
3.1 Main objective 18
3.2 Secondary objectives 18
4. RESEARCH CONCEPTION 18
4.1 Type of study 18
3.2 Randomization 18
3.3 Endpoint 18
3.3.1 Primary outcomes 18
3.3.2 Secondary outcomes 19
4 ELIGIBILITY CRITERIONS 19
4.1 Pre-inclusion criterions 19
4.2 Inclusion criterions 19
4.1 Non-inclusion criterions 19
4.2 Premature 20
4.3 Recruitment modalities and feasibility 20
5 EXPERIMENTAL STRATGIES 20
5.1 Study strategy 20
6 GENERAL ORGANIZATION 21
6.1 Study calendar 21
6.2 General figure and recapitulative table 21
6.3 Study design 23
6.3.1 Screening – Pre-inclusion 23

6.3.2 Inclusion visit / Randomization 24
6.3.3 Phone call follow up 27
6.3.4 End of the study visit 28
6.4 Rules of temporary or definitive study termination 29
6.5 Collection of biological samples 29
7 SECURITY EVALUATION 30
7.1 Definitions 30
7.1.1 Adverse event 30
7.1.2 Severe adverse event (EIG) 30
7.1.3 Adverse effect (EI) 31
7.2 Investigator responsibility 31
7.2.1 Procedures for the detection and collection of adverse events. 31
7.2.2 EIG notification 31
7.2.3 Serious Adverse Events Not Requiring Immediate Notification to the Promotor 32
7.2.4 Adverse event of particular interest 32
7.2.5 Evaluation of causality 32
7.2.6 *Period for immediate notification of SAEs to the promotor by the investigator and procedures for monitoring serious adverse events.* 32
7.3 Responsibility of the promotor 32
7.3.1 Declaration to authorities 32
7.3.2 Description of research-related adverse effects (safety reference for the promotor's assessment of expected/unexpected nature) 33
7.4 Independent surveillance comity 33
8 STATISTICS 34
8.1 Population needed 34
8.2 Statistical method Ananlysis 34
8.3 Missing data 35
8.4 Management of modifications made to the analysis plan 35
8.5 Biostatistician 35
9 RESEARCH SURVEILLANCE 36
9.1 Access to data 36
9.2 Source documents 36
9.3 Data confidentiality 36
10 CONTROL AND QUALITY INSURANCE 37

1 INFORMATIONS GENERALES

1.1. Title

Effets d'une intervention en activité physique individualisée en condition écologique sur les facteurs d'instabilité de la plaque d'athérome carotidienne (PACAPh)

1.2. Project Identifiers and Update History

Code promoteur : 69HCL19_0345
N°ID-RCB: 2019-A01543-54
Numéro d'enregistrement clinicaltrials.gov : *NCT04053166*
Avis favorable du CPP Sud-Méditerranée le : 26/09/2019
Autorisation de l'ANSM le : 22/07/2019

Historique des versions		
**Version**	**Date**	**Motif de la mise à jour**
	XX/XX/XXXX	Rédaction initiale

1.3. Promotor

- *IdentityF :*

Hospices Civils de Lyon
BP 2251
3 Quai des Célestins
69229 LYON Cedex 02

- *Signature du protocole au nom du Promoteur :*

Muriel MALBEZIN, Directeur de la Recherche Clinique et de l'Innovation
Hospices Civils de Lyon, Direction de la Recherche Clinique et de l'Innovation, Siège Administratif, BP 2251, 3 Quai des Célestins, 69229 LYON Cedex 02
Tél : 04 72 40 68 52, Fax : 04 72 40 68 69

- *Responsable de la recherche au niveau du Promoteur :*

Valérie PLATTNER, médecin référent
Hospices Civils de Lyon, Direction de la Recherche Clinique et de l'Innovation, Siège Administratif, BP 2251, 3 Quai des Célestins, 69229 LYON Cedex 02
Tél : 04 72 40 68 40, Fax : 04 72 11 51 90

- *Responsable de la vigilance des essais au niveau du Promoteur :*

Marina NGUON, pharmacien référent
Hospices Civils de Lyon, Direction de la Recherche Clinique et de l'Innovation, Siège Administratif, BP 2251, 3 Quai des Célestins, 69229 LYON Cedex 02
Tél : 04 72 40 68 26, Fax : 04 72 11 51 90

1.4. Investigators

Pr Antoine Millon
Service de chirurgie vasculaire
Hôpital Louis Pradel
59 boulevard Pinel 69500 Bron
Tél : 04.72.11.11.16
Email : antoinemillon@hotmail.com

Attention, vérifier la durée de validité de l'ALR si étude du 1° :

- 7 ans
- 3 ans si volontaire sain + 1ere administration à l'homme

1.4.1. Principal investigator *

Pr Antoine Millon
Service de chirurgie vasculaire
Hôpital Louis Pradel
59 boulevard Pinel 69500 Bron
Tél : 04.72.11.11.16
Email : antoinemillon@hotmail.com

1.4.2. Associated investigators

Monocentric study

1.5. Associated scientists

Mathilde MURA
Laboratoire interuniversitaire de la biologie et de la motricité
8 avenue Rockefeller
Email : mathilde.mura@univ-lyon1.fr

Vincent Pialoux
Laboratoire interuniversitaire de la biologie et de la motricité
8 avenue Rockefeller
Email : vincent.pialoux@univ-lyon1.fr

Camille Faes
Laboratoire interuniversitaire de la biologie et de la motricité
8 avenue Rockefeller
Email : camille.faes@univ-lyon1.fr

1.6. Methodologist – Biostatistician

-

Vincent Pialoux
Laboratoire interuniversitaire de la biologie et de la motricité
8 avenue Rockefeller
Email: vincent.pialoux@univ-lyon1.fr

1.7. Pharmacist

Non-applicable

1.8. Comities

1.8.1. Scientific comities

To ensure regular monitoring of the study progress, a steering committee has been established. This committee will address any logistical and scientific issues that may arise during the study. The committee, which will meet on a quarterly basis, is composed of the following individuals or their representatives:

- Pr Antoine Millon, investigateur principal
- Pr Vincent Pialoux, physiologiste, Université Claude Bernard Lyon 1

2 SCIENTIFIC JUSTIFICATION

2.1 Rational

2.1.1 Atherosclerosis epidemiology

Atherosclerosis is a complex vascular inflammatory disease characterized by lipid deposition and macrophage infiltration in the arterial walls of large-caliber bifurcations, forming an atherosclerotic plaque. In France, circulatory diseases are the leading cause of mortality for women and the second leading cause for men (1). Rupture of an atherosclerotic plaque, regardless of its vascular location, is the main cause of mortality worldwide (3). The atherosclerotic plaque remains silent until its instability leads to major complications, which can impact the patient's functional and even vital prognosis. Instability of the carotid atherosclerotic plaque can lead to the formation of a thrombus on its surface, which, when detached, can obstruct a smaller downstream vessel, resulting in ischemic stroke. Stroke is one of the leading causes of mortality worldwide and the primary cause of non-traumatic disability. 80% of strokes are ischemic strokes, and approximately 80% of them are caused by carotid plaque rupture (2). Hence the importance of limiting the instability of the carotid atherosclerotic plaque.

Carotid endarterectomy surgery involves the removal of the carotid atherosclerotic plaque. Its benefits have been extensively demonstrated in large cohort studies (17,18) for patients who have already experienced an ischemic stroke. However, for patients who have never had an ischemic stroke, its benefits are controversial (19) as the surgical procedure itself increases the risk of ischemic stroke during the postoperative period of 1 to 2 months. When surgical treatment is not feasible, medical treatment targeting risk factors such as smoking, dyslipidemia, hypertension, and type 2 diabetes is implemented, but nothing is proposed to address their sedentary behavior. Furthermore, there is no real monitoring of the carotid atherosclerotic plaque for these patients. In light of the decline in surgical procedures for asymptomatic patients with a high-risk carotid atherosclerotic plaque (20), an improvement in their management should be offered. Physical activity thus appears as a potential therapeutic target. It helps combat the sedentary lifestyle of these patients, and it has been shown to reduce the incidence of ischemic strokes (21).

2.1.2 Physical activity and atherogenesis

Chronic physical activity (PA) has numerous beneficial effects as it reduces the incidence of vascular diseases in the carotid arteries (20).

The effects of PA on risk factors for carotid atherosclerotic plaque formation have been widely described in the literature, but its effects on plaque instability remain poorly studied.

PA training prevents the development of dyslipidemia (22) and reduces plasma low-density lipoprotein (LDL) levels in healthy individuals (23), hypercholesterolemic men (24), and obese women (25). LDLs are involved in the pathogenesis of atherosclerotic plaque through their accumulation in the arterial wall of pro-atherogenic areas. High LDL concentration can affect the ability of high-density lipoprotein (HDL) to eliminate cholesterol. An increase in plasma concentration has been observed in individuals trained in PA (26). In the vessel wall, LDLs can be oxidized by reactive oxygen species (ROS) such as myeloperoxidases, 15-LO, or iNOS. Reducing LDL plasma concentration could be achieved through ROS reduction. In humans, a study showed that exercise increases the resistance of plasma LDLs to oxidation and decreases the plasma concentration of already oxidized LDLs (27). Reduced glutathione (GSH) plays a role in managing lipid uptake in advanced lesions (28). In vitro, antioxidant supplementation in macrophages reduced their uptake of oxidized LDLs (29). Furthermore, moderate-intensity exercise has been shown to reduce the inhibitory effects of oxidized LDLs on the bioavailability of superoxide dismutase (SOD) and GSH.

Numerous studies have demonstrated that PA induces improved endothelial function in healthy individuals as well as in individuals with endothelial dysfunction, such as those with coronary artery disease, chronic heart failure, and type 2 diabetes (30). Physical exercise mechanically stimulates the endothelium through the shear stress it induces, leading to the production of nitric oxide (NO) by endothelial nitric oxide synthase (eNOS) and improving vasodilation (31). Physical exercise also helps prevent the degradation of NO by reducing oxidative stress. PA training reduces oxidative activity induced by NADPH oxidase (32), IFN-γ, and superoxide anion (O2°-) (33). The improvement in PA-induced vasodilation is often accompanied by upregulation of SOD (34). The increased SOD activity induced by shear stress could be responsible for the decreased inhibitory activity of O2°- on NO (35).

The recruitment of circulating monocytes and their differentiation into macrophages in the vessel wall is an important step in atherosclerotic plaque formation. Adhesion of blood cells to the endothelium is limited in healthy individuals. In conditions of low-grade inflammation in atherosclerosis, the expression of adhesion molecules is increased, leading to increased diapedesis of circulating monocytes (36). In trained individuals, the expression of these adhesion molecules is decreased (37). Macrophages have the ability to change phenotype in response to environmental changes. It is therefore highly likely that systemic changes induced by PA could have an effect on plaque macrophage phenotype. It has been shown that low-intensity exercise increases the expression of anti-inflammatory macrophage markers while decreasing pro-inflammatory markers (38).

Finally, PA, through an increase in maximal oxygen volume (VO2max), decreases the intima and media thickness of the carotid artery (cIMT) (39). cIMT is a risk factor for atherogenesis as it induces local hypoxia. PA plays a major role in the formation of carotid atherosclerotic plaque, but its effects on plaque instability have been poorly studied.

2.1.3 Physical activity and carotid plaque instability

Intraplaque hemorrhage (IPH) is a major anatomical factor for the risk of carotid plaque rupture (8) and is predictive of ischemic events (5) such as strokes. IPH results from the leakage of immature neovessels (40), facilitated by a hypoxic and pro-inflammatory environment. Furthermore, by activating plaque macrophages, IPH attracts circulating monocytes, which enter the vessel wall through diapedesis and differentiate into macrophages, thereby maintaining the pro-inflammatory environment (41). Our team recently demonstrated through a cross-sectional study of 90 patients (70 ± 5 years) with asymptomatic carotid plaque (i.e., no history of stroke or transient ischemic attack) that the prevalence of IPH was significantly reduced (69% vs. 31%) in the most active and least sedentary patients compared to the least active and most sedentary patients. It was found that a

threshold of physical activity beyond 900 MET.min/week significantly reduces the frequency of carotid IPH (6). Finally, oxidative stress plays an important role in intraplaque hemorrhage and plaque rupture risk (5).

IPH can lead to the rupture of the fibrous cap, exposing plaque components, including tissue factor, to the circulation, known to activate the coagulation cascade (7), forming a clot that can cause an ischemic stroke (8).

In a previous study (6), we found an association between low levels of physical activity and increased erythrocyte aggregation, which may play a role in the risk of ischemic stroke.
In the most active and least sedentary subjects, our preliminary results show that markers of oxidative stress and the percentage of intermediate circulating monocytes (CD14++/CD16+) are reduced compared to the least active and most sedentary subjects. This is in line with a study by Van Craenenbroek (42), which shows a decrease in intermediate monocytes (CD14++/CD16+) in response to acute exercise, although the stimulus (acute PA vs. chronic low-intensity PA) and the experimental design (interventional study vs. cross-sectional study) are different. This should be considered in light of the results of the HOM SWEET HOMe study, which showed that CD14++/CD16+ monocytes were predictive of cardiovascular events in a prospective cohort of subjects eligible for coronary angiography (10). In addition to the phenotypic changes of plaque macrophages towards a more pro-inflammatory phenotype (38), the quantity of macrophages accumulated in the plaque can affect the severity of atherosclerotic plaque lesions.

2.1.4 Necessity of follow-up for patients

Carotid endarterectomy surgery allows for the removal of carotid plaque. Its benefits have been extensively demonstrated in large cohort studies for patients with symptomatic ischemic strokes (AVCi). However, it has been observed that for patients who have never had an ischemic stroke, the risk-benefit ratio of surgery is unfavorable, even with a high degree of carotid stenosis (11,19). In fact, the surgery itself increases the risk of ischemic stroke during the postoperative period of one to two months. Moreover, 95% of strokes may still occur despite the surgery (43). The ACAS cohort study on asymptomatic stroke patients showed no beneficial effects of surgery at 4 years (11). It is, therefore, highly likely that an increasing number of patients with significant carotid stenosis will opt not to undergo surgery. For these patients, it is important to be able to propose an appropriate treatment.

Thus, the aim of our study will be to evaluate the effects of an individualized physical activity intervention in an home based setting on the factors of instability of non-operated carotid atherosclerotic plaque, based on the analysis of intraplaque hemorrhage (IPH) levels measured by MRI. The changes in IPH intensity levels before and after the intervention will allow us to characterize the effects of the intervention. Our hypothesis is that this physical activity program should decrease the factors of instability of carotid atherosclerotic plaque.

2.2 Research hypothesis

Our hypothesis is that this individualized physical activity intervention in an home based setting decreases the levels of IPH, the proportion of pro-inflammatory monocytes, inflammatory and oxidative stress levels, increases antioxidant levels, and improves the physical capacity of patients (with carotid atherosclerotic plaque with more than 50% stenosis, asymptomatic for more than 6 months, without surgical indication for endarterectomy) in the intervention group compared to the patients in the control group.

2.3 Justification of methodology

2.3.1 Tool of plaque instability follow-up

IPH has been demonstrated to be the primary risk factor for plaque rupture (5). The gold standard tool for detecting IPH remains histology, but this technique requires surgical removal of the plaque. It has been shown in the literature that magnetic resonance imaging (MRI) of the carotid plaque demonstrates a good correlation with histology in detecting IPH in vivo (12, 44, 45). Carotid plaque MRI is considered the most appropriate non-invasive tool for monitoring lesion progression as it is currently the most accurate imaging modality for detecting IPH (12).

2.3.2 Interest of home-based Physical activity

These patients are not undergoing surgery, so an alternative therapeutic approach needs to be proposed. According to the literature, physical activity appears to be a promising candidate as it decreases levels of reactive oxygen species (MDA) (6), LDL (46), pro-inflammatory cytokines (47), CRP (47, 48), fibrinogen (46), adhesion molecules (47), and circulating leukocytes (46). A meta-analysis conducted by Lee shows that physical activity reduces the prevalence and mortality of both ischemic and hemorrhagic strokes by 65% in cross-sectional studies and 25% in longitudinal studies.

This type of protocol does not require people to travel to specialized centers (avoiding time and travel constraints), which increases feasibility and patient recruitment rates. Moreover, the adherence rate to physical activity for this type of program is higher than that of a conventional physical activity program (49). Our goal is the long-term sustainability of physical activity, which is why we will allow patients to keep the wearable device at the end of the intervention.

According to the World Health Organization, physical activity above 150 minutes of moderate-intensity physical activity (or 600 Metabolic Equivalent of Task-minutes/week: MET-min/week) reduces cardiovascular mortality in older individuals (WHO 2010). A threshold of 1600 MET-min/week would reduce stroke-related mortality by 21% (50). For inactive older adults, physical activity increases life expectancy, physical function, quality of life, and independence (51). It has recently been demonstrated that the duration of physical activity, rather than its intensity, is inversely associated with cardiovascular risk (52). Therefore, encouraging moderate physical activity based on daily step counts appears relevant for these patients.

In the cross-sectional study we conducted on the same type of patients, the analysis of physical activity questionnaires revealed that a large majority (over 90%) of the physical activity performed by the patients was moderate in intensity and primarily related to walking (e.g., leisurely walks, housework, gardening), which aligns with the physical activity intervention in this protocol. By gradually increasing the goals, excellent adherence to the physical activity intervention can be expected, as demonstrated by the compliance rates in our team's study on metastatic breast cancer (13). Moreover, this interventional study showed effectiveness on physical fitness markers such as TM6 or maximal isometric quadriceps strength, as well as an effect on biological markers including oxidative stress.

2.4 Population

This study will include adult patients with carotid atherosclerotic plaque with more than 50% stenosis, who have been asymptomatic for more than 6 months and do not have a surgical indication for endarterectomy.

The criteria for selecting participants are detailed in paragraph 5.

2.5 Benefits / risks ratio

Benefits:

The beneficial effects of physical activity (PA) in patients with atherosclerotic plaque have been demonstrated, including a reduction in the incidence of cardiovascular diseases (20), a decrease in the occurrence of carotid IPH (6), anti-inflammatory effects (53), improvement in body composition, quality of life, and physical fitness.

However, physical activity is not typically recommended for patients with carotid atherosclerotic plaque. Nevertheless, some studies suggest potential benefits for patients with chronic cardiovascular diseases (51).

Risks:

There is a low risk of traumatic injury associated with the practice of PA. The objectives will be tailored to the patients' abilities.

The amount of blood collected for this study is minimal and will not have any consequences on the general health of the patients. The blood samples required for the study will not require any additional travel beyond the scheduled visits for the study.

Two additional visits related to this study will be necessary (at baseline and at 6 months). The completion of study questionnaires will take place at the participating center during the scheduled study consultations.

We do not anticipate any additional risks or discomfort associated with this study. Any other potential risks the patient may be exposed to are inherent to standard treatments for the disease and have been explained by the physician managing the patient.

We believe that this study presents a favorable benefit-to-risk ratio as the expected benefits outweigh the foreseeable and known risks for individuals participating in the research.

2.6 Expected outcomes

This study will allow:

1) Characterizing the effects of a 6-month individualized PA intervention in a home-based setting on IPH levels detected by MRI in patients with non-operated carotid atherosclerotic plaques.

2) Characterizing the circulating effects of a 6-month individualized PA intervention in a home-based setting on monocyte phenotype, oxidative stress, antioxidant, and inflammatory levels in patients with non-operated carotid atherosclerotic plaques.

3) Characterizing the muscle function of a 6-month individualized PA intervention in a home-based setting through questionnaires and functional tests in patients with non-operated carotid atherosclerotic plaques.

4) In the long term, proposing PA as an alternative to surgery.

Ultimately, this study will help define the mechanisms involved in the stabilization of non-operated carotid atherosclerotic plaques through physical activity.

3 Research objective

3.1 Main objective

The main goal of this study is to evaluate the effect of a home-based physical activity (PA) intervention of 6-months on carotid intraplaque hemorrhage (IPH), in patients with non-operated carotid stenosis.

3.2 Secondary objectives

1) Evaluate the effect of a home-based PA intervention of 6-months on circulating biomarkers linked to carotid plaque instability.

2) Evaluate the effect of a home-based PA intervention of 6-months on physical capacities.

3) Test the feasibility of a home-based PA intervention of 6-months, for therapeutic use of physical activity on carotid plaque instability as an alternative to carotid endarterectomy.

4) Evaluate the effect of a home-based PA intervention of 6-months on anthropometric parameters.

4. RESEARCH CONCEPTION

4.1 Type of study

Monocentric interventional randomized clinical study.

Research of type 1 according to the article L1121-1 of the *loi Jardé 2012-300* of the *5 mars* 2012 of the French health law

This monocentric study will involve the vascular and endovascular unit and the radiology unit of the Louis Pradel hospital from the Hospices Civils de Lyon (Lyon)

The study include two visits. The participants will be randomized either in the control or in the PA group aftr the inclusion visit.

3.2 Randomization

The physician who included the patient will randomize the patient after the inclusion meeting. The randomization will be 1:1. Randomization will be checked by the biostatistician in the electronic CRF.

3.3 Endpoint

3.3.1 Primary outcomes

The primary outcome is the decrease in IPH. The decrease in IPH will be determined as the reduced levels of IPH intensity measured by MRI at the end of the intervention, in comparison with

the inclusion, for the patients of the PA group in comparison with the control group. After the scoring of the image's quality, only the better images will be further processed. Intensity of IP will be scored from 0 to 3 with 0: absence, 1: low presence, 2, moderated presence and 3: important presence of IPH.

Same principle will be applied to calcifications and volume of lipidic core. Fibrous cap will be evaluated as intact or ruptured.

3.3.2 Secondary outcomes

To go further, we will also circulating biomarkers linked to carotid plaque instability: monocytes phenotype ($CD14^{++}CD16^{-}$ classical monocytes, $CD14^{++}CD16^{+}$ intermediate monocytes and $CD14^{+}$ $CD16^{++}$ non-classical monocytes), blood rheology markers, (red blood cells aggregation and, elongation index), coagulation markers (clot formation time, elasticity and firmness), oxidative stress markers (MDA, AOPP), antioxidants (SOD, Catalase, GPX), pro-inflammatory (IL-1B), as well as physical capacities markers (anthropometric data, 6-minutes' walk test with VO_2, isometric quadriceps strength test, questionnaires of sedentary behavior, PA, and quality of life).

4 ELIGIBILITY CRITERIONS

4.1 Pre-inclusion criterions

Non-endarterectomized patient that already underwent the study *Athérosclérose Carotidienne: Biomarqueurs d'Imagerie Innovants* (ACABII, Clinical trial: NCT02748941) or that already underwent a high-resolution MRI of the carotid plaque in the last 4 months. The primary objective of this transversal study is to determine the combination of criterions that are the most performant to predict carotid atherosclerotic plaque instability. It also will compare MRI to the anatomopathological results in operated patients and lead a hemodynamic analysis of the environment from the MRI

4.2 Inclusion criterions

- Patient with a carotid stenosis ≥ 50% NASCET,
- Followed at the vascular surgery of the Louis Pradel hospital from the Hospices Civils de Lyon, without carotid surgery,
- Men and women older than 18 years old,
- No contraindications to PA, performance scale PS <2
- Disponible and voluntary to invest himself during the 6-month of the study
- Able to understand, read and write French
- Affiliated to the French health care system or assimilated,
- Dated and signed informed consent.

4.1 Non-inclusion criterions

- TIA or homolateral cerebral infarct < 6 months;
- History of homolateral or cervical irradiation;
- Cancer, heart failure, seropositivity;
- Renal failure (Cockcroft creatinine clearance < 30 mL/min);
- Contraindication and precaution to use of Prohance: hyper-sensibility to one of the constituents de of Prohance, renal failure (Cockcroft creatinine clearance < 30 mL/min), susceptibility to convulsions during the exam higher in epileptic patients, or patients suffering of brain lesions, pregnancy or breastfeeding;

- MRI contraindication: ferromagnetic materials (pacemaker, implantable defibrillators, valvular cardiac prosthetics, cochlear implants, neurostimulators, implantable automated injection material, intra ocular metallic debris, neurochirurgical or vascular clips);
- Carotid occlusion;
- Coronary risk;
- Homolateral intracranial stenosis;
- Pregnancy or risk of pregnancy. breastfeeding;
- Guardianship, curatorship, maintenance of justice;
- Incapacity to express consent;
- Non-controlled cardiologic or neurologic diseases;
- Impossibility of follow-up due to medical, social, geographic or psychologic reason during the whole study.

4.2 Premature

Rules of premature permanent end of the study are:

- Withdrawal of the consent;
- End of the study by the promotor or investigator;
- Loss of sight;
- Death.

Withdrawal of the consent is immediate and will be notified by the principal investigator on the observation notebook.

4.3 Recruitment modalities and feasibility

The active file of patients diagnosed with a carotid atheromatous plaque with a stenosis > 50% but non-operated is 150 patient per year at the *Hospices Civils de Lyon*.

The study *Athérosclérose Carotidienne: Biomarqueurs d'Imagerie Innovants* (ACABII) has already been proposed to all these patients. At the end of this protocol, the PACAPh study will be proposed to all the patients for whom no surgical option was needed. Patients will be identified by the clinic research associate of the unit of vascular surgery of the hospital Louis Pradel. In order to estimate the number of patients potentially eligible and counting on 50% rejection, it is planned to recruit 4.44 patients per month, to reach 80 patients in 18 months.

During the appointment with the surgeon, he will explain the study design. Rights of the patients will also be specified during this appointment and eligibility will be checked. All patients will receive the information notice and an exemplary of the consent form.

5 EXPERIMENTAL STRATGIES

5.1 Study strategy

- **Definition and description of the strategy**

The individualized physical activity intervention in an home based setting involves personalizing the patients' physical activity in their usual living conditions. For this purpose, they will be equipped with a connected activity bracelet that measures their daily step count. A reassessment of daily goals will be conducted every two weeks via phone, based on the achievement or non-achievement of the goals set for the previous two weeks and the

patient's feedback regarding the objectives. Additionally, the use of the connected bracelet allows the patient to engage in physical activity without having to travel to the hospital or rehabilitation center. The program enables them to incorporate physical activity into their daily lives without radically changing their routines (they can engage in walking as a leisure activity or choose to walk for errands, etc.).

- **Justification of the strategy**

Physical activity (PA) offers numerous benefits for patients with atherosclerotic plaques, including a reduction in carotid intima-media thickness (cIMT), circulating inflammation, oxidative stress, and LDL levels, as well as an increase in circulating HDL levels. Furthermore, in a previous cross-sectional study conducted by our team (6), the most active patients were found to have a higher occurrence of non-hemorrhagic plaques.
Engaging in physical activity in an home based setting has an advantage over a protocol that involves travel: patients have a higher adherence rate to physical activity at the end of the protocol (6 months later) (49).
However, a physical activity protocol has never been tested on this type of patients.

- **Contraindications description**

Contraindications to the practice of PA: cardiovascular pathologies (exclusion criterion) osteoarticular diseases.

6 GENERAL ORGANIZATION

6.1 Study calendar

Inclusion period: 18 months
Study duration for each patient: *6 months +/- 2 weeks*
Total duration of the study: *24 months*
Start of inclusions: *3rd trimester 2019*

The promotor must promptly inform the CPP (Committee for the Protection of Persons) and ANSM (French National Agency for Medicines and Health Products Safety) of the start date of the study if it is a Phase 1 study (effective start date = date of the first person's consent signature to participate in the research).
The promotor must also notify the CPP and ANSM of the study's end date within 90 days for Phase 1 research. The end date of the research corresponds to the completion of participation by the last person enrolled in the study or, if applicable, the term defined in the protocol.

6.2 General figure and recapitulative table

Recrutement

Patients with HR-IRM of the carotid plaque (ACABII or other) but without any indication of carotid endarterectomy surgery

Pre-inclusion : appointment with the surgeon who explain the protocol, check for inclusion and exclusion criterionsand give the informed consent form.

Included / **Non Included**

Inclusion (18 months)

Inclusion : signature of the consent form, connected wristlet

Start of the study tests:

Questionnaires :
- Levels of PA and sendentary behavior
- Alimentation
- Quality of life

Clinic data:
- Age, height, wright, BMI, BP, HR, hips circumference
- Drug treatment
- Medical history

Tests/mesures:
- Maximal isometric quadriceps strenght test
- 6minutes walk test/VO2
- Blood sample (19 mL): flux cytometry, rheologie, coagulation, bioassays
- Steps/day

Start of the intervention

Randomization

Interventional group : bimensual phone call for reevaluation of objectives

Control group

6 months

End of study tests:

Questionnaires :
- Levels of sendentary behavior
- Alimentation
- Quality of life

Clinic data:
- Age, height, wright, BMI, BP, HR, hips circumference
- Drug treatment
- Medical history

Tests/mesures:
- Maximal isometric quadriceps strenght test
- 6minutes walk test/VO2
- Blood sample (19 mL): flux cytometry, rheologie, coagulation, bioassays
- Steps/day

End of the intervention

3 months

Analyses:

Biological mecanisms:
- IPH with MRI
- Monocytes phenotype
- Oxidative stress
- Inflammation

Physical activity
- 6minutes walk test/VO2
- Macimal quadriceps strenght
- Steps/day
- Questionnaires PA/sedentary behavior

Anthropometry / questionnaires:
- Weight, BMI, Hip circomference, leg volume, Alimentation, quality of life

ETAPES	***V1 Inclusion***	***V2 Start of the inclusion***	***V3 Phone calls***	***V4 End of the study***
Moment Actions	***Day -15***	***Day 0***	***Every 15 days***	***M6 +/- 15 days***
Consent form	*X*			
history	*X*			
Clinical exam[1]	*X*			
Paraclinical exams[2]		*X**		*X**
Biological analysis[3]		*X**		*X**
Randomization	*X*			
Protocol explanations/ connected device explanations	*X**	*X**		
Observance			*X**	*X**
Research of adverse events			*X*	*X*
Phone calls			*X*	

[1]Clinical exam: blood pressure, heart rate, ischemic events, laterality and degree of stenosis, obesity, type 2 diabetes, arterial hypertension, dyslipidemia, statins or anti-coagulant drugs.
[2]Paraclinical exams: MRI, 6 minutes' walk test, maximal isometric quadriceps strength test, quality of life PA and sedentary behavior questionnaires*, age, height, weight, BMI, hip circumference, waist circumference, steps/day.*
[3]Biologcal analysis: coagulation, hemato/hemorheology, monocytes phenotype, Biological assay.
**Exams done specifically for the present research*

6.3 Study design

- Pre-inclusion meeting
- Inclusion meeting
- Intervention (only interventional group)
- End of the study meeting

6.3.1 Screening – Pre-inclusion

The surgeon will meet the patient after the ACABII study. If the surgery is not needed, the surgeon will propose the PACAPh protocol to the patient. If the patient accepts he will receive an appointment with the clinical research associate of the unit for the inclusion meeting.
Recruitment will be done over a period of 18 months. Patients identified during the meeting with the surgeon will receive an information notice explaining the objectives and the progress of the study. The patient will receive a double exemplary of the consent form, so he has the time to read it through. A sufficient delay will be given to the patient to accept the protocol. If he accepts, the investigator clinician will check all inclusion and exclusion criterions and paraclinical exams will be done. The surgeon will give a non-contraindication certificate to the practice of PA. an auscultation and basic variable (hearth rate, blood pressure) will be collected.

The consent form should be signed before the realization of any clinical or paraclinical exam realized for the purpose of the research.

If the patient accepts the protocol, he and the investigating clinician date and sign two exemplary of the consent form. One will be given to the patient and the other will be kept in the investigation notebook of the study.

- Clinical exam: age, height, weight, BMI, blood pressure, heart rate, ischemic events, laterality and degree of stenosis, obesity, type 2 diabetes, arterial hypertension, dyslipidemia, statins or anti-coagulant drugs.
- Paraclinical exams: MRI, 6 minutes' walk test, maximal isometric quadriceps strength test, anthropometric measures, quality of life PA and sedentary behavior questionnaires.
- Biological analysis: coagulation, hemato/hemorheology, monocytes phenotype, Biological assay.

6.3.2 Inclusion visit / Randomization

During this visit, the protocol will be explained again to the patient. The investigator clinician will ensure that the consent form is signed and that the randomization 1:1 in the PA or control group is done. In the same day, the patient will see the professional in PA, that will dispense the PA tests and the questionnaires of quality of life. Blood sample will also be taken.

All patients will receive a connected wristlet and a complete explanation of its functioning. The patient will learn how to visualize his daily steps count, and basic functions of the app. The professional in PA will insist on how it is important to wear the wristlet all day and all night.

The professional in PA will do an individualized meeting with the patients of the PA group, in order to determine their lifestyle habits, their preferences in PA, in order to determine a first objective in terms of steps/day, keeping in mind that the final goal is reach 6 000 steps/day, or increase by 30% in comparison with the initial evaluation. Patients of the PA group will wear the connected wristlet during all the study duration, in order to follow continuously their dally step count. Strategies will be elaborated with the patient, as favor stairs over the elevator, walk to the grocery store, etc. Patients of the control group will wear the connected wristlet during the 15 firsts days of the study and then will be asked to not change their usual habits during the 6 months.

Functional tests done at Day 0 will evaluate the level and physical capacities of all patients and will be realize at the Louis Pradel's hospital. MRI will be done in the radiology unit of the *Hospices Civils de Lyon*. Flux cytometry and bioassays will be realized in the laboratory *LIBM EA7424* who masters these techniques.

The following evaluations will be done at Day 0:

- Anthropometry: weight (kg), height (cm), waist circumference (cm) and hip circumference (cm) using standard techniques (standardization of the measures: waist circumference is measured at equidistance from the last rib and the iliac crest; hip circumference is measured at the tip of the pubis). These measurements will be done with a measuring tape, a gauge and a scale.
- PA and sedentary levels: Quantitative questionnaire of PA and sedentary behavior, functional tests: 6 minutes' walk tests with measurement of respiratory exchanges, including VO_2 (mL/min/kg), and a maximal isometric quadriceps strength test of the hip extensors (quadriceps).
- Steps/day: an explanation will be given about the connected device in order to ensure the good follow up of the daily steps during the first 15 days of the protocol.
- blood sample for rheology, coagulation, oxidative stress bio assay, inflammation, flux cytometry (19 mL: 1 citrated tube (1x3mL) 2 EDTA tubes (2x4mL) and 2 heparin tubes (2x4mL)).
- Values of the usual blood draw will be collected in the patient file.

➔ The results of the ACABII study will be collected in order to ensure the initial evaluation of IPH and usual blood markers.

All these measurements are detailed below.

Anthropometric measures

Anthropometric characteristics of each patient will be systematically measured before the beginning of the protocol. Thus, height and weight be measured the day of the experimentation. Fat per- centage will be calculated using the skin fold sum method equations of Stevens et al. (2014) (54). Lower limb volume will be measured using the Jones et Pearson (1969) (55) method, validated in children's and adults (56). This technique relies on truncated cones modeling. With this model, the measure of circumferences and height will allow the normalization of the maximal strength of each patient

Quality of life questionnaires

Cognitive capacities will be assessed using the Folstein mini-mental questionnaire (57) (Annexes 1). For those with a score greater than 27 [52], the following questionnaires will be completed. The daily amount of time spent being sedentary will be assessed by the Sedentary Behavior Questionnaire [53]. Patients will be asked to estimate the time spent sitting or lying during nine activities (i.e., work, television, computer, meals, listening music, with friends/family, travels, leisure, and nap) (15). Weekly PA levels and intensity will be assessed by the Global Physical Activity Questionnaire (GPAQ) characterizing walking trips or moderate to intense PA at work or during leisure time [54, 55] and expressed in [metabolic equivalent of task]-min/week.

In order to characterize the population, a questionnaire of nutrition and quality of life will be done(59) (16).

6 minutes' walk test

Walking endurance will be measured by the 6-min walking test (6MWT) which has been validated to reliably determine the maximum distance a patient is able to walk in 6 min [45] according to the American Thoracic Society (ATS statement (60)). It is a sub-maximal test often used in cardiovascular populations because of the low risks involved and its simplicity. It is a quick, individualized test that measure the distance walked for a patient on a flat and hard floor during 6minutes. Thus this test is conceived to reevaluate the limitation of physical capacities for patients (ATS statement (60)).

Patients will be asked to walk as far as possible for 6 min between two cones. The distance between the cones is usually 30m. however, it has been shown that this distance can vary from 15m to 50m without influence on the result of the test. According to our experience a distance of 21m has been set (61). A mark will be done every 3 meters and will be done in a corridor; thus, patients can rest against the wall if needed.

Before the test the patients will rest for 10 minutes, sitting. During this period, the 6 minutes' walk test will be explained to him and the dyspnea will be evaluated using a Borg's scale. The following parameters will be measured:

- Oxygen saturation SpO2 (%), with a pulse oximeter. SpO2 < 90% will put an end to the test.
- Heart rate (bpm),
- Systolic and diastolic blood pressure (mmHg) with a brachial tensiometer.
- Oxygen consumption (VO_2), production of CO_2 (VCO_2),

INSTRUCTIONS TO THE PATIENT:
« The goal of this test is to walk the greatest distance during 6 minutes, walking back and forth between the cones. Walking 6 minutes, is a long time, it will be an important effort. You will probably be breathless and exhausted. You can slow down, stop or rest if necessary. You can rest against the wall, but you have to resume the test as soon as possible. You will walk back and forth between these

cones. You just have to pivot quickly around the cones without marking the stop nor hesitating. I will show you. Look at me how I am doing the pivot without hesitation. We will start the test as soon as you are ready. »

To avoid early exertion, incentives are standardized:
- After the first minute « You are doing fine. You have 5 more minutes to walk ».
- 2 minutes: « keep up. You have 4 more minutes ».
- 3 minutes: « great. You have done half of the test »
- 4 minutes: « keep up. You have 2 more minutes »
- 5 minutes: « Great. You only have 1-minute left ».

At the end of the test, the distance walked, physiological constants measured before the test will be measured again immediately after the test and 10 minutes after the test.

<u>Maximal isometric strength test of the hip extensors</u> (quadriceps)

The lower limb strength will be measured using an isometric quadricep strength test on the dominant leg, while sitting on a chair with a knee angle of 90° and a hip angle of 110°. The warm up will consist of 10 sub maximal contractions of 5 seconds with 30 seconds of recovery in between. The last 3 contraction will be done on average at estimated 25%, 50%, 75% intensity of the maximal capacities. After 1 minute of recovery, the patient will do 3 maximal contractions of the hip extensors of the dominant leg for a duration of 3 seconds, with 1-minute recovery. This type of contraction is used to measure the maximal voluntary isometric strength of the patient (IMVC). 3 repetition are done to avoid learning effect. Patient will be asked to push the strongest and the quickest possible on the cuff as if they were trying to extend the knee. During each contraction the patient is vigorously encouraged by the team in order to reach the best performance possible. This test should not promote discomfort or pain, but if expressed, the test will be stopped.

<u>Hemato/hemorheologie</u>

For each patient, 2 EDTA tubes of 4mL re dedicated to the hematological and hemorheological parameters analysis. Analyses will be done extemporaneously at the hemato/hemorheology platform of our laboratory LIBM at the biochemistry laboratory – *UF Pathologie moléculaire du globule rouge (Hôpital Edouard Herriot, HCL, Lyon*).

Hematological parameters (complete blood count) will be done on the MICROS 60 (ABX, Horiba). Blood viscosity will be measured with a cone/plate viscosimeter (Wells-Brookfield, cone CPE-40) at 3 different shear rates (45, 90 et 225 s^{-1}). Hematocrit will be measured by microcentrifugation. The index of hemorheological oxygenation will be calculated with the hematocrit values and the viscosities at different shear rates. Deformability of the red blood cells will be measured by ektacytometry (LORCA) at 9 shear stress (de 0,3 à 30 Pa). Aggregation and disaggregation of the red blood cells are studied with syllectometry (LORCA), after hematocrit adjustment 40%. All measures will be done in agreement with the international guide of good practices in hemorheology (65).

<u>Coagulation</u>

Venous citrated blood will be used for ex vivo clot formation according to protocols from)previous studies (66, 67). A complete blood count will be done on total blood samples. Blood clots will be analyzed with scanning electron microscopy. Blood clot formation, contraction and lysis will be explored by rotative thromboelastometry (ROTEM). Finally, in order to investigate the

elastic properties of the blood clot, we will realize ultrasound elastography (1D et 2D). All these measures will characterize the structure and ultrastructure of the venous blood clot.

Flux cytometry of monocytes

Two 3-mL heparin tubes will be used to determine monocytic phenotype. Peripheral blood mononuclear cells will be isolated from whole blood using a Ficoll gradient. The labeling procedure will include incubation with a Fc receptor blocking solution for 30 min to saturate monocyte Fc receptors. FITC-conjugated anti-CD14, PE-conjugated anti-CD16, and APC-conjugated anti-CD142 antibodies will be added for 30 min at 4°C. Controls included isotype- matched antibodies. The analysis will be performed by flux cytometry in order to analyze the surface markers of 50 00 cells per sample.

Usual bioassay

One EDTA tube will be prescribed at the *Centre de Biologie Est de l'Hôpital Louis Prade*l in order to dose the total cholesterol; triglycerides, HDL-cholesterol, LDL-cholesterol, platelets, fasting glucose, plasmatic fibrinogen. Dosage will be performed at 15days from any inti-inflammatory treatment.

MRI of the carotid plaque

MRI will be done by the radiologist of the unit of *Groupement Hospitalier Est*. tThe analyze will be done on a 3T-HR-MRI (Ingenia scanners, Philips Healthcare, Best, The Netherlands) device with a double surface coil (Sense Flex S, Philips Healthcare, Best, The Netherlands) positioned on the top of the mandible. Preliminary picture will be done to precisely determine the carotid bifurcation localization. Then time-of-flight (TOF), proton-density (PD) and time of longitudinal relaxation (T1 3D PSIR) sequences will be done perpendicularly to the carotid axis. Before and after gadolinium injection (Prohance, Bracco Imaging France, France) a TOF sequence of the supra-aortic trunks will be done with a magnetic resonance angiography with gradient echo and a reconstruction in 3D followed 5 minutes later by the T1 sequence. The total duration of the acquisition is 45 minutes. A fat suppression filter will be applied on every sequence and all images are acquired with a 18 centimeter field. Before analysis, the image quality will be scored from 0 to 4 (0: no image, 1: poor quality, 2: moderated quality, 3: good quality and 4: excellent quality) In function of the signal-to-noise ratio. Two experimented readers will evaluate the images in double-blind.

At the inclusion visit le patients will not realize the MRI nor the usual bioassay, because it already has been done during the ACABII trial. Nevertheless, all these measurements will be done at the end of the study specifically for research purposes.

Anthropometric measurements will last 10 minutes, questionnaires 20 minutes, functional tests 30 minutes for a total of 30 minutes for each patient.

6.3.3 Phone call follow up

The interventional group will be followed during 6-months for their PA, the other group will not be having any particular incentive.

During the first week, a phone call will be made with the patient to ensure the good use of the connected device and to answer eventual questions. Other additional phone calls can be added at the request of the patient.

The goal of this intervention in PA is to propose a PA support as therapeutic treatment, when the surgery is not an option for the patients. Only using connected wristlets, our intervention is accessible to all patients.

The intervention will consist in a 6-month home based PA individualized in function of the patient's capacities. The PA intervention is deliberately vague and accessible to all patients, so they can adapt PA at home in agreement with his wishes and preferred practices. PA evaluations and follow-up will be done by a professional in PA (master, 5 years of studies in sport science).

The PA intervention consists in giving the recommendations of 6000 steps /day measured by the connected wristlet to the patients of the PA group, in order for them to maintain or increase their active behavior and the subsequent benefits on health (for example, diminution of cardiovascular risk). The goal will also be to reduce the time spend having a sedentary behavior in the PA group. In order to reach this objective, patients of the PA group will have to walk at least 30 minutes per day. And/or increase their daily trips in the everyday life (climb stairs, walk to the grocery store, etc.).

In the PA group, after the first evaluation of the daily steps (day 0 to day 15) an objective in the daily steps will be set for a month.

Th data collected by the connected wristlet will be harvested by the regular transfer of the watch data to the mobile application. This application can be used by the patient to follow his activity. In order to respect the confidentiality of the data, an anonymized email address will be created for each patient, with a personalized identification and password, only known from the patient and the investigator, so he can follow the daily steps evolution. If patients do not possess a smartphone, the laboratory will lend a smartphone to the patient, for the duration of the study. In order to respect the progressivity of the intervention, the daily steps goal will be reevaluated twice a month and the difficulty of reaching these objectives will be assessed by a RPE scale. With these indicators, the objectives will be reevaluated twice a month by phone call.

Duration, frequency and intensity of exercises recommended will be individualized for each patient of the PA group in agreement with the physical capacities of each patient (estimated with the MET-hour/week and the results of the 6minutes walk test), his eventual history as a sportsman, his treatment and preferences. If the patient of the PA group realized the intervention without difficulties, the duration, frequency and intensity of the PA will be increased if he thinks he is capable.

6.3.4 End of the study visit

The patient will meet the professional in PA, who will administer physical capacities tests and quality of life questionnaires. Blood draw and HR-MRI will be done on the same day.

Functional capacities test will be done at M6 (end of the study) for all patients. MRI will be performed at the radiology unit of the *hospices Civils de Lyon*. Flux cytometry and bioassay will be done at the laboratory LIBM EA7424 who masters these techniques.

15 days (+/- 2 days) before the end of the study visit, the connected wristlets will be send to the patients of the control group for the 15 days of final evolution of their daily steps.

<u>The following evaluations will be done at Month 6:</u>

- MRI of the carotid plaque (see paragraph 7.3.2)
- Anthropometry: weight (kg), height (cm), waist circumference (cm) and hip circumference (cm) using standard techniques (standardization of the measures: waist circumference is measured at equidistance from the last rib and the iliac crest; hip circumference is measured at the tip of the pubis). These measurements will be done with a measuring tape, a gauge and a scale.
- PA and sedentary levels: Quantitative questionnaire of PA and sedentary behavior, functional tests: 6 minutes' walk tests with measurement of respiratory exchanges, including VO_2 (mL/min/kg), and a maximal isometric quadriceps strength test of the hip extensors (quadriceps).
- Steps/day: an explanation will be given about the connected device in order to ensure the good follow up of the daily steps during the first 15 days of the protocol.
- Blood sample for rheology, coagulation, oxidative stress bio assay, inflammation, flux cytometry (19 mL: 1 citrated tube (1x3mL) 2 EDTA tubes (2x4mL) and 2 heparin tubes (2x4mL)).

- A prescription will be given to the patient in order to realize usual bioassay at the *Centre de Biologie Est de l'Hôpital Louis Pradel.*

Duration of the evaluations is set as 1h for the MRI, 10 minutes for the anthropometric measures, 10 minutes for the questionnaires and 30 minutes of functional tests resulting in 2h appointments with the patient.

The study will be done once all patient has done all the visits.

6.4 *Rules of temporary or definitive study termination*

- Termination of a person's participation in the research: Subjects may withdraw their consent and request to exit the study at any time, for any reason. In the event of early withdrawal, the investigator must document the reasons as comprehensively as possible. The investigator may temporarily or permanently interrupt a subject's participation in the study for any reason that would best serve the subject's interests, particularly in the case of serious adverse events. In the event that a subject is lost to follow-up, the investigator will make every effort to re-establish contact with the individual. If consent is withdrawn, the data collected up to the date of withdrawal will be analyzed.

- Termination of part or all of the research: The study may be prematurely terminated in the event of unexpected serious adverse events requiring a review of the strategy's profile. Similarly, unforeseen events or new information related to the investigative method, which would likely prevent the study objectives from being achieved, may lead the promotor to prematurely terminate the study. The Hospices Civils de Lyon reserve the right to interrupt the study at any time if the inclusion objectives are not met. In the event of premature termination of the study for safety reasons, the promotor will provide the information to the ANSM and CPP within a period of 15 days.

6.5 *Collection of biological samples*

Usual care analyses: 1 EDTA tube (only at the end of the study visit). Biological analysis of oxidative stress , inflammation, hemato/hemorheology and coagulation markers: 1 citrated tube, 2 EDTA tube and 2 heparin tubes at day 0 and 6 months.

**Blood samples**	**Biological mechanisms**	**Biomarkers**	**Method**	**Volume Of blood**
PLASMA	DNA oxidation	8 OHDG	Kit Elisa	2 x 20 µl
	AGPI oxidation	MDA	Spectrometry	2 x 50 µl
	Protein oxidation	AOPP	Spectrometry	2 x 50 µl
	Antioxidant enzymes activity	SOD	Enzymology	2 x 20 µl
		GPX		2 x 20 µl
		Catalase		2 x 20 µl
	NO Metabolism	NO3 + NO2	Griess method	8 x 30 µl
	Inflammation	IL-1B		
Sang total	Rheology (Aggregation, deformability)			1 x 4mL

	Blood clot kinetics	Clotting time, clot firmness time clot firmness at different time points, clot lysis at 30 and 60 minutes	Rotative thromboelast ometry (ROTEM)	1 x 3mL
	ex-vivo clot formation	Quantity of red blood cells extracted from the clot, weight of the clot	N/A	
	Clot elasticity	Elasticity index	Ultrasound elastography	
	Usula bioassay	Total cholesterol, triglycerides, HDL-cholesterol, LDL-cholesterol, platelets, fasting glucose, plasmatic fibrinogen	Dosages	1 x 4 mL
Monocytes	Phenotype	CD14/CD16	Flux cytometry	2x 4mL

5 tubes will be needed at each blood draw at the *Louis Pradel* hospital, by a nurse of the vascular unit: 1 citrated tube (1x3mL), 2 EDTA tubes (2x 4mL) and 2 heparin tubes (2x4mL). These tubes will be directly processed at the *Centre de Biologie et Physiologie Est*

The centrifugation of one EDTA tube will be done in the half hour following the blood draw in order to collect the plasma and will be frozen at -80°C at the *Centre de Biologie et Physiologie Est*. Other tubes will go the Edouard Herriot hospital. The tubes will be analyzed at the *Laboratoire Interuniversitaire de Biologie de la Motricité*. Hemato/hemorheology, coagulation, monocytes isolation, and flux cytometry should be done on the same day as the blood draw

Each tube will be immediately identified with the anonymization number given to each patient at inclusion.

Samples will be kept until the end of the study at the *Centre de Biologie et Physiologie Est.* After analyses, the remaining samples will be destroyed.

7 SECURITY EVALUATION

7.1 Definitions

According to the article R1123-46 of the French public health code.

7.1.1 Adverse event

Any harmful occurrence in a person participating in a research involving human subjects, whether or not related to the research.

7.1.2 Severe adverse event (EIG)

Any event or adverse effect:
- That results in death; or
- That endangers the life of the person participating in the research; or

- That requires hospitalization or prolongation of hospitalization; or
- That causes significant or long-lasting disability or impairment; or
- That results in a congenital anomaly or malformation; or
- Any other medically significant event that does not meet the qualifications listed above:
  - But may be considered "potentially serious," including certain biological abnormalities;
  - Or medically relevant event according to the judgment of the investigator;
  - Or an event requiring medical intervention to prevent progression to one of the aforementioned states.

For example, these events may include intensive treatment in hospital emergency departments or at the participant's home for allergic bronchospasm, seizures, or coagulation disorders.
The term "endangering life" is reserved for an immediate life-threatening situation at the time of the adverse event, regardless of the corrective or palliative therapeutic consequences.
Some circumstances requiring hospitalization may not meet the severity criterion.
Refer to paragraph 8.2.3 for serious adverse events that do not require immediate reporting to the promotor.

7.1.3 Adverse effect (EI)

Any harmful and undesirable reaction resulting from research involving a human subject in its entirety.

7.2 Investigator responsibility

7.2.1 Procedures for the detection and collection of adverse events.

All adverse events must be actively searched for, reported, recorded, managed, and evaluated from the first visit (baseline, Day 0) until the end of the study and until their resolution.

All adverse events will be documented on the adverse event data collection forms in the study observation booklet. Each observed adverse event will be individually recorded. The intensity of adverse events will be determined as follows:

- Mild (Grade 1): No interference with the patient's daily activities.
- Moderate (Grade 2): Moderate interference with the patient's daily activities, but still acceptable.
- Severe (Grade 3): Significant interference with the patient's daily activities and deemed unacceptable.
- Life-threatening (Grade 4).
- Death (Grade 5).

All adverse events must be graded.

All adverse events of severe intensity, life-threatening, or resulting in death (Grade 3 or higher) are considered SERIOUS and must be promptly reported to the promotor.

7.2.2 EIG notification

The investigator evaluates each adverse event in terms of its severity.

The investigator must promptly notify the promotor, no later than 24 hours from the day of awareness, of all serious adverse events and serious incidents that occur in the trial, except for those specified in the protocol as not requiring immediate notification.

This initial notification must be provided in written form and followed by one or more detailed written follow-up report(s) within 8 days of the initial notification.

The investigator must fax a dated and signed Serious Adverse Event (SAE) notification form to +33 4 72 11 51 90 as soon as they have the minimum required information to report an SAE:

- A notifier
- A subject

- An investigational product (if applicable)

- An adverse event

The investigator must thoroughly document the event (including copies of laboratory results or examination and hospitalization reports that provide information on the serious event, including relevant negative results, ensuring the documents are anonymized and include the patient's number and code), the medical diagnosis, and establish a causal relationship between the serious adverse event and the medication(s).

The investigator must continue to follow up with the patient who experienced an SAE until its resolution, stabilization at a level deemed acceptable by the investigator, or return to the pre-existing state, even if the patient has exited the trial, and inform the promotor by fax at +33 4 72 11 51 90 using the provided form (check the box: ✓ follow-up).

7.2.3 Serious Adverse Events Not Requiring Immediate Notification to the Promotor

Adverse events should be recorded in the observation log.

7.2.4 Adverse event of particular interest

Some events require special monitoring and will be reported as a Serious Adverse Event (at the request of the promotor, the Independent Monitoring Committee, a pharmaceutical company, or the relevant authorities).

7.2.5 Evaluation of causality

The investigator must assess the causal relationship between adverse events and the research. The causal relationship is binary (related / unrelated).

7.2.6 *Period for immediate notification of SAEs to the promotor by the investigator and procedures for monitoring serious adverse events.*

The investigator must promptly notify the promotor of serious adverse events:
- From the patient's inclusion (date of first consent signature) to [DATE OF PROCEDURE];
- Until [END OF PATIENT'S PARTICIPATION OR UP TO/1 MONTH AFTER THE LAST PROCEDURE] in the study;
- Without a time limit for serious adverse events related to the research.

7.3 <u>Responsibility of the promotor</u>

7.3.1 Declaration to authorities

The promotor will report to the ANSM:
- In case of a life-threatening situation or death of the subject: all suspicions of unexpected serious adverse events without delay from the day the promotor becomes aware of them, and relevant additional information to be submitted as a follow-up report within 8 days upon receipt of the follow-up.
- For all other unexpected serious adverse events: no later than 15 days from the day the promotor becomes aware of them, and relevant additional information to be submitted as a follow-up report within a new deadline of 8 days upon receipt of the follow-up.

The promotor will also report to the ANSM and the CPP any new safety information and, if applicable, the measures taken without delay from the day the promotor becomes aware of them, and relevant additional information to be submitted as a follow-up report to the ANSM within 8 days from the initial notification.

FOR ALL

The promotor will also prepare an annual safety report (ASR) to be submitted to the ANSM and the CPP within 60 days after the study's anniversary date.

7.3.2 Description of research-related adverse effects (safety reference for the promotor's assessment of expected/unexpected nature)

Adverse effects associated with the physical activity program and physical activity tests are typical injuries related to the practice of physical activity, such as musculoskeletal disorders (sprains, tendinitis, etc.). The coronary risk is very low for these patients since gentle physical activity (walking) is recommended. Additionally, gentle to moderate physical activity is protective against coronary-related deaths (Léon 1987).

The expected adverse events following the MRI are infrequent and primarily related to the contrast agent injection, including:

- Hypersensitivity reactions
- Injection site reactions
- Pruritus, rash
- Hypotension, hypertension
- Headaches
- Dizziness
- Nausea
- Chest pain
- Myocardial ischemia and/or myocardial infarction, particularly in patients with underlying coronary disease (especially with SONOVUE®)
- Cardiac arrhythmia (especially with SONOVUE® if used in combination with dobutamine)
- Risks associated with blood sampling: hematoma, bleeding, injection site pain

7.4 Independent surveillance comity

The Independent Data Monitoring Committee is an advisory committee responsible for providing the Promotor of a clinical trial with an opinion on the conduct of the trial. For this purpose, it reviews all issues that may arise in the trial, including scientific, ethical, and safety concerns that could affect the risk/benefit ratio. Following this review, it provides written recommendations to the Promotor. These recommendations may include the continuation, modification, or termination of the trial. The Promotor retains the decision-making authority regarding the measures to be implemented following the recommendations of the Independent Data Monitoring Committee. The operating procedures of the committee are described in a charter signed by the committee members at the start of the research. It is composed of at least two clinical experts and one methodologist/biostatistician.

An adverse event is a harmful and unintended reaction. According to Article L1123-10, the provisions concerning vigilance for each product or practice being investigated apply. The investigator must report any adverse event according to the quality and care management procedures of their institution. According to Article L1413-14, any healthcare professional or healthcare facility that identifies a nosocomial infection or any

other serious adverse event related to care provided during investigations, treatments, or preventive actions must report it to the Director General of the Regional Health Agency.

Healthcare-related adverse events should be reported on the portal for reporting healthcare-related adverse events: www.signalement-sante.gouv.fr

The investigator notifies the Promotor through the observation notebook of all adverse events and abnormal results of medical laboratory tests defined in the protocol as relevant for the evaluation of participant safety. Please refer to the outcome measures.

---

8 STATISTICS

8.1 Population needed

In our cross-sectional study (6) asymptomatic patients with a carotid atherosclerotic plaque had an IPH prevalence of 57%. We hypothesize that an increase of 100% of the level of PA (600 vs 1200 MET-min/week) decreases the prevalence of plaques presenting IPH by 50%. The R software (version 3.3.2) allowed us to calculate a population of 40 patients in each group (power 80% and an α bilateral risk of 5%) for a total of 80 over 18 months.

The active file of patients diagnosed with a carotid stenosis > 50% but not operated is on average 150 patients per year at the *hospices civils de Lyon*. In order to estimate the number of patients potentially eligible over 18 months and counting on 50% rejection, it is planned to recruit 4.44 patients per month, to reach 80 patients in 18 months. Patients will be randomized in 2 groups:

- Interventional group: Individualized, home-based PA (increase or stabilization of the daily step count during the 6 months)
- Control group: no modification of daily step count during the 6 months

The comparison of the groups will be focused on the degree of IPH. The trial is a difference trial within a two-tailed test formulation. The PA (interventional) group will receive a connected wristlet, and the daily step goal will be re-evaluated every 15 day. No particular incentive was given to the control group.

We hypothesize that in the interventional group, the proportion of patients reducing their IPH levels are 26% (6) et we consider that the strategy is interesting if the proportion of patients reducing their IPH level increased by 33% (6).

In this case the calculus of the size effect with a risk α of 5%, 44 patients should be included in each group, 88 on total. With an attrition rate of 2%, 45 patients should be included, 90 on total.

8.2 Statistical method Ananlysis

The statistical analyses will be conducted by the Direction of Clinical Research and Innovation of the Hospices Civils de Lyon (Lyon). In general, qualitative data will be described by their frequency and percentage. The number of missing data will be reported if necessary. Quantitative data will be described by the number of patients, mean, standard deviation, median, minimum and maximum values, and the number of missing data if necessary.

The data will be processed using GraphPad Prism software. Univariate analysis will compare mean differences observed in different physiological, biological, and clinical measures between the different groups. After checking the normality of the distribution of the variables studied (Kolmogorov-Smirnov test), these mean comparisons will be performed using the Student's t-test for independent samples (Mann-Whitney U test if necessary) for comparisons between 2 groups, and a one-way ANOVA followed by Tukey's post-hoc tests (Kruskal-Wallis test + Dunn's test if necessary) for comparisons among multiple groups.

Correlation analysis will be conducted between physiological, biological, and clinical data in both groups and within each group.

Subsequently, multivariate analysis using multiple linear regressions will be performed to identify factors independently associated with HIP in patients with carotid atherosclerotic plaque, taking potential confounding factors into account.

The analyses will be conducted on all patients included in the study.

For all tests, a significance level of 5% (α=0.05) will be used.

Finally, a coding system for missing data will be established during data collection. These data will not be included in the analyses but will be described in the results.

Any subsequent modifications to the analysis plan will be systematically validated by the steering committee.

8.3 Missing data

The number of missing data will be specified if necessary.

8.4 Management of modifications made to the analysis plan

A detailed statistical analysis plan will be developed prior to database lock. It will consider any protocol modifications or unexpected events that occur during the study and have an impact on the analyses outlined above. The planned analyses may be supplemented in line with the study objectives.
Any subsequent modifications made to the statistical analysis plan must be justified and documented in a revised version of the document. These deviations from the analysis plan will be reported in the final study report. All documents will be retained in the study file.

8.5 Biostatistician

Vincent PIALOUX

Softwares :
- BDIS software
- GraphPad
- Matlab
-

9 RESEARCH SURVEILLANCE

To ensure regular monitoring of the study progress, a steering committee has been established.

This committee will address any logistical and scientific issues that may arise during the study.

The committee, which will meet on a quarterly basis, consists of the following individuals or their representatives:

- Pr Antoine Millon, principal investigator
- Pr Vincent Pialoux, physiologist, Université Claude Bernard Lyon 1

ACCESS RIGHTS TO DATA AND SOURCE DOCUMENTS

9.1 Access to data

In accordance with Good Clinical Practice (GCP):

- The promotor is responsible for obtaining the agreement of all parties involved in the research to ensure direct access to all research sites, source data, source documents, and reports for the purpose of quality control and auditing by the promotor.

- Investigators will make available to individuals responsible for monitoring, quality control, or auditing of research involving human subjects the strictly necessary individual documents and data for such control, in accordance with applicable legislative and regulatory provisions (articles L.1121-3 and R.5121-13 of the French Public Health Code).

9.2 Source documents

Source documents are defined as any original document or object that provides evidence of the existence or accuracy of a recorded data or fact during the clinical study. They will be retained for 25 years by the investigator or by the hospital if it is a hospital medical record.

Listed below are the types of source documents within the scope of the research:

Medical records
Biological test result sheets
MRI reports
Case Report Forms (CRFs)

9.3 Data confidentiality

In accordance with the provisions regarding data confidentiality accessed by individuals responsible for quality control in research involving human subjects (Article L.1121-3 of the French Public Health Code) and the provisions concerning the confidentiality of information related to trials, participants, and obtained results (Article R. 5121-13 of the French Public Health Code), individuals with direct access to the data will take all necessary precautions to ensure the confidentiality of trial-related information, including the identity of participants and the obtained results.

These individuals, like the investigators themselves, are bound by professional secrecy (in accordance with the conditions defined by Articles 226-13 and 226-14 of the French Penal Code). During or after the research involving human subjects, the data collected on participants and transmitted to the promotor by investigators (or any other specialized personnel) will be rendered anonymous.

Under no circumstances should the data contain the clear names or addresses of the individuals involved.

Anonymization of patients will be carried out as follows:
- Two initials: the first letter of the last name and the first letter of the first name
- The patient's inclusion number corresponding to the chronological order number.

For each patient included in the study, an anonymized paper observation chart will be used.

The promotor will ensure that each research participant has provided written consent for access to their individual data strictly necessary for quality control of the research.

10 CONTROL AND QUALITY INSURANCE

A Clinical Research Associate (CRA) appointed by the promotor will ensure the proper conduct of the study, the collection of data generated in writing, their documentation, recording, and reporting, in accordance with the Standard Operating Procedures implemented within the Clinical Research and Innovation Department (DRCI) of the Hospices Civils de Lyon, and in compliance with Good Clinical Practice as well as applicable legislative and regulatory provisions.

The investigator and members of their team agree to be available during regular Quality Control visits conducted by the Clinical Research Associate. During these visits, the following elements may be reviewed based on the level of monitoring adapted to the study and determined in accordance with the Promotor's Standard Operating Procedures:

As part of the monitoring plan:
- Informed consent
- Adherence to the study protocol and defined procedures
- Quality of data collected in the observation chart: accuracy, missing data, consistency of data with source documents
- Management of investigational treatments
- Reporting of serious adverse events.

Each visit will be documented in a monitoring report provided in written form to the investigator of the visited site and the research coordination structure.

11 ETHICAL CONSIDERATIONS

11.1 Competent autorities

The study protocol, informed consent form, and patient information sheet will be submitted for review to the Ethics Committee for XX (Specify the number of the designated Ethics Committee after review).
The notification of the favorable opinion from the Ethics Committee will be sent to the study promotor and to the ANSM (French National Agency for Medicines and Health Products Safety). The study promotor will also submit a study authorization request to the ANSM.
The study promotor commits to initiating the study only after obtaining the favorable opinion from the Ethics Committee and the study authorization from the ANSM.

11.2 Substantial modifications

In the event of a substantial modification made to the protocol by the investigator, it will require approval from the promotor. The promotor must obtain a favorable opinion from the Ethics Committee and authorization from the ANSM within their respective jurisdictions before implementing the modification. If necessary, a new consent will be obtained from the individuals participating in the research.

11.3 Patient Information and Written Consent Form

Patients will be fully and honestly informed, in understandable terms, about the objectives and constraints of the study, potential risks involved, necessary monitoring and safety measures, and their rights to refuse participation in the study or to withdraw at any time.
All this information will be provided in an information and consent form given to the patient. The patient's voluntary, informed, and written consent will be obtained by the investigator or a representing physician prior to the final inclusion in the study. A copy of the signed information and consent form by both parties will be given to the patient, while the investigator will retain the original.

11.4 Conformity declaration

The promotor and investigator commit to conducting this research:
- in accordance with the protocol,
- in accordance with current French and international good clinical practices,
- in accordance with current legislative and regulatory requirements in France and at the international level.

11.5 Exclusion period

No exclusion period

11.6 Compensation for subjects and enrollment in the national registry for individuals participating in interventional research on human subjects under Article 1°

The patients will keep the connected bracelet for their personal use at the end of the study. Since the research pertains to the participants' pathological condition, enrollment in the national registry for Volunteers participating in Biomedical Research (VRB) is not required.

There is no provision for compensating the volunteers who agree to participate in the study. The activity tracker bracelet will be given to them at the end of the protocol.

12 DATA GESTION AND STORAGE

12.1 Observation book

The observation log will only include data necessary for analysis and publication. All other patient-related data necessary for their follow-up outside of the study will be collected in their medical record.

All information required by the protocol must be recorded in the observation logs. The data should be collected as they are obtained and explicitly recorded in these logs. Each missing data point should be coded.
This electronic observation log will be implemented in each center using an internet-based data collection platform. A user guide for the tool will be provided to the investigators.
By filling out the observation log online, the investigator allows the study coordination center to quickly and remotely access the data. The investigator is responsible for the accuracy, quality, and relevance of all entered data. Furthermore, during data entry, the system performs immediate consistency checks. As such, the investigator must validate any changes made to data values in the case report form (CRF). These modifications are documented in an audit trail. Additional justifications may be included as comments. At the end of the study, a printed copy will be requested, authenticated (dated and signed) by the investigator. A copy of the authenticated document for the promotor should be archived by the investigator.

12.2 Data gestion

Data entry will be performed by an operator from the vascular surgery department using a computerized data entry mask generated with SAS® software. The data will be anonymized according to the data management plan jointly defined by the coordinating investigator, methodologist, data manager, and statistician.

Data validation follows the established process outlined in the study coordination center's procedures. The raw data will be frozen in XML format and as SAS tables.

A complete backup of the data will be conducted every evening, retained for 4 weeks, and then archived monthly on the hard drive.

12.3 CNIL

This study falls under the scope of the "Méthodologie de Référence" (MR-001) in accordance with Article 54, paragraph 5 of Law No. 78-17 of January 6, 1978, as amended, relating to information technology, files, and civil liberties. This change was approved by decision on January 5, 2006, and modified on July 21, 2016. The Hospices Civils de Lyon, the study's promotor, have signed a commitment to comply with this "Méthodologie de Référence."

12.4 Archives

For all research involving human subjects, the duration of retention is 25 years.

The following documents will be archived under the name of the study in the premises of the Vascular Surgery Department of the Hospices Civils de Lyon until the end of the practical utility period:
- Protocol and annexes, including any amendments
- Original signed information and consent forms
- Individual data (authenticated copies of raw data)
- Follow-up documents and correspondence related to the research
- Statistical analyses
- Final study report

The promotor will retain all study data for 25 years.

No relocation or destruction can take place without the consent of the promotor. At the end of the 25-year period, the promotor will be consulted regarding destruction. All data, documents, and reports may be subject to audit or inspection.

13 FINANCEMENT ET INSURANCE

13.1 Budget of the study

Exemple :
Les frais liés à cette recherche sont les suivants :

- *Intervention propre à l'étude : médicaments, dispositif…*
- *Examens relatifs au protocole : imagerie, biologie…*
- *Logistique : transports des prélèvements, CRB…*
- *Recrutement de personnel : ARC, TEC, IRC, etc…*
- *Contrôle qualité par un ARC mandaté par le promoteur*
- *Assurance de la recherche*
- *Gestion des données : cahier d'observation électronique, data-management, analyse statistique*
- *Frais divers : réunions, missions des coordonnateurs, papeterie, envois divers, …*

13.2 Insurance

The promotor has obtained insurance coverage for the entire duration of the study, ensuring their own civil liability as well as that of any physician involved in the study. They will also provide full compensation for any harmful consequences to the research participant and their beneficiaries, unless they can prove that the damage is not attributable to their fault or that of any other party involved, without taking into account the

actions of a third party or the voluntary withdrawal of the person who initially consented to participate in the research.

The insurance policy was obtained prior to the start of the study from Société Hospitalière d'Assurance Mutuelle, located at 18 rue Edouard Rochet, 69008 Lyon, under the number XXXXXX (to be completed by the DRCI).

14 PUBLICATION RULES

The scientific communications and reports related to this study will be prepared under the responsibility of the principal investigator, with the agreement of the associated investigators. The co-authors of the report and publications will include the investigators and clinicians involved, proportionate to their contribution to the study, as well as the biostatistician and associated researchers.

The publication guidelines will follow international recommendations (N Engl J Med, 1997; 336:309-315).

The study will be registered on an open-access clinical trials registry (clinicaltrials.gov) before the inclusion of the first patient.

15 REFERENCES BIBLIOGRAPHIQUES

1. Aouba A. Mortality data in France: the main causes of death in 2008 and trends since 2000 [Internet]. 2011 [cited 2017 Mar 10]. Available from: http://invs.santepubliquefrance.fr/beh/2011/22/beh_22_2011.pdf

2. Li F, Yang L, Yang R, Xu W, Chen F-P, Li N, et al. Ischemic Stroke in Young Adults of Northern China: Characteristics and Risk Factors for Recurrence. Eur Neurol. 2017 Jan 5;77(3–4):115–22.

3. Benjamin EJ, Blaha MJ, Chiuve SE, Cushman M, Das SR, Deo R, et al. Heart Disease and Stroke Statistics—2017 Update: A Report From the American Heart Association. Circulation. 2017 Mar 7;135(10):e146–603.

4. Mughal MM, Khan MK, DeMarco JK, Majid A, Shamoun F, Abela GS. Symptomatic and asymptomatic carotid artery plaque. Expert Rev Cardiovasc Ther. 2011 Oct;9(10):1315–30.

5. Michel J-B, Virmani R, Arbustini E, Pasterkamp G. Intraplaque haemorrhages as the trigger of plaque vulnerability. Eur Heart J. 2011 Aug;32(16):1977–85, 1985a, 1985b, 1985c.

6. Mury P, Mura M, Della-Schiava N, Chanon S, Vieille-Marchiset A, Nicaise V, et al. Association between physical activity and sedentary behaviour on carotid atherosclerotic plaques: an epidemiological and histological study in 90 asymptomatic patients. Br J Sports Med. 2019 Mar 6;

7. Nemerson Y. Tissue factor and hemostasis [published erratum appears in Blood 1988 Apr;71(4):1178]. Blood. 1988 Jan 1;71(1):1–8.

8. Chistiakov DA, Orekhov AN, Bobryshev YV. Contribution of neovascularization and intraplaque haemorrhage to atherosclerotic plaque progression and instability. Acta Physiol Oxf Engl. 2015 Mar;213(3):539–53.

9. Higher Daily Physical Activity Level Is Associated with Lower RBC Aggregation in Carotid Artery Disease Patients at High Risk of Stroke. - PubMed - NCBI [Internet]. [cited 2019 Mar 15]. Available from: https://www.ncbi.nlm.nih.gov/pubmed/29311973

10. Rogacev KS, Cremers B, Zawada AM, Seiler S, Binder N, Ege P, et al. CD14++CD16+ monocytes independently predict cardiovascular events: a cohort study of 951 patients referred for elective coronary angiography. J Am Coll Cardiol. 2012 Oct 16;60(16):1512–20.

11. Barnett HJM, Meldrum HE, Eliasziw M, North American Symptomatic Carotid Endarterectomy Trial (NASCET) collaborators. The appropriate use of carotid endarterectomy. CMAJ Can Med Assoc J J Assoc Medicale Can. 2002 Apr 30;166(9):1169–79.

12. den Hartog AG, Bovens SM, Koning W, Hendrikse J, Luijten PR, Moll FL, et al. Current status of clinical magnetic resonance imaging for plaque characterisation in patients with carotid artery stenosis. Eur J Vasc Endovasc Surg Off J Eur Soc Vasc Surg. 2013 Jan;45(1):7–21.

13. Delrieu L, Perol O, Friedenreich C, Febvey O, Martin A, Fervers B, et al. Advanced Stage Breast Cancer Lifestyle and Exercise (ABLE) Feasibility Study: Preliminary Results. The Breast. 2017 Nov 1;36:S66–7.

14. Armstrong T, Bull F. Development of the World Health Organization Global Physical Activity Questionnaire (GPAQ). J Public Health. 2006 Apr 1;14(2):66–70.

15. Rosenberg DE, Norman GJ, Wagner N, Patrick K, Calfas KJ, Sallis JF. Reliability and validity of the Sedentary Behavior Questionnaire (SBQ) for adults. J Phys Act Health. 2010 Nov;7(6):697–705.

16. Devlin NJ, Krabbe PFM. The development of new research methods for the valuation of EQ-5D-5L. Eur J Health Econ. 2013 Jul 1;14(1):1–3.

17. MRC European Carotid Surgery Trial: interim results for symptomatic patients with severe (70-99%) or with mild (0-29%) carotid stenosis. European Carotid Surgery Trialists' Collaborative Group. Lancet Lond Engl. 1991 May 25;337(8752):1235–43.

18. North American Symptomatic Carotid Endarterectomy Trial. Beneficial Effect of Carotid Endarterectomy in Symptomatic Patients with High-Grade Carotid Stenosis. N Engl J Med. 1991 Aug 15;325(7):445–53.

19. Naylor AR. Time to rethink management strategies in asymptomatic carotid artery disease. Nat Rev Cardiol. 2011 Oct 11;9(2):116–24.

20. Stein RA, Rockman CB, Guo Y, Adelman MA, Riles T, Hiatt WR, et al. Association between physical activity and peripheral artery disease and carotid artery stenosis in a self-referred population of 3 million adults. Arterioscler Thromb Vasc Biol. 2015 Jan;35(1):206–12.

21. Lee CD, Folsom AR, Blair SN. Physical activity and stroke risk: a meta-analysis. Stroke. 2003 Oct;34(10):2475–81.

22. Breneman CB, Polinski K, Sarzynski MA, Lavie CJ, Kokkinos PF, Ahmed A, et al. The Impact of Cardiorespiratory Fitness Levels on the Risk of Developing Atherogenic Dyslipidemia. Am J Med. 2016;129(10):1060–6.

23. Pigłowska M, Kostka T, Drygas W, Jegier A, Leszczyńska J, Bill-Bielecka M, et al. Body composition, nutritional status, and endothelial function in physically active men without metabolic syndrome--a 25 year cohort study. Lipids Health Dis. 2016 Apr 27;15:84.

24. Crouse SF, O'Brien BC, Grandjean PW, Lowe RC, Rohack JJ, Green JS. Effects of training and a single session of exercise on lipids and apolipoproteins in hypercholesterolemic men. J Appl Physiol Bethesda Md 1985. 1997 Dec;83(6):2019–28.

25. Greene NP, Martin SE, Crouse SF. Acute exercise and training alter blood lipid and lipoprotein profiles differently in overweight and obese men and women. Obes Silver Spring Md. 2012 Aug;20(8):1618–27.

26. Kim M, Yoo HJ, Kim M, Ahn HY, Park J, Lee S-H, et al. Associations among oxidative stress, Lp-PLA2 activity and arterial stiffness according to blood pressure status at a 3.5-year follow-up in subjects with prehypertension. Atherosclerosis. 2017 Feb;257:179–85.

27. Elosua R, Molina L, Fito M, Arquer A, Sanchez-Quesada JL, Covas MI, et al. Response of oxidative stress biomarkers to a 16-week aerobic physical activity program, and to acute physical activity, in healthy young men and women. Atherosclerosis. 2003 Apr;167(2):327–34.

28. Callegari A, Liu Y, White CC, Chait A, Gough P, Raines EW, et al. Gain and loss of function for glutathione synthesis: impact on advanced atherosclerosis in apolipoprotein E-deficient mice. Arterioscler Thromb Vasc Biol. 2011 Nov;31(11):2473–82.

29. Gieseg SP, Amit Z, Yang Y-T, Shchepetkina A, Katouah H. Oxidant production, oxLDL uptake, and CD36 levels in human monocyte–derived macrophages are downregulated by the macrophage-generated antioxidant 7,8-dihydroneopterin. Antioxid Redox Signal. 2010 Nov 15;13(10):1525–34.

30. Zoppini G, Targher G, Zamboni C, Venturi C, Cacciatori V, Moghetti P, et al. Effects of moderate-intensity exercise training on plasma biomarkers of inflammation and endothelial dysfunction in older patients with type 2 diabetes. Nutr Metab Cardiovasc Dis NMCD. 2006 Dec;16(8):543–9.

31. Gielen S, Sandri M, Erbs S, Adams V. Exercise-induced modulation of endothelial nitric oxide production. Curr Pharm Biotechnol. 2011 Sep;12(9):1375–84.

32. Microvascular Endothelial Dysfunction in Sedentary, Obese Humans Is Mediated by NADPH Oxidase: Influence of Exercise Training. - PubMed - NCBI [Internet]. [cited 2019 Mar 17]. Available from: https://www.ncbi.nlm.nih.gov/pubmed/?term=Microvascular+Endothelial+Dysfunction+in+Sedentary%2C+Obese+Humans+Is+Mediated+by+NADPH+Oxidase%3A+Influence+of+Exercise+Training

33. Lee J, Cooke JP. The role of nicotine in the pathogenesis of atherosclerosis. Atherosclerosis. 2011 Apr;215(2):281–3.

34. Ookawara T, Haga S, Ha S, Oh-Ishi S, Toshinai K, Kizaki T, et al. Effects of endurance training on three superoxide dismutase isoenzymes in human plasma. Free Radic Res. 2003 Jul;37(7):713–9.

35. Inoue N, Ramasamy S, Fukai T, Nerem RM, Harrison DG. Shear stress modulates expression of Cu/Zn superoxide dismutase in human aortic endothelial cells. Circ Res. 1996 Jul;79(1):32–7.

36. Ross R. Atherosclerosis--an inflammatory disease. N Engl J Med. 1999 Jan 14;340(2):115–26.

37. Kargarfard M, Lam ETC, Shariat A, Asle Mohammadi M, Afrasiabi S, Shaw I, et al. Effects of endurance and high intensity training on ICAM-1 and VCAM-1 levels and arterial pressure in obese and normal weight adolescents. Phys Sportsmed. 2016;44(3):208–16.

38. Yakeu G, Butcher L, Isa S, Webb R, Roberts AW, Thomas AW, et al. Low-intensity exercise enhances expression of markers of alternative activation in circulating leukocytes: roles of PPARγ and Th2 cytokines. Atherosclerosis. 2010 Oct;212(2):668–73.

39. Lakka TA, Laukkanen JA, Rauramaa R, Salonen R, Lakka HM, Kaplan GA, et al. Cardiorespiratory fitness and the progression of carotid atherosclerosis in middle-aged men. Ann Intern Med. 2001 Jan 2;134(1):12–20.

40. Sluimer JC, Kolodgie FD, Bijnens APJJ, Maxfield K, Pacheco E, Kutys B, et al. Thin-walled microvessels in human coronary atherosclerotic plaques show incomplete endothelial junctions relevance of compromised structural integrity for intraplaque microvascular leakage. J Am Coll Cardiol. 2009 Apr 28;53(17):1517–27.

41. Libby P. Inflammation in atherosclerosis. Nature. 2002 Dec 19;420(6917):868–74.

42. Van Craenenbroeck AH, Van Ackeren K, Hoymans VY, Roeykens J, Verpooten GA, Vrints CJ, et al. Acute Exercise-Induced Response of Monocyte Subtypes in Chronic Heart and Renal Failure. Mediators Inflamm [Internet]. 2014 [cited 2018 May 5];2014. Available from: https://www.ncbi.nlm.nih.gov/pmc/articles/PMC4283262/

43. Hankey GJ, Warlow CP, Molyneux AJ. Complications of cerebral angiography for patients with mild carotid territory ischaemia being considered for carotid endarterectomy. J Neurol Neurosurg Psychiatry. 1990 Jul;53(7):542–8.

44. Ogata A, Kawashima M, Wakamiya T, Nishihara M, Masuoka J, Nakahara Y, et al. Carotid artery stenosis with a high-intensity signal plaque on time-of-flight magnetic resonance angiography and association with evidence of intraplaque hypoxia. J Neurosurg. 2016 Jul 1;1–6.

45. Puppini G, Furlan F, Cirota N, Veraldi G, Piubello Q, Montemezzi S, et al. Characterisation of carotid atherosclerotic plaque: comparison between magnetic resonance imaging and histology. Radiol Med (Torino). 2006 Oct;111(7):921–30.

46. Mora S, Lee I-M, Buring JE, Ridker PM. Association of physical activity and body mass index with novel and traditional cardiovascular biomarkers in women. JAMA. 2006 Mar 22;295(12):1412–9.

47. Palmefors H, DuttaRoy S, Rundqvist B, Börjesson M. The effect of physical activity or exercise on key biomarkers in atherosclerosis--a systematic review. Atherosclerosis. 2014 Jul;235(1):150–61.

48. Ford ES. Does exercise reduce inflammation? Physical activity and C-reactive protein among U.S. adults. Epidemiol Camb Mass. 2002 Sep;13(5):561–8.

49. Zdziarski LA, Wasser JG, Vincent HK. Chronic pain management in the obese patient: a focused review of key challenges and potential exercise solutions. J Pain Res. 2015 Feb 9;8:63–77.

50. Poggio R, Gutierrez L, Irazola V, Rubinstein AL, Danaei G. Preventable Ischaemic Heart Disease and Stroke Deaths Attributable to Insufficient Physical Activity: A Comparative Risk Assessment Analysis in the Argentinian Population. 2017 Apr [cited 2019 Mar 17]; Available from: http://ri.conicet.gov.ar/handle/11336/40654

51. Kanejima Y, Kitamura M, Izawa KP. Self-monitoring to increase physical activity in patients with cardiovascular disease: a systematic review and meta-analysis. Aging Clin Exp Res. 2019 Feb;31(2):163–73.

52. Fitzgerald JD, Johnson L, Hire DG, Ambrosius WT, Anton SD, Dodson JA, et al. Association of objectively measured physical activity with cardiovascular risk in mobility-limited older adults. J Am Heart Assoc. 2015 Feb 18;4(2).

53. Petersen AMW, Pedersen BK. The anti-inflammatory effect of exercise. J Appl Physiol Bethesda Md 1985. 2005 Apr;98(4):1154–62.

54. Stevens J, Cai J, Truesdale KP, Cuttler L, Robinson TN, Roberts AL. Percent body fat prediction equations for 8- to 17-year-old American children. Pediatr Obes. 2014;9(4):260–71.

55. Jones PR, Pearson J. Anthropometric determination of leg fat and muscle plus bone volumes in young male and female adults. J Physiol. 1969 Oct;204(2):63P-66P.

56. Martin AD, Daniel M, Clarys JP, Marfell-Jones MJ. Cadaver-assessed validity of anthropometric indicators of adipose tissue distribution. Int J Obes Relat Metab Disord J Int Assoc Study Obes. 2003 Sep;27(9):1052–8.

57. Folstein MF, Folstein SE, McHugh PR. ‘Mini-mental state’. A practical method for grading the cognitive state of patients for the clinician. J Psychiatr Res. 1975 Nov;12(3):189–98.

58. Bull FC, Maslin TS, Armstrong T. Global physical activity questionnaire (GPAQ): nine country reliability and validity study. J Phys Act Health. 2009 Nov;6(6):790–804.

59. England CY, Andrews RC, Jago R, Thompson JL. A systematic review of brief dietary questionnaires suitable for clinical use in the prevention and management of obesity, cardiovascular disease and type 2 diabetes. Eur J Clin Nutr. 2015 Sep;69(9):977–1003.

60. ATS Committee on Proficiency Standards for Clinical Pulmonary Function Laboratories. ATS statement: guidelines for the six-minute walk test. Am J Respir Crit Care Med. 2002 Jul 1;166(1):111–7.

61. Waltz X, Romana M, Hardy-Dessources M-D, Lamarre Y, Divialle-Doumdo L, Petras M, et al. Hematological and hemorheological determinants of the six-minute walk test performance in children with sickle cell anemia. PloS One. 2013;8(10):e77830.

62. Borg G, Hassmén P, Lagerström M. Perceived exertion related to heart rate and blood lactate during arm and leg exercise. Eur J Appl Physiol. 1987;56(6):679–85.

63. Uyuklu M, Cengiz M, Ulker P, Hever T, Tripette J, Connes P, et al. Effects of storage duration and temperature of human blood on red cell deformability and aggregation. Clin Hemorheol Microcirc. 2009;41(4):269–78.

64. Connes P, Uyuklu M, Tripette J, Boucher JH, Beltan E, Chalabi T, et al. Sampling time after tourniquet removal affects erythrocyte deformability and aggregation measurements. Clin Hemorheol Microcirc. 2009;41(1):9–15.

65. Baskurt OK, Boynard M, Cokelet GC, Connes P, Cooke BM, Forconi S, et al. New guidelines for hemorheological laboratory techniques. Clin Hemorheol Microcirc. 2009;42(2):75–97.

66. Aleman MM, Holle LA, Stember KG, Devette CI, Monroe DM, Wolberg AS. Cystamine preparations exhibit anticoagulant activity. Miyata T, editor. PLoS One. 2015;10(4):e0124448 Available from: https://dx.plos.org/10.1371/journal. pone.0124448.

67. . Byrnes JR, Duval C, Wang Y, Hansen CE, Ahn B, Mooberry MJ, et al. Factor XIIIa-dependent retention of red blood cells in clots is mediated by fibrin α- chain crosslinking. Blood.

2015;126(16):1940–8. Available from: https://www.bloodjournal.org/content/126/16/1940.full.pdf. https://doi.org/10.1182/ blood-2015-06-652263.

16 LISTE DES ANNEXES

Annexe 1 :

MINI-MENTAL TEST DE FOLSTEIN

Score maximal	Score	
5		**ORIENTATION (1 point par réponse juste)**
	…..	- En quelle année sommes-nous ?
	…..	- Quelle saison ?
	…..	- Quel mois ?
	…..	- Quelle est la date ?
	…..	- Quel est le jour ?
5	…..	- Dans quelle pays sommes-nous ?
	…..	- Quelle ville ?
	…..	- Quel département ?
	…..	- Quel est le nom de l'hôpital ? (ou adresse du médecin)
	…..	- Quelle salle ? (ou endroit, cabinet, etc.…)

Annexe 2 :

Questionnaire du temps passé à être sédentaire

	TEMPS PASSEE PENDANT UN JOUR HABITUEL DE SEMAINE									
	Aucune	- 15 min	15-30 min	30-60 min	1-2 h	2-3 h	3-4 h	4-5 h	5-6 H	Plus de 6 h
1. Assis/e au travail	1.❑	2.❑	3.❑	4.❑	5.❑	6.❑	7.❑	8.❑	9.❑	10.❑
**Et pendant votre temps libre de loisirs**										
2. Assis/e ou allongé/e en regardant la TV	1.❑	2.❑	3.❑	4.❑	5.❑	6.❑	7.❑	8.❑	9.❑	10.❑
3. Utilisant l'ordinateur à la maison	1.❑	2.❑	3.❑	4.❑	5.❑	6.❑	7.❑	8.❑	9.❑	10.❑
4. Assis/e pour manger	1.❑	2.❑	3.❑	4.❑	5.❑	6.❑	7.❑	8.❑	9.❑	10.❑
5. Assis/e ou allongé/e en lisant ou écoutant de la musique	1.❑	2.❑	3.❑	4.❑	5.❑	6.❑	7.❑	8.❑	9.❑	10.❑
6. Assis/e avec des ami/es ou la famille	1.❑	2.❑	3.❑	4.❑	5.❑	6.❑	7.❑	8.❑	9.❑	10.❑
7. Voyageant en voiture, bus ou train	1.❑	2.❑	3.❑	4.❑	5.❑	6.❑	7.❑	8.❑	9.❑	10.❑
8. Assis/e ou allongé/e pendant ses loisirs	1.❑	2.❑	3.❑	4.❑	5.❑	6.❑	7.❑	8.❑	9.❑	10.❑
9. En faisant une sieste	1.❑	2.❑	3.❑	4.❑	5.❑	6.❑	7.❑	8.❑	9.❑	10.❑

Annexe 3 :

Activité physique			
Différents types d'AP lors d'une semaine typique. Plusieurs formes d'activités physiques doivent être prises en compte : au travail, à la maison ou au jardin, pendant les déplacements d'un endroit à un autre et pendant le temps libre (exercices physiques de loisir ou activité sportive).			
**Question**		**Ré**	**Code**
**Activités au travail = Métier: __________**			
1	Est-ce que votre travail implique des AP de FORTE intensité qui nécessitent **une augmentation conséquente de la respiration ou du rythme cardiaque**, comme [soulever charges lourdes, travailler sur un chantier, effectuer du travail de maçonnerie] pendant au moins 10 min d'affilée ?	Oui 1 Non 2 *Si Non, aller à P4*	P1
2	Habituellement, jours / semaine?	Nombre de jours └─┘	P2
3	Combien de temps / jour ?	Heures : minutes : hrs mins	P3 (a-b)
4	Est-ce que votre travail implique des AP **d'intensité** MODEREE, comme une marche rapide ou [soulever une charge légère] durant au moins 10 min d'affilée ?	Oui 1 Non 2 *Si Non, aller à P 7*	P4
5	Habituellement, jours / semaine?	Nombre de jours └─┘	P5
6	Combien de temps / jour	Heures : minutes └─┴─┘ : └─┴─┘ hrs mins	P6 (a-b)
**Se déplacer d'un endroit à l'autre**			
Maintenant, je voudrais connaître votre façon habituelle de vous déplacer d'un endroit à l'autre ; par exemple pour aller au travail, faire des courses, aller au marché, aller à votre lieu consacré au culte.			
7	Est-ce que vous effectuez des trajets d'au moins 10 minutes à pied ou à vélo ?	Oui 1 Non 2 *Si Non, aller à P 10*	P7
8	Habituellement, jours / semaine?	Nombre de jours └─┘	P8
9	Combien de temps / jour ?	Heures : minutes └─┴─┘ : └─┴─┘ hrs mins	P9 (a-b)
**Activités de loisirs**			
Maintenant je souhaiterais vous poser des questions sur **le sport, le fitness et les activités de loisirs.**			
10	Est-ce que vous pratiquez des sports, du fitness ou des activités de loisirs de **FORTE intensité** qui nécessitent une **augmentation importante de la respiration** ou du rythme cardiaque comme [courir ou jouer au football] pendant au moins 10 minutes d'affilée?	Oui 1 Non 2 *Si Non, aller à P 13*	P10
11	Habituellement, jours / semaine?	Nombre de jours └─┘	P11
12	Combien de temps / jour	Heures : minutes └─┴─┘ : └─┴─┘ hrs mins	P12 (a-b)
13	Est-ce que vous pratiquez des sports, du fitness ou des activités de loisirs d'intensité MODEREE qui nécessitent une petite augmentation de la respiration ou du rythme cardiaque comme la marche rapide [faire du vélo, nager, jouer au volley] pendant au moins dix minutes d'affilée ?	Oui 1 Non 2 *Si Non, aller à P16*	P13
14	Habituellement, jours / semaine?	Nombre de jours └─┘	[illegible]
15	Combien de temps / jour	Heures : minutes └─┴─┘ : └─┴─┘ hrs mins	P15 (a-b)

<u>Annexe 4 :</u>

3.01 Habituellement, mangez-vous du pain, des biscottes ou des céréales du type « petit déjeuner » chaque jour ?
Une seule réponse dans la colonne correspondante.
Ce groupe comprend le pain, les biscottes sous toutes leurs formes et les céréales de petit déjeuner.

❑ oui ❑ non

Si oui :
combien de fois en mangez-vous par jour ?
- ❑1 1 fois
- ❑2 2 fois
- ❑3 3 fois
- ❑4 4 fois et plus

Si non :
combien de fois en mangez-vous ?
- ❑5 4 à 6 fois par semaine
- ❑6 2 à 3 fois par semaine
- ❑7 Une fois par semaine ou moins
- ❑8 Jamais

3.02 Habituellement, mangez-vous du riz, des pâtes, des pommes de terre, de la semoule ou du maïs chaque jour ?
Une seule réponse dans la colonne correspondante.
Ce groupe comprend aussi le blé, la purée lyophilisée, les raviolis, lasagnes, hachis-Parmentier, gratins dauphinois, etc. et tous les plats cuisinés à base de riz, pâtes, pomme de terre ou semoule.

❑ oui ❑ non

Si oui :
combien de fois en mangez-vous par jour ?
- ❑1 1 fois
- ❑2 2 fois
- ❑3 3 fois
- ❑4 4 fois et plus

Si non :
combien de fois en mangez-vous ?
- ❑5 4 à 6 fois par semaine
- ❑6 2 à 3 fois par semaine
- ❑7 Une fois par semaine ou moins
- ❑8 Jamais

3.03 Habituellement, mangez-vous des légumes secs (haricots secs, lentilles, pois chiches etc.) chaque semaine ?
Une seule réponse dans la colonne correspondante.
Ce groupe comprend tous les légumes secs (pois chiches, pois cassés, flageolets, maïs, lentilles, haricots blancs, rouges, fèves) et les préparations à base de légumes secs (saucisses lentilles, couscous si il y a des pois chiches, etc.).

❑ oui ❑ non

Si oui :
combien de fois en mangez-vous par semaine ?
- ❑1 1 fois
- ❑2 2 fois
- ❑3 3 fois
- ❑4 4 fois et plus

Si non :
combien de fois en mangez-vous ?
- ❑5 2 à 3 fois par mois
- ❑6 **Une fois par mois ou moins**
- ❑7 Jamais

3.04 Habituellement, mangez-vous des produits laitiers chaque jour ?
Une seule réponse dans la colonne correspondante.
Cette section comprend le lait (aromatisé ou nature), les fromages, les yaourts (nature ou aux fruits), le fromage blanc et les petits suisses.
Les desserts lactés tels que les crèmes dessert ou les flans, ainsi que les yaourts à boire, sont compris dans les produits sucrés (question 3.10) à cause de leur teneur en sucre et en matière grasse et ne sont pas inclus dans cette question.

❑ oui ❑ non

Si oui :
combien de fois en mangez-vous par jour ?
- ❑1 1 fois
- ❑2 2 fois
- ❑3 3 fois
- ❑4 4 fois et plus

Si non :
combien de fois en mangez-vous ?
- ❑5 4 à 6 fois par semaine
- ❑6 2 à 3 fois par semaine
- ❑7 Une fois par semaine ou moins
- ❑8 Jamais

3.05 Habituellement, consommez-vous des fruits (y compris des jus de fruits 100% pur jus) chaque jour ?

Une seule réponse dans la colonne correspondante.

Cette section comprend les fruits sous toute leur forme (crus ou cuits, en compote, en conserve, surgelés, au sirop, soupes, etc.). Les jus de fruits 100% pur jus ou sans sucre ajouté, les tartes et les gâteaux à base de fruits sont compris dans cette catégorie. Ne sont pas compris les nectars, « boissons à base fruits », etc.

❑ oui ❑ non

Si oui : combien de fois en mangez-vous par jour ?	Si non : combien de fois en mangez-vous ?
❑1 1 fois	❑5 4 à 6 fois par semaine
❑2 2 fois	❑6 2 à 3 fois par semaine
❑3 3 fois	❑7 Une fois par semaine ou moins
❑4 4 fois et plus	❑8 Jamais

3.06 Habituellement, mangez-vous des légumes (sans compter les pommes de terre et les légumes secs) chaque jour ?

Une seule réponse dans la colonne correspondante.

Cette section comprend les légumes sous toute leur forme (purée, en conserve, surgelés, soupes, tartes, etc.), qu'ils soient crus ou cuits. La sauce tomate fait partie de cette catégorie, mais pas les pommes de terre.

❑ oui ❑ non

Si oui : combien de fois en mangez-vous par jour ?	Si non : combien de fois en mangez-vous ?
❑1 1 fois	❑5 4 à 6 fois par semaine
❑2 2 fois	❑6 2 à 3 fois par semaine
❑3 3 fois	❑7 Une fois par semaine ou moins
❑4 4 fois et plus	❑8 Jamais

3.07 Habituellement, mangez-vous de la viande, de la volaille, du jambon ou des œufs chaque jour ?

Une seule réponse dans la colonne correspondante.

Cette catégorie comprend toutes les viandes, les œufs sous toute leur forme, les abats, etc. Pour le jambon, cette question ne concerne que le jambon blanc (jambon cuit). Le reste de la charcuterie (le jambon cru, les saucisses, rillettes, pâtés, saucisson etc.) n'est pas compris dans cette catégorie.

❑ oui ❑ non

Si oui : combien de fois en mangez-vous par jour ?	Si non : combien de fois en mangez-vous ?
❑1 1 fois	❑5 4 à 6 fois par semaine
❑2 2 fois	❑6 2 à 3 fois par semaine
❑3 3 fois	❑7 Une fois par semaine ou moins
❑4 4 fois et plus	❑8 Jamais

3.08 Habituellement, mangez-vous du poisson ou d'autres produits de la pêche chaque semaine ?

Une seule réponse dans la colonne correspondante.

Cette catégorie comprend le poisson sous toutes ses formes (dont le poisson en conserve, le poisson pané) et les fruits de mer.

N'oubliez pas toutes les préparations à base de fruits de mer dans cette catégorie (tartes aux fruits de mer, bouchées à la reine aux fruits de mers, etc.)

❑ oui ❑ non

Si oui : combien de fois en mangez-vous par semaine ?	Si non : combien de fois en mangez-vous ?
❑1 1 fois	❑5 2 à 3 fois par mois
❑2 2 fois	❑6 Une fois par mois ou moins
❑3 3 fois	❑7 Jamais
❑4 4 fois et plus	

3.09 Habituellement, mangez-vous des plats prêts à consommer (ou plats « préparés ») qu'ils soient frais, surgelés ou en conserve chaque jour?

Une seule réponse dans la colonne correspondante.

Cette catégorie comprend par exemple les pizzas, les barquettes préparées, sous vide ou congelées, les cassoulets en conserve, les plats achetés chez le traiteur ainsi que ceux achetés en fast-food et rapportés à la maison, etc.

❑ oui ❑ non

Si oui : combien de fois en mangez-vous par jour ?	Si non : combien de fois en mangez-vous ?
❑1 1 fois	❑5 4 à 6 fois par semaine
❑2 2 fois	❑6 2 à 3 fois par semaine
❑3 3 fois	❑7 Une fois par semaine ou moins
❑4 4 fois et plus	❑8 Jamais

3.10 Habituellement, mangez-vous des produits sucrés comme des gâteaux, des barres chocolatées, des pâtisseries, des viennoiseries, des crèmes dessert, chaque jour (sans compter les boissons sucrées)?

Une seule réponse dans la colonne correspondante.

Cette catégorie comprend tous les produits sucrés quelque soit leur forme (par exemple : confiserie, barre chocolatée, flans, pain au raisin, mousse au chocolat, etc.) et leur provenance (commerce ou fait maison).

❑ oui ❑ non

Si oui : combien de fois en mangez-vous par jour ?	Si non : combien de fois en mangez-vous ?
❑1 1 fois	❑5 4 à 6 fois par semaine
❑2 2 fois	❑6 2 à 3 fois par semaine
❑3 3 fois	❑7 Une fois par semaine ou moins
❑4 4 fois et plus	❑8 Jamais

Annexe 5 :

EQ-5D-5L

Questionnaire sur la santé

Version française pour le Canada

(French version for Canada)

EuroQol Group EQ-5D

Pour chaque rubrique, veuillez cocher UNE case, celle qui décrit le mieux votre santé AUJOURD'HUI.

MOBILITÉ

Je n'ai aucun problème pour me déplacer à pied ❑

J'ai des problèmes légers pour me déplacer à pied ❑

J'ai des problèmes modérés pour me déplacer à pied ❑

J'ai des problèmes sévères pour me déplacer à pied ❑

Je suis incapable de me déplacer à pied ❑

AUTONOMIE DE LA PERSONNE

Je n'ai aucun problème pour me laver ou m'habiller tout(e) seul(e) ❑

J'ai des problèmes légers pour me laver ou m'habiller tout(e) seul(e) ❑

J'ai des problèmes modérés pour me laver ou m'habiller tout(e) seul(e) ❑

J'ai des problèmes sévères pour me laver ou m'habiller tout(e) seul(e) ❑

Je suis incapable de me laver ou de m'habiller tout(e) seul(e) ❑

ACTIVITÉS COURANTES *(exemples: travail, études, travaux domestiques, activités familiales ou loisirs)*

Je n'ai aucun problème pour accomplir mes activités courantes ❑

J'ai des problèmes légers pour accomplir mes activités courantes ❑

J'ai des problèmes modérés pour accomplir mes activités courantes ❑

J'ai des problèmes sévères pour accomplir mes activités courantes ❑

Je suis incapable d'accomplir mes activités courantes ❑

DOULEURS / INCONFORT

Je n'ai ni douleur ni inconfort ❑

J'ai des douleurs ou un inconfort léger(ères) ❑

J'ai des douleurs ou un inconfort modéré(es) ❑

J'ai des douleurs ou un inconfort sévère(s) ❑

J'ai des douleurs ou un inconfort extrême(s) ❑

ANXIÉTÉ / DÉPRESSION

Je ne suis ni anxieux(se) ni déprimé(e) ❑

Je suis légèrement anxieux(se) ou déprimé(e) ❑

Je suis modérément anxieux(se) ou déprimé(e) ❑

Je suis sévèrement anxieux(se) ou déprimé(e) ❑

Je suis extrêmement anxieux(se) ou déprimé(e) ❑

2

EuroQol Group EQ-5D

La meilleure santé que vous puissiez imaginer

- Nous aimerions savoir dans quelle mesure votre santé est bonne ou mauvaise AUJOURD'HUI.
- Cette échelle est numérotée de 0 à 100.
- 100 correspond à la meilleure santé que vous puissiez imaginer. 0 correspond à la pire santé que vous puissiez imaginer.
- Veuillez faire un X sur l'échelle afin d'indiquer votre état de santé AUJOURD'HUI.
- Maintenant, veuillez noter dans la case ci-dessous le chiffre que vous avez coché sur l'échelle.

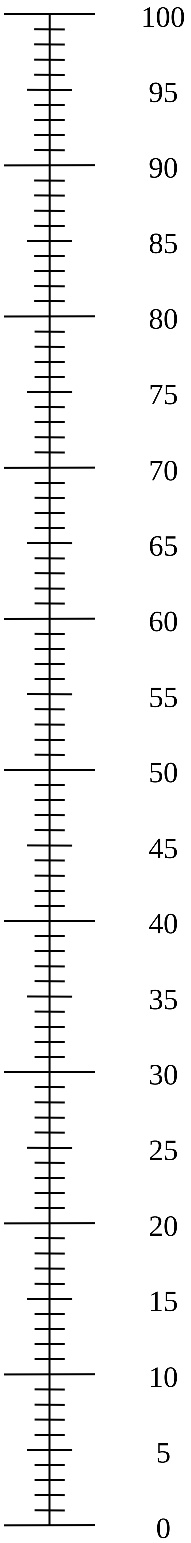

VOTRE SANTÉ AUJOURD'HUI = ☐

La pire santé que vous puissiez imaginer

3

EuroQol Group EQ-5D
