# Supplementary material for: Influence of a six-month home-based individualized physical activity intervention on carotid plaque instability measured by magnetic resonance imaging: a randomized controlled clinical trial
Source: eClinicalMedicine. 2025 Apr 22;83:103158. doi: 10.1016/j.eclinm.2025.103158 (PMC12179388; doi:10.1016/j.eclinm.2025.103158)
Supplement: Renamed_ef9bb [file mmc4.pdf]

ClinicalTrials.gov PRS
Protocol Registration and Results System

ClinicalTrials.gov Protocol Registration and Results System (PRS) Receipt
Release Date: August 9, 2019

ClinicalTrials.gov ID: NCT04053166

Study Identification

Unique Protocol ID: 69HCL19_0345

Brief Title: Individualized Physical Activity and Carotid Plaque Instability (PACAPh)

Official Title: Effect of an Individualized Home-based Physical Activity Trial on Carotid Plaque Vulnerability for Asymptomatic Patients

Secondary IDs: 2019-A01543-54 [ID-RCB]

Study Status

Record Verification: August 2019

Overall Status: Not yet recruiting

Study Start: October 1, 2019 [Anticipated]

Primary Completion: October 1, 2021 [Anticipated]

Study Completion: October 1, 2021 [Anticipated]

Sponsor/Collaborators

Sponsor: Hospices Civils de Lyon

Responsible Party: Sponsor

Collaborators:

Oversight

U.S. FDA-regulated Drug: No

U.S. FDA-regulated Device: No

U.S. FDA IND/IDE: No

Human Subjects Review: Board Status: Pending
Board Name: CPP Sud-Méditerranée II
Board Affiliation: Direction Générale de la Santé
Phone: 04 91 74 56 09
Email: secretaires@cpp-sudmed2.fr
Address:

Hôpital Sainte Marguerite
Pavillon 9
1er étage
270, bld Sainte Marguerite
13274 MARSEILLE Cedex 09

Data Monitoring: No

FDA Regulated Intervention: No

Study Description

Brief Summary: Intraplaque hemorrhage (IPH) is one of the main features of the carotid plaque instability's and predictor of ischemic stroke. Benefits (on the basis on benefit/risk ratio) of the carotid endarterectomy remain unclear for stroke asymptomatic patients; thus, more and more patients with important stenosis (i.e. over 60%) detected are not operated. However, these patients need adapted therapeutic treatments to limit plaque instability and this should include physical activity (PA). Indeed, PA has been showed to decrease numerous inflammatory markers involved in atherosclerosis. It has also recently been reported on stroke asymptomatic patients that the prevalence of carotid IPH was decreased in those with higher level of PA. Magnetic Resonance Imaging (MRI) of the IPH has been shown to be the better non-invasive imaging technique to assess carotid plaque instability and in particular IPH. Here, the aim of this study is to assess the effect of an individualized home-based 6 months physical activity intervention on carotid IPH and other biomarkers of vulnerability for asymptomatic patients.

This study has been designed as a monocentric, longitudinal and interventional study. This study will involve one centre: Hopital Louis Pradel (HCL, Lyon). After inclusion tests, patients will be randomly included in the control group, or in the PA group. Patients of the PA group will have connected bracelets to measure daily count of steps. Twice a month, daily goals will be revaluated to increase or maintain the steps per day. The final goal is to reach 6 000 steps per day or increase by 30% the initial count of steps per day. Same tests will be done after 6 months of intervention for comparison.

Detailed Description:

Conditions

Conditions: Carotid Atherosclerosis

Keywords: Intraplaque haemorrhage
MRI
home-based Physical activity
carotic plaque vulnerability

Study Design

Study Type: Interventional

Primary Purpose: Prevention

Study Phase: N/A

Interventional Study Model: Parallel Assignment

Number of Arms: 2

Masking: Single (Outcomes Assessor)
Two experienced observers will blindly read the MRI scans.

Allocation: Randomized

Enrollment: 80 [Anticipated]

Arms and Interventions

Arms	Assigned Interventions
Experimental: Individualized home-based physical activity<br>The subjects of this arm will have a daily goal in number of steps based on the initial 2 first week evaluation of daily number of steps. They will wear connected wrists, and will be contacted twice a month by phone call by the adapted physical activity trainer to revaluate these goals.	Device: individualized home-based physical activity<br>Subjects will have to reach a daily goal in number of steps, based on the initial evaluation, during 6 months . They will wear connected wrists, and will be contacted twice a month by phone call by an adapted physical activity to revaluate these goals.<br>MRI<br>An MRI will be performed for each patient at the end of the study to identify IPH and other features of histological vulnerability (lipid core, fibrous cap integrity and calcifications).<br>Biological/Vaccine: blood sampling<br>Blood will be collected, to analyse monocyte phenotype by flow cytometry, blood rheology by ektacytometry, coagulation by rotational thromboelastometry (ROTEM). Plasma will be extracted from blood to assess inflammation, oxidative stress and antioxidant markers.<br>Questionnaires<br>sedentary, physical activity, nutrition and quality of life questionnaire will be performed fo each patient.<br>6-minute walk test<br>The 6-minute walk test is a simple, individualized test that measures how fast a patient walks on a flat, hard surface for 6 minutes.
Active Comparator: Control group<br>The subjects of this arm will not have evaluation of daily steps and recommendations regarding physical activity and sedentary behaviour. They will be asked to live as usual.	MRI<br>An MRI will be performed for each patient at the end of the study to identify IPH and other features of histological vulnerability (lipid core, fibrous cap integrity and calcifications).<br>Biological/Vaccine: blood sampling<br>Blood will be collected, to analyse monocyte phenotype by flow cytometry, blood rheology by ektacytometry, coagulation by rotational thromboelastometry (ROTEM). Plasma will be extracted from blood to assess inflammation, oxidative stress and antioxidant markers.<br>Questionnaires<br>sedentary, physical activity, nutrition and quality of life questionnaire will be performed fo each patient.<br>6-minute walk test<br>The 6-minute walk test is a simple, individualized test that measures how fast a patient walks on a flat, hard surface for 6 minutes.

Outcome Measures

Primary Outcome Measure:

1. decreased intensity of IPH levels measured by MRI
   Image quality will be assessed from 1 to 5 (grade 1, low Signal-to-Noise Ratio (SNR) limits use, arterial wall and vessel margins are unidentifiable; grade 2, marginal SNR, arterial wall is visible, but the substructure, lumen, and outer boundaries are indistinct; grade 3, marginal SNR, wall structures are identifiable, but lumen and outer boundaries are partially obscured; grade 4, high SNR with minimal artifacts, vessel wall, lumen, and adventitial margins are well defined; and grade 5, high SNR without artifacts, wall architecture depicted in detail, lumen and adventitial boundary are clearly defined) . If the quality of the image is sufficient (≥ 3), IPH levels will be semi-quantified on a scale from 0 to

3 (0: No IPH, 1: light IPH, 2 moderate IPH, strong IPH). Images will be assessed blindly and independently by clinical experts of carotid plaque imaging.

[Time Frame: Day 0]

2. decreased intensity of IPH levels measured by MRI
   Image quality will be assessed from 1 to 5 (grade 1, low Signal-to-Noise Ratio (SNR) limits use, arterial wall and vessel margins are unidentifiable; grade 2, marginal SNR, arterial wall is visible, but the substructure, lumen, and outer boundaries are indistinct; grade 3, marginal SNR, wall structures are identifiable, but lumen and outer boundaries are partially obscured; grade 4, high SNR with minimal artifacts, vessel wall, lumen, and adventitial margins are well defined; and grade 5, high SNR without artifacts, wall architecture depicted in detail, lumen and adventitial boundary are clearly defined) . If the quality of the image is sufficient (≥ 3), IPH levels will be semi-quantified on a scale from 0 to 3 (0: No IPH, 1: light IPH, 2 moderate IPH, strong IPH). Images will be assessed blindly and independently by clinical experts of carotid plaque imaging.

   [Time Frame: Month 6]

Secondary Outcome Measure:

3. Evaluation of intermediate monocyte phenotype (cluster of differentiation 14 (CD14)++ /cluster of differentiation 16 (CD16)+)
   monocytes will be extracted from blood sample, marked with specific antibodies (anti CD14/16) and intermediate phenotypes (in %) will be measured by flow cytometry

   [Time Frame: Day 0]

4. Evaluation of intermediate monocyte phenotype (cluster of differentiation 14 (CD14)++ /cluster of differentiation 16 (CD16)+)
   monocytes will be extracted from blood sample, marked with specific antibodies (anti CD14/16) and intermediate phenotypes (in %) will be measured by flow cytometry

   [Time Frame: Month 6]

5. Evaluation of classical monocyte phenotype (cluster of differentiation 14 (CD14)++ /cluster of differentiation 16 (CD16)-)
   monocytes will be extracted from blood sample, marked with specific antibodies (anti CD14/16) and classical phenotypes (in %) will be measured by flow cytometry

   [Time Frame: Day 0]

6. Evaluation of classical monocyte phenotype (cluster of differentiation 14 (CD14)++ /cluster of differentiation 16 (CD16)-)
   monocytes will be extracted from blood sample, marked with specific antibodies (anti CD14/16) and classical phenotypes (in %) will be measured by flow cytometry

   [Time Frame: Month 6]

7. Evaluation of non-classical monocyte phenotype (cluster of differentiation 14 (CD14)+ /cluster of differentiation 16 (CD16)++)
   monocytes will be extracted from blood sample, marked with specific antibodies (anti CD14/16) and non-classical phenotypes (in %) will be measured by flow cytometry

   [Time Frame: Day 0]

8. Evaluation of non-classical monocyte phenotype (cluster of differentiation 14 (CD14)+ /cluster of differentiation 16 (CD16)++)
   monocytes will be extracted from blood sample, marked with specific antibodies (anti CD14/16) and non-classical phenotypes (in %) will be measured by flow cytometry

   [Time Frame: Month 6]

9. Assessment of red blood cell aggregation
   Red blood cell aggregation (in %)will be measured by ektacytometry

   [Time Frame: Day 0]

10. Assessment of red blood cell aggregation
    Red blood cell aggregation (in %) will be measured by ektacytometry

[Time Frame: Month 6]

11. in vitro clotting formation time
 In vitro clotting formation time (in minutes) will be measured on whole blood by rotational thromboelastometry

 [Time Frame: Day 0]
12. in vitro clotting formation time
 In vitro clotting formation time (in minutes) will be measured on whole blood by rotational thromboelastometry

 [Time Frame: Month 6]
13. Measurement of in vitro clot lysis index
 In vitro clot lysis index (in millimeter) will be measured on whole blood by rotational thromboelastometry

 [Time Frame: Day 0]
14. Measurement of in vitro clot lysis index
 In vitro clot lysis index (in millimeter) will be measured on whole blood by rotational thromboelastometry

 [Time Frame: Month 6]
15. Measurement of in vitro clot firmness
 In vitro clot firmness (in millimeter) will be measured on whole blood by rotational thromboelastometry

 [Time Frame: Day 0]
16. Measurement of in vitro clot firmness
 In vitro clot firmness (in millimeter) will be measured on whole blood by rotational thromboelastometry

 [Time Frame: Month 6]
17. Assessment of plasma lipid oxidation
 Plasma protein oxidation (advanced oxidation proteins products) measured by by spectrophotometry (in micromole/liter (µmol/L))

 [Time Frame: Day 0]
18. Assessment of plasma lipid oxidation
 Plasma protein oxidation (advanced oxidation proteins products) measured by by spectrophotometry (in micromole/liter (µmol/L))

 [Time Frame: Month 6]
19. Assessment of plasma protein oxidation
 Plasma protein oxidation (advanced oxidation proteins products) measured by by spectrophotometry (in micromole/liter (µmol/L))

 [Time Frame: Day 0]
20. Assessment of plasma protein oxidation
 Plasma protein oxidation (advanced oxidation proteins products) measured by by spectrophotometry (in micromole/liter (µmol/L))

 [Time Frame: Month 6]
21. Assessment of plasma protein nitration
 Plasma protein nitration (nitrotyrosine) measured by the enzyme-linked immunosorbent assay (ELISA) in micromole/liter (µmol/L).

 [Time Frame: Day 0]
22. Assessment of plasma protein nitration
 Plasma protein nitration (nitrotyrosine) measured by the enzyme-linked immunosorbent assay (ELISA) in micromole/liter (µmol/L).

 [Time Frame: Month 6]
23. Assessment of plasma inflammatory markers
 Plasma inflammatory markers will be measured by multiplex assay in micromole/liter (µmol/L).

 [Time Frame: Day 0]

24. Assessment of plasma inflammatory markers
    Plasma inflammatory markers will be measured by multiplex assay in micromole/liter (μmol/L).

    [Time Frame: Month 6]

25. Assessment of plasma enzymes activity
    Plasma antioxidant enzymes activity will be measured by enzymology (in micromole/liter/minute (μmol/L/min))

    [Time Frame: Day 0]

26. Assessment of plasma enzymes activity
    Plasma antioxidant enzymes activity will be measured by enzymology (in micromole/liter/minute (μmol/L/min))

    [Time Frame: Month 6]

27. number of steps per day
    the daily number of steps (in number of step per day) will be measured using a connected wrist activity tracker

    [Time Frame: during 2 weeks after Day 0]

28. number of steps per day
    the daily number of steps (in number of step per day) will be measured using a connected wrist activity tracker

    [Time Frame: during 2 weeks after Month 6]

29. distance of the 6 minutes walking test
    The distance at the 6 minutes walking test (in meters) will be evaluated on the 30meters flat round-trip

    [Time Frame: Day 0]

30. distance of the 6 minutes walking test
    The distance at the 6 minutes walking test (in meters) will be evaluated on the 30meters flat round-trip

    [Time Frame: Month 6]

31. quadriceps maximal isometric strength
    The quadriceps maximal isometric strength (in Newton) will be evaluated in sitting position using dynamometer

    [Time Frame: Day 0]

32. quadriceps maximal isometric strength
    The quadriceps maximal isometric strength (in Newton) will be evaluated in sitting position using dynamometer

    [Time Frame: Month 6]

33. Determination of the level of physical activity
    the level physical activity will be evaluated by the global physical activity questionnaire (in Metabolic Equivalent of Task/minutes per week (MET/min.week)).

    [Time Frame: Day 0]

34. Determination of the level of physical activity
    the level physical activity will be evaluated by the global physical activity questionnaire (in Metabolic Equivalent of Task/minutes per week (MET/min.week)).

    [Time Frame: Month 6]

35. Determination of the sedentary time
    Sedentary time will be evaluated by the sedentary behaviour questionnaire evaluating the total daily sitting and lying down time (in minute/day) during awaking time.

    [Time Frame: Day 0]

36. Determination of the sedentary time
    Sedentary time will be evaluated by the sedentary behaviour questionnaire evaluating the total daily sitting and lying down time (in minute/day) during awaking time.

    [Time Frame: Month 6]

37. descriptive health state score
    Health state score will be assessed using descriptive system of the EQ-5D-5L (five-level version of the EuroQol five-dimensional) questionnaire.

Health status is measured in terms of five dimensions (5D); mobility, self-care, usual activities, pain/discomfort, and anxiety/depression. The subjects self-rate their level of severity for each dimension using a five-level (EQ-5D-5L) scale (scored from 1 to 5, 1 indicating no problem and 5 indicating extreme problem).

The health rate score correspond to the addition of each dimension score and is from 5 to 25. The lower the score, the better the health state.

[Time Frame: Day 0]

38. descriptive health state score
    Health state score will be assessed using descriptive system of the EQ-5D-5L (five-level version of the EuroQol five-dimensional) questionnaire.

    Health status is measured in terms of five dimensions (5D); mobility, self-care, usual activities, pain/discomfort, and anxiety/depression. The subjects self-rate their level of severity for each dimension using a five-level (EQ-5D-5L) scale (scored from 1 to 5, 1 indicating no problem and 5 indicating extreme problem).

    The health rate score correspond to the addition of each dimension score and is from 5 to 25. The lower the score, the better the health state.

    [Time Frame: Month 6]

39. self-evaluated overall health status
    Overall health status will be assessed using the evaluation part of the EQ-5D-5L (five-level version of the EuroQol five-dimensional) questionnaire.

    The subject’s self- evaluate their overall health status using the visual analogue scale (EQ-VAS).

    The raw score is from 0 to 100. The higher the score, the better the perceived overall health status

    [Time Frame: Day 0]

40. self-evaluated overall health status
    Overall health status will be assessed using the evaluation part of the EQ-5D-5L (five-level version of the EuroQol five-dimensional) questionnaire.

    The subject’s self- evaluate their overall health status using the visual analogue scale (EQ-VAS).

    The raw score is from 0 to 100. The higher the score, the better the perceived overall health status

    [Time Frame: Month 6]

41. body mass index
    Body mass index (in kilogram/metre² (kg/m²)) will be calculated with the measurement of body weight (in kilogram) and height (in meter)

    [Time Frame: Day 0]

42. body mass index
    Body mass index (in kilogram/metre² (kg/m²)) will be calculated with the measurement of body weight (in kilogram) and height (in meter)

    [Time Frame: Month 6]

43. number of comorbidities
    Number of comorbidities (Diabetes, hypertension, obesity and , poly-atheroma) will be determined

    [Time Frame: Day 0]

44. number of comorbidities
    Number of comorbidities (Diabetes, hypertension, obesity and , poly-atheroma) will be determined

    [Time Frame: Month 6]

Eligibility

Minimum Age: 18 Years

Maximum Age:

Sex: All

Gender Based: No

Accepts Healthy Volunteers: No

Criteria: Inclusion Criteria:

- Patient with an carotid atheromatous plaque with ≥ 50% North American Symptomatic Carotid Endarterectomy Trial (NASCET) stenosis
- Patient from vascular surgery department of the Louis Pradel Hospital of the Hospices Civils de Lyon, but not operated
- Males and females aged over 18 years old
- No contra-indication to physical activity with index performance (PS) < 2
- Available and voluntary to invest in the study throughout its duration (6 months)
- Able to understand, read and write French;
- a social security system or similar;
- Having dated and signed informed consent.

Exclusion Criteria:

- Transient ischemic attack (TIA) or ipsilateral cerebral infarction less than 6 months
- History of ipsilateral carotid surgery or cervical irradiation;
- Cancer, heart failure, seropositivity;
- Coronary risk;
- Renal failure (Cockcroft clearance of creatinine < 30 milliliter/minute (mL/min);
- Contraindication and precautions for use related to Prohance: hypersensitivity to the active substance or to any of the constituents of Prohance, renal insufficiency with clearance <30 ml / min / 1.73 m², probability of convulsions during the higher examination in patients with epilepsy or brain injury, pregnancy, breastfeeding;
- Contraindication to MRI: ferromagnetic material (including pacemaker, implantable defibrillators, cardiac valve prostheses, cochlear implants, neurostimulators, implanted automated injection equipment, intraocular metallic foreign bodies, neurosurgical and vascular clips);
- Carotid occlusion;
- ipsilateral intracranial stenosis;
- Risk of pregnancy or proven pregnancy on interrogation data. Breastfeeding;
- Patient under guardianship, under curatorship or safeguard of justice;
- inability to express consent;
- uncontrolled cardiological or neurological diseases;
- Impossibility of being followed for medical, social, geographical or psychological reasons throughout the duration of the study.

Contacts/Locations

Central Contact Person: Antoine MILLON, PU, PH
Telephone: 04 72 11 11 16 Ext. +33
Email: antoine.millon@chu-lyon.fr

Central Contact Backup:

Study Officials:

Locations: **France**
Hôpital Louis Pradel
Bron, France, 69500

Contact: Antoine Millon, PU, PH 04.72.11.11.16 Ext. +33
antoine.millon@chu-lyon.fr
Principal Investigator: Antoine Millon, PU, PH

IPDSharing

Plan to Share IPD:

References

Citations:

Links:

Available IPD/Information:

U.S. National Library of Medicine | U.S. National Institutes of Health | U.S. Department of Health & Human Services
